# Meta-analysis of gene–environment-wide association scans accounting for education level identifies additional loci for refractive error

**DOI:** 10.1038/ncomms11008

**Published:** 2016-03-29

**Authors:** Qiao Fan, Virginie J. M. Verhoeven, Robert Wojciechowski, Veluchamy A. Barathi, Pirro G. Hysi, Jeremy A. Guggenheim, René Höhn, Veronique Vitart, Anthony P. Khawaja, Kenji Yamashiro, S Mohsen Hosseini, Terho Lehtimäki, Yi Lu, Toomas Haller, Jing Xie, Cécile Delcourt, Mario Pirastu, Juho Wedenoja, Puya Gharahkhani, Cristina Venturini, Masahiro Miyake, Alex W. Hewitt, Xiaobo Guo, Johanna Mazur, Jenifer E. Huffman, Katie M. Williams, Ozren Polasek, Harry Campbell, Igor Rudan, Zoran Vatavuk, James F. Wilson, Peter K. Joshi, George McMahon, Beate St Pourcain, David M. Evans, Claire L. Simpson, Tae-Hwi Schwantes-An, Robert P. Igo, Alireza Mirshahi, Audrey Cougnard-Gregoire, Céline Bellenguez, Maria Blettner, Olli Raitakari, Mika Kähönen, Ilkka Seppala, Tanja Zeller, Thomas Meitinger, Consortium for Refractive Error and Myopia (CREAM), Janina S. Ried, Christian Gieger, Laura Portas, Elisabeth M. van Leeuwen, Najaf Amin, André G. Uitterlinden, Fernando Rivadeneira, Albert Hofman, Johannes R. Vingerling, Ya Xing Wang, Xu Wang, Eileen Tai-Hui Boh, M. Kamran Ikram, Charumathi Sabanayagam, Preeti Gupta, Vincent Tan, Lei Zhou, Candice E. H. Ho, Wan'e Lim, Roger W. Beuerman, Rosalynn Siantar, E-Shyong Tai, Eranga Vithana, Evelin Mihailov, Chiea-Chuen Khor, Caroline Hayward, Robert N. Luben, Paul J. Foster, Barbara E. K. Klein, Ronald Klein, Hoi-Suen Wong, Paul Mitchell, Andres Metspalu, Tin Aung, Terri L. Young, Mingguang He, Olavi Pärssinen, Cornelia M. van Duijn, Jie Jin Wang, Cathy Williams, Jost B. Jonas, Yik-Ying Teo, David A. Mackey, Konrad Oexle, Nagahisa Yoshimura, Andrew D. Paterson, Norbert Pfeiffer, Tien-Yin Wong, Paul N. Baird, Dwight Stambolian, Joan E. Bailey Wilson, Ching-Yu Cheng, Christopher J. Hammond, Caroline C. W. Klaver, Seang-Mei Saw, Jugnoo S. Rahi, Jean-François Korobelnik, John P. Kemp, Nicholas J. Timpson, George Davey Smith, Jamie E. Craig, Kathryn P. Burdon, Rhys D. Fogarty, Sudha K. Iyengar, Emily Chew, Sarayut Janmahasatian, Nicholas G. Martin, Stuart MacGregor, Liang Xu, Maria Schache, Vinay Nangia, Songhomitra Panda-Jonas, Alan F. Wright, Jeremy R. Fondran, Jonathan H. Lass, Sheng Feng, Jing Hua Zhao, Kay-Tee Khaw, Nick J. Wareham, Taina Rantanen, Jaakko Kaprio, Chi Pui Pang, Li Jia Chen, Pancy O. Tam, Vishal Jhanji, Alvin L. Young, Angela Döring, Leslie J. Raffel, Mary-Frances Cotch, Xiaohui Li, Shea Ping Yip, Maurice K.H. Yap, Ginevra Biino, Simona Vaccargiu, Maurizio Fossarello, Brian Fleck, Seyhan Yazar, Jan Willem L. Tideman, Milly Tedja, Margaret M. Deangelis, Margaux Morrison, Lindsay Farrer, Xiangtian Zhou, Wei Chen, Nobuhisa Mizuki, Akira Meguro, Kari Matti Mäkelä

**Affiliations:** 1 Singapore Eye Research Institute, Singapore National Eye Centre, Singapore 169856, Singapore; 2 Duke-NUS Medical School, Singapore 169857, Singapore; 3 Department of Ophthalmology, Erasmus Medical Center, 3000 CA Rotterdam, The Netherlands; 4 Department of Epidemiology, Erasmus Medical Center, 3000 CA Rotterdam, The Netherlands; 5 Computational and Statistical Genomics Branch, National Human Genome Research Institute, National Institutes of Health, Baltimore, Maryland 21224, USA; 6 Department of Epidemiology, Johns Hopkins Bloomberg School of Public Health, Baltimore, Maryland 20205, USA; 7 Department of Ophthalmology, National University Health Systems, National University of Singapore Singapore 119228, Singapore; 8 Department of Twin Research and Genetic Epidemiology, King's College London School of Medicine, London SE1 7EH, UK; 9 School of Optometry and Vision Sciences, Cardiff University, Cardiff CF24 4HQ, UK; 10 Department of Ophthalmology, University Medical Center Mainz, 55131 Mainz, Germany; 11 Department of Ophthalmology, Inselspital, University Hospital Bern, CH-3010 Bern, Switzerland; 12 Medical Research Council Human Genetics Unit, Institute of Genetics and Molecular Medicine, University of Edinburgh, Edinburgh EH4 2XU, Scotland; 13 Department of Public Health and Primary Care, Institute of Public Health, University of Cambridge School of Clinical Medicine, Cambridge CB2 0SR, UK; 14 Department of Ophthalmology and Visual Sciences, Kyoto University Graduate School of Medicine, Kyoto 6068507, Japan; 15 Program in Genetics and Genome Biology, The Hospital for Sick Children and Institute for Medical Sciences, University of Toronto, Toronto Ontario, Canada M5G 1X8; 16 Department of Clinical Chemistry, Fimlab Laboratories and School of Medicine, University of Tampere, Tampere 33520, Finland; 17 Statistical Genetics Laboratory, QIMR Berghofer Medical Research Institute, Herston, Brisbane, Queensland 4029, Australia; 18 Estonian Genome Center, University of Tartu, Tartu 51010, Estonia; 19 Centre for Eye Research Australia (CERA), Royal Victorian Eye and Ear Hospital, University of Melbourne, Melbourne, Victoria 3002, Australia; 20 Université de Bordeaux, ISPED (Institut de Santé Publique d'Épidémiologie et de Développement), Bordeaux 33000, France; 21 INSERM, U1219-Bordeaux Population Health Research Center, Bordeaux 33000, France; 22 Institute of Population Genetics, National Research Council, Sassari 07100, Italy; 23 Department of Public Health, University of Helsinki, Helsinki 00014, Finland; 24 Department of Ophthalmology, University of Helsinki and Helsinki University Hospital, Helsinki 00014, Finland; 25 UCL Institute of Ophthalmology, London SE1 7EH, UK; 26 Menzies Research Institute Tasmania, University of Tasmania, Hobart, Tasmania 7000, Australia; 27 Department of Statistical Science, School of Mathematics and Computational Science, Sun Yat-Sen University, Guangzhou 510275, China; 28 Institute of Medical Biostatistics, Epidemiology and Informatics, University Medical Center Mainz, 55131 Mainz, Germany; 29 Department of Ophthalmology, King's College London, London SE1 7EH, UK; 30 Faculty of Medicine, University of Split, Split 21000, Croatia; 31 Usher Institute for Population Health Sciences and Informatics, University of Edinburgh, Teviot Place, Edinburgh EH8 9AG, Scotland; 32 Department of Ophthalmology, Sisters of Mercy University Hospital, Zagreb 10000, Croatia; 33 MRC Integrative Epidemiology Unit (IEU), University of Bristol, Bristol BS8 2BN, UK; 34 School of Social and Community Medicine, University of Bristol, Bristol BS8 2BN, UK; 35 Max Planck Institute for Psycholinguistics, Wundtlaan 1, 6525 XD Nijmegen, The Netherlands; 36 University of Queensland Diamantina Institute, Translational Research Institute, Brisbane, Queensland 4102, Australia; 37 Department of Genetics, Genomics and Informatics, University of Tennessee Health Science Center, Memphis, Tennessee 38163, USA; 38 Department of Epidemiology and Biostatistics, Case Western Reserve University, Cleveland, Ohio 44106, USA; 39 Dardenne Eye Hospital, Bonn-Bad Godesberg, 53177 Bonn, Germany; 40 Inserm, U1167, Lille 59000, France; 41 Univ. Lille, U1167, Lille 59000, France; 42 Université Lille 2, Lille 59000, France; 43 Research Centre of Applied and Preventive Medicine, University of Turku, Turku 20520, Finland; 44 Department of Clinical Physiology and Nuclear Medicine, Turku University Hospital, Turku 20520, Finland; 45 Department of Clinical Physiology, Tampere University Hospital and School of Medicine, University of Tampere, Tampere 33520, Finland; 46 Clinic for General and Interventional Cardiology, University Heart Center Hamburg, 20246 Hamburg, Germany; 47 Institute of Human Genetics, Helmholtz Zentrum München, 85764 Neuherberg, Germany; 48 Institute of Human Genetics, Klinikum rechts der Isar, Technische Universität München, 81675 Munich, Germany; 49 Institute of Genetic Epidemiology, Helmholtz Zentrum München—German Research Center for Environmental Health, 85764 Neuherberg, Germany; 50 Department of Internal Medicine, Erasmus Medical Center, 3000 CA Rotterdam, The Netherlands; 51 Netherlands Consortium for Healthy Ageing, Netherlands Genomics Initiative, 2518 AD Hague, The Netherlands; 52 Beijing Institute of Ophthalmology, Beijing Tongren Hospital, Capital Medical University, Beijing 100044, China; 53 Saw Swee Hock School of Public Health, National University of Singapore and National University Health Systems, Singapore 117549, Singapore; 54 National Healthcare Group Eye Institute, Tan Tock Seng Hospital, Singapore 308433, Singapore; 55 Department of Medicine, National University of Singapore, Singapore 119228, Singapore; 56 Division of Human Genetics, Genome Institute of Singapore, Singapore 138672, Singapore; 57 Division of Genetics and Epidemiology, UCL Institute of Ophthalmology, London EC1V 9EL, UK; 58 NIHR Biomedical Research Centre, Moorfields Eye Hospital NHS Foundation Trust and UCL Institute of Ophthalmology, London EC1V 2PD, UK; 59 Department of Ophthalmology and Visual Sciences, University of Wisconsin School of Medicine and Public Health, Madison, Wisconsin 53726, USA; 60 Department of Ophthalmology, Centre for Vision Research, Westmead Institute for Medical Research, University of Sydney, Sydney, New South Wales 2145, Australia; 61 Department of Ophthalmology and Visual Sciences, School of Medicine and Public Health, University of Wisconsin, Madison, Wisconsin 53705, USA; 62 State Key Laboratory of Ophthalmology, Zhongshan Ophthalmic Center, Sun Yat-Sen University, Guangzhou 510060, China; 63 Department of Ophthalmology, Central Hospital of Central Finland, Jyväskylä 40620, Finland; 64 Gerontology Research Center and Department of Health Sciences, University of Jyväskylä, Jyväskylä 40014, Finland; 65 Medical Faculty Mannheim, Department of Ophthalmology, Ruprecht-Karls-University Heidelberg, 69115 Mannheim, Germany; 66 Department of Statistics and Applied Probability, National University of Singapore, Singapore 117546, Singapore; 67 Centre for Ophthalmology and Visual Science, Lions Eye Institute, University of Western Australia, Perth, Western Australia 6009, Australia; 68 Department of Ophthalmology, University of Pennsylvania, Philadelphia, Pennsylvania 19104, USA; 69 Medical Research Council Centre of Epidemiology for Child Health, Institute of Child Health, University College London, London WC1E 6BT, UK; 70 Institute of Ophthalmology, Moorfields Eye Hospital, London EC1V 2PD, UK; 71 Ulverscroft Vision Research Group, University College London, London WC1E 6BT, UK; 72 Université de Bordeaux, 33400 Talence, France; 73 INSERM (Institut National de la Santé Et de la Recherche Médicale), ISPED (Institut de Santé Publique d'épidémiologie et de Développement), Centre INSERM U897-Epidemiologie-Biostatistique, 33076 Bordeaux, France; 74 MRC Integrative Epidemiology Unit (IEU), The University of Bristol, Bristol BS8 2BN, UK; 75 Department of Ophthalmology, Flinders University, Adelaide, South Australia 5001, Australia; 76 Department of Epidemiology and Biostatistics, CaseWestern Reserve University, Cleveland, Ohio 44106, USA; 77 Department of Ophthalmology and Visual Sciences, Case Western Reserve University and University Hospitals Eye Institute, Cleveland, Ohio 44106, USA; 78 Department of Genetics, Case Western Reserve University, Cleveland, Ohio 44106, USA; 79 National Eye Institute, National Institutes of Health, Bethesda, Maryland 20892, USA; 80 Genetic Epidemiology Laboratory, QIMR Berghofer Medical Research Institute, Herston, Brisbane, Queensland 4029, Australia; 81 Suraj Eye Institute, Nagpur, Maharashtra 440001, India; 82 Department of Pediatric Ophthalmology, Duke Eye Center For Human Genetics, Durham, North Carolina 27710, USA; 83 MRC Epidemiology Unit, Institute of Metabolic Sciences, University of Cambridge, Cambridge CB2 1TN, UK; 84 MRC Epidemiology Unit, Institute of Metabolic Science, Addenbrooke's Hospital, Cambridge CB2 0QQ, UK; 85 Gerontology Research Center, University of Jyväskylä, Jyväskylä Finland; 86 Department of Public Health, University of Helsinki, Helsinki 00014, Finland; 87 Institute for Molecular Medicine, University of Helsinki, Helsinki 00014, Finland; 88 Department of Mental Health and Alcohol Abuse Services, National Institute for Health and Welfare, Helsinki 00271, Finland; 89 Department of Ophthalmology and Visual Sciences, Hong Kong Eye Hospital, The Chinese University of Hong Kong, Kowloon, Hong Kong; 90 Department of Ophthalmology and Visual Sciences, Prince of Wales Hospital, The Chinese University of Hong Kong, Shatin, Hong Kong; 91 Institute of Epidemiology I, Helmholtz Zentrum München, German Research Center for Environmental Health, 85764 Neuherberg, Germany; 92 Institute of Epidemiology II, Helmholtz Zentrum München, German Research Center for Environmental Health, 85764 Neuherberg, Germany; 93 Medical Genetics Institute, Cedars-Sinai Medical Center, Los Angeles, California 90048, USA; 94 Division of Epidemiology and Clinical Applications, National Eye Institute, Bethesda, Maryland 20892, USA; 95 Institute for Translational Genomics and Population Sciences, Los Angeles Biomedical Research Institute, Harbor-UCLA Medical Center, Los Angeles, California 90502, USA; 96 Department of Health Technology and Informatics, The Hong Kong Polytechnic University, Hong Kong, Hong Kong; 97 Institute of Molecular Genetics, National Research Council, Pavia 27100, Italy; 98 Princess Alexandra Eye Pavilion, Edinburgh EH3 9HA, UK; 99 Department of Ophthalmology and Visual Sciences, Moran Eye Center, University of Utah, Salt Lake City, Utah 84132, USA; 100 Department of Ophthalmology and Visual Sciences, John Moran Eye Center, University of Utah, Salt Lake City, Utah 84132, USA; 101 Departments of Medicine (Biomedical Genetics), Ophthalmology, Neurology, Epidemiology and Biostatistics, Boston University Schools of Medicine and Public Health, Boston, Massachusetts 02118, USA; 102 School of ophthalmology and optometry, Wenzhou Medical University, Wenzhou 325035, China; 103 Department of Ophthalmology, Yokohama City University School of Medicine, Yokohama, Kanagawa 236-0027, Japan; 104 Department of Clinical Chemistry, Fimlab Laboratories and School of Medicine, University of Tampere, Tampere 33014, Finland.

## Abstract

Myopia is the most common human eye disorder and it results from complex genetic and environmental causes. The rapidly increasing prevalence of myopia poses a major public health challenge. Here, the CREAM consortium performs a joint meta-analysis to test single-nucleotide polymorphism (SNP) main effects and SNP × education interaction effects on refractive error in 40,036 adults from 25 studies of European ancestry and 10,315 adults from 9 studies of Asian ancestry. In European ancestry individuals, we identify six novel loci (*FAM150B-ACP1*, *LINC00340*, *FBN1*, *DIS3L-MAP2K1*, *ARID2-SNAT1* and *SLC14A2*) associated with refractive error. In Asian populations, three genome-wide significant loci *AREG*, *GABRR1* and *PDE10A* also exhibit strong interactions with education (*P*<8.5 × 10^−5^), whereas the interactions are less evident in Europeans. The discovery of these loci represents an important advance in understanding how gene and environment interactions contribute to the heterogeneity of myopia.

## Abstract (web summary)

This report by the Consortium for Refractive Error and Myopia uses gene-environment-wide interaction study (GEWIS) to identify genetic loci that affect environmental influence in myopia development, and identifies ethnic specific genetic loci that attribute to eye refractive errors.

## Graphical abstract

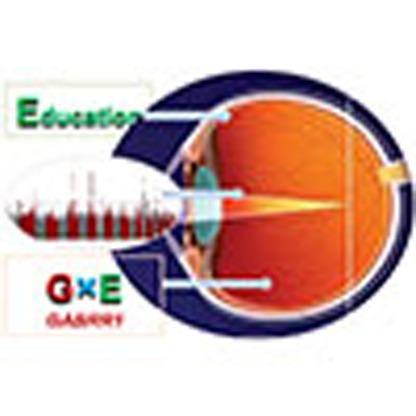

Myopia, or nearsightedness, has rapidly emerged as a global health concern in the last three decades^1^. It is one of the leading causes of visual impairment and is associated with potentially blinding ocular complications including myopic maculopathy, glaucoma, cataract and retinal detachment^2^. Evidence from family and twin studies strongly supports the heritability of myopia^3^. Estimates for the heritability of the quantitative trait refractive error have been reported to be as high as 90% (ref. 4). On the other hand, the rapid upsurge of myopia in the last few decades in many parts of the world is likely to be a consequence of lifestyle changes such as the increasing educational intensity, in particular in urban East Asia^5^,^6^, and potentially gene and environment (G × E) interactions.

Major attempts undertaken in genome-wide association studies (GWAS) to elucidate the genetic determination of myopia and refractive error have recently led to the discovery of >30 distinct susceptibility loci^7^,^8^. Nevertheless, collectively these genetic variants are estimated to explain <12% of phenotypic variance in refractive errror^7^,^8^. As myopia is a result of the combination of genetic and environmental factors, interplay between genes and environment may account for a substantial proportion of the phenotypic variance. In recent times, we showed interactions between education and genetic risk score of myopia derived from 26 known GWAS single-nucleotide polymorphisms (SNPs) in the Rotterdam Study^9^; the combined effect of genetic predisposition and education on the risk of myopia was substantially greater than anticipated from a simple sum of these two factors. At the gene level, some genes such as *SHISA6-DNAH9* have been shown to interact with education level and exhibit strong genetic effects for myopia among Asians with at least higher secondary education^10^. In the current study, we demonstrate that new genetic effects implicated in myopia development could be uncovered by studying interactions between genetic variants and education level.

In the context of the aetiology of refractive errors, education attainment is generally considered a surrogate measure for accumulated near work activity^1^. When viewing near objects, the eye generates extra optical power through the process of accommodation to focus the image on the retinal plane, to maintain clear vision^11^. There is an accommodative lag (less accommodation produced than needed) in many myopes, resulting in a hyperopic defocus on the retina for near work, which has long been proposed to promote eye growth^1^,^12^, but whether this occurs before or after the onset of myopia in humans is less clear. The retina has a central role in the mechanism linking such visual input with eye growth and refractive development^13^. Several neurotransmitters or molecules have been implicated in this process by animal studies including dopamine, acetylcholine, vasoactive intestinal peptide, GABA (γ-aminobutyric acid) and glucagon^14^,^15^. However, an organized framework for the retinal signalling mechanisms underlying refractive error development under various environmental conditions remains to be elucidated.

Factoring in environmental exposures may enhance power for the detection of genes, especially in circumstances where a genetic locus has a differential effect conditional on specific environment exposures^16^. Gene–environment-wide interaction studies (GEWIS) using a joint meta-analysis (JMA) approach on SNP main effects and SNP × environment interactions have recently been described^17^,^18^. This approach has successfully identified six novel loci associated with fasting insulin and glucose accounting for interactions with body mass index^18^. It also led to the identification of two novel loci for pulmonary function that did not emerge from analyses based on the genetic main effects alone^19^. The well-documented effects of educational attainment on myopia and refractive error make the proposed interaction an excellent analytical candidate for the GEWIS.

The availability of large-scale GWAS spherical equivalent data sets from the Consortium for Refractive Error And Myopia (CREAM) makes G × E interaction analyses feasible. To identify additional genetic variants for refractive error, we performed GEWIS-based analyses on 40,036 adults of European ancestry from 25 studies and 10,315 adults of Asian ancestry from 9 studies. We identified nine new loci using the JMA approach, where three loci exhibited G × E interaction on refractive error in Asians, including the GABA_C_ receptor subunit ρ1 gene *GABRR1*.

## Results

### Educational level and its main effects on spherical equivalent

**Figure 1:**
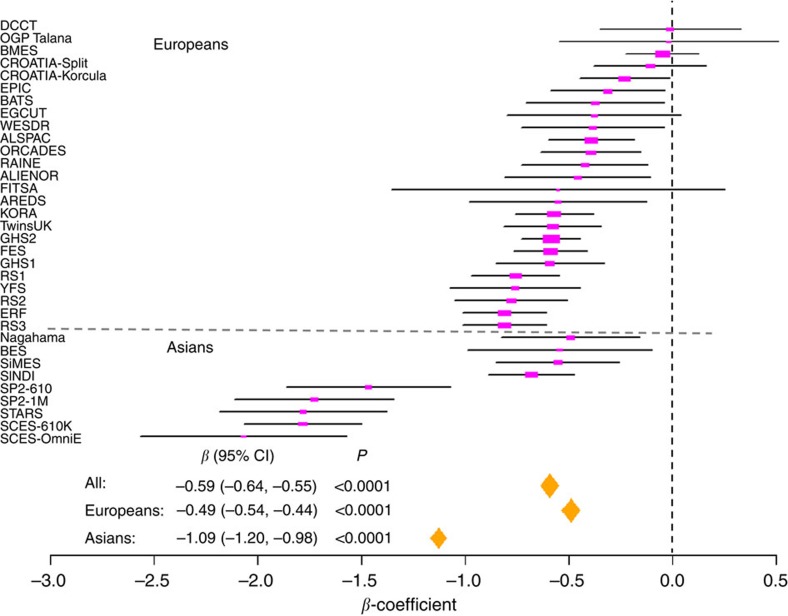
Forest plot of education main effects on spherical equivalent across studies. The *β*-coefficient represents the differences of dioptres in refractive error comparing individuals in higher education group versus lower education group in Europeans (*n*=40,036), Asians (*n*=10,315) and all studies (*n*=50,351). The studies are sorted by effect size of education on spherical equivalent within Europeans and Asians studies, respectively.

The baseline characteristics of 50,351 participants from 34 studies in our meta-analysis are shown in Table 1. A total of 40,036 participants were of European descent and 10,315 were of Asian descent; the age of the participants ranged from 20 to 99 years. We grouped individuals into two educational categories: a higher education group that included individuals who completed higher secondary or university education and a lower education group comprising those with lower secondary education or below (see Methods). Among Europeans, the proportions of participants in the higher education group ranged from 16.5% (FITSA^20^ and OGP Talana^21^) to 94.4% (AREDS^22^) with an average of 50.7% (Supplementary Table 1). In Asians, the proportions of individuals in the higher education group ranged from 6.7% (SiMES^23^) to 75.9% (Nagahama^24^) with an average of 30.0%. Across all studies, individuals in the higher education group had a spherical equivalent refractive error that was on average 0.59 dioptres (D) more myopic, or less hyperopic, compared with those in the lower education group (*β*=−0.59 D; 95% confidence interval (CI): −0.64 to −0.55 D). High education level was associated with a twofold more myopic spherical equivalent in individuals of Asian as compared with European ancestry (Asians: *β*=−1.09 D, 95% CI: −1.20 to −0.98 D; Europeans: *β*=−0.49 D, 95% CI: −0.54 to −0.44 D; Fig. 1). Among Asian studies, we also observed heterogeneity of education effects for refractive error. The education effects on spherical equivalent in Singapore Chinese were significantly larger than that in other Asian studies (Singapore Chinese: *β*=−1.75 D, 95% CI: −1.92 to −1.58 D; other Asian cohorts: *β*=−0.60 D, 95% CI: −0.75 to −0.46 D).

### GEWIS in Europeans

**Figure 2:**
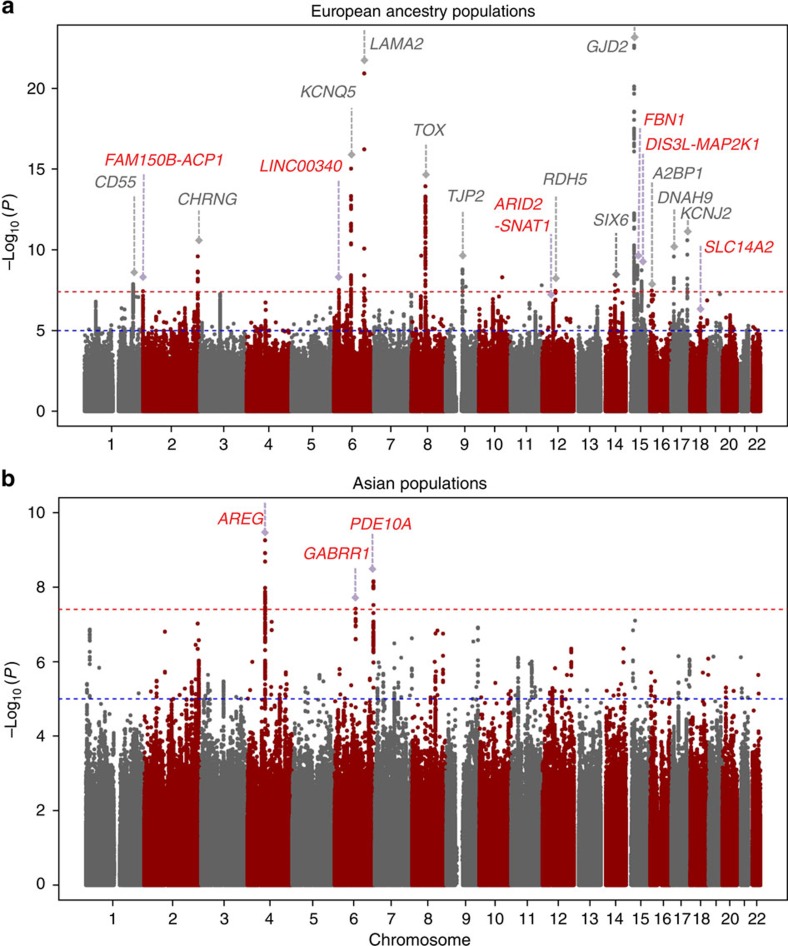
Manhattan plots of −log_10_(*P*) for the JMA on SNP main effects and SNP × education effects on spherical equivalent in (a) European ancestry populations and (b) Asian populations. The horizontal red line indicates the genome-wide significance level of *P*_JMA_<5 × 10^−8^. The horizontal blue line indicates the suggestive significance level of *P*_JMA_<1 × 10^−5^. Novel loci reaching genome-wide significance are labelled in red and known loci are in grey.

After stringent quality control (QC) filtering, ∼6 million SNPs in each study were eligible for the genome-wide JMA test (Supplementary Table 2). The JMA for SNP main effects and SNP × education interactions in 40,036 European Ancestry individuals showed an association with spherical equivalent at 12 previously implicated loci (Fig. 2a, Supplementary Table 3 and Supplementary Fig 1). We also identified four previously unreported loci associated with spherical equivalent achieving genome-wide significance (*P*_JMA_<5.0 × 10^−8^; *P*_het_≥0.086; Table 2): *FAM150B-ACP1*, *LINC00340*, *FBN1* and *DIS3L-MAP2K1*. The significant association for JMA testing at these loci in Europeans was primarily driven by SNP effects in both the lower and higher education strata (4.40 × 10^−8^≤*P*_main_≤1.35 × 10^−6^ and 7.61 × 10^−11^≤*P*_main_≤1.75 × 10^−6^, respectively). SNP × education interaction was not significant (*P*_int_≥0.208). The estimated effect sizes of SNP effects on spherical equivalent were highly similar across education strata.

### GEWIS in Asians

The JMA for spherical equivalent in 10,315 individuals from the Asians cohorts identified genome-wide significant association for three genes: *AREG*, *GABRR1* and *PDE10A* (*P*_JMA_<5.0 × 10^−8^; Table 3 and Fig. 2b). SNP × education interaction effects associated with spherical equivalent were observed at all three loci, with genetic effects significantly larger within participants who had a higher level of education compared with those with a lower education level: *AREG* (rs12511037, *β*_int_=−0.89±0.14 D, *P*_int_=6.87 × 10^−11^), *GABRR1* (rs13215566, *β*_int_=−0.56±0.14 D, *P*_int_=8.48 × 10^−5^) and *PDE10A* (rs12206610, *β*_int_=−0.72±0.13 D, *P*_int_=2.32 × 10^−8^). The genotype and phenotype associations were highly significant in the higher education stratum (main genetic effects, 1.97 × 10^−10^≤*P*_main_≤8.16 × 10^−8^) but were considerably weaker in the lower education stratum (0.008≤*P*_main_≤0.243). There was no evidence of inter-study heterogeneity at index SNPs within *AREG*, *GABRR1* or *PDE10A* loci (*Q*-test: *P*_het_≥0.122).

*GABRR1* and *PDE10A* index SNPs were not associated with spherical equivalent in European samples, for either the JMA test, SNP main effect or SNP × education interaction (Table 3). *AREG* SNP rs12511037 was excluded in the meta-analysis of European studies after QC filtering; hence, a proxy SNP, rs1246413, in linkage disequilibrium (LD) with rs12511037 in Asians (*r*^2^=0.97) was tested but not associated with spherical equivalent (*P*_JMA_=0.527; *P*_int_=0.176). The meta-regression including study-level characteristics as covariates in the model confirmed the heterogeneity between populations of European and Asian ancestry (*GABRR1*: *P*=0.006; *PDE10A*: *P*=0.0419; Supplementary Table 4). For *PDE10A*, besides ethnicity, average spherical equivalent of each study also explained the inter-study heterogeneity for the interaction effects (*P*=0.025).

We examined whether the underlying assumption of G × E independence held at these three G × E interaction loci. We performed a meta-analysis of logistic regression analysis for education level on *AREG* SNP rs12511037, *GABRR1* SNP rs13215566 and *PDE10A* SNP rs12296610, adjusting for age, gender and population stratification in Asian cohorts (*n*=10,315). Our analysis did not reveal any significant associations between these loci and education level (*P*≥0.102, *P*_het_≥0.170; Supplementary Table 5). Furthermore, the three loci were not associated with educational attainment in a large meta-analysis of GWAS recently conducted in European cohorts^25^. Thus, our G × E results are unlikely to be biased due to dependence between gene and education.

We also evaluated the association for spherical equivalent in Asian cohorts for four loci identified from European populations. Two of them showed significant associations in the JMA test: *FAM150B-ACP1* (*P*_JMA_=0.031) and *DIS3L-MAP2K1* (*P*_JMA_=0.0042; Table 2). The SNP effect sizes in lower and higher education strata in Asians were similar at *FAM150B-ACP1*. The signal at the *DIS3L-MAP2K1* locus was mainly driven by SNP × education interaction in Asians (*P*_int_=7.95 × 10^−4^), whereas the interaction effect was not statistically significant in Europeans (*P*_int_=0.208).

### Combined GEWIS of all cohorts

We subsequently conducted a JMA in the combined data including both the European and Asian participants of all 34 studies. This analysis revealed two additional SNPs: *ARID2-SNAT1* (*P*_JMA_=4.38 × 10^−8^) and *SLC14A2* (*P*_JMA_=2.54 × 10^−8^). Both loci showed suggestive association with spherical equivalent in European cohorts, with the same direction of effect and similar effect sizes in Asian cohorts (Table 2). We also detected genome-wide significant associations with spherical equivalent for 17 known loci^8^ identified in our previous CREAM GWAS (Supplementary Table 3). The regional plots of the identified novel loci are presented in Supplementary Fig 2.

### Gene and education interactions for GWAS known loci

We also evaluated the interactions between education and previously reported genetic association with spherical equivalent at 39 loci identified from recent two large GWAS studies^7^,^8^. Two SNP × education interactions were nominally significant (Supplementary Table 6): *TJP2* in Europeans (rs11145488, *P*_int_=6.91 × 10^−3^) and *SHISA6-DNAH9* in Asians (rs2969180, *P*_int_=4.02 × 10^−3^). In general, the index SNPs tested at 39 loci had larger SNP × education interaction effect on spherical equivalent in Asians versus Europeans (meta-regression *P* for fold changes<0.001; Supplementary Fig. 3). For 20 SNPs with the same direction of the interaction effect, the magnitudes of interaction effects were fourfold larger on average in Asians than in Europeans (*P*=0.003).

### Gene and near work interactions for three identified loci

High education levels may reflect an estimator for the greater accumulative effect of near work^26^,^27^. We thus examined whether there was evidence for SNP × near work interactions associated with spherical equivalent at the three loci (*AREG*, *GABRR1* and *PDE10A*) in paediatric cohorts (SCORM^28^, Guangzhou Twins^29^ and ALSPAC^30^; combined *n*=5,835; Supplementary Table 7). Tentative support for a SNP × near work interaction was observed for *PDE10A* (rs12206610, *P*_int_=0.032, *P*_het_=0.658), with the stronger genetic effect in children spending more hours on reading, writing or compute use. Weaker support for an interaction was noted at *GABRR1* (rs13215566, *P*_int_=0.309, *P*_het_=0.655), although the direction of meta-analysed interaction effect was largely consistent across paediatric studies with that observed in adults. We did not observe the interaction at *AREG* (rs12511037, *P*_int_=0.795, *P*_het_=0.062).

### Gene expression in human tissues

Using the Ocular Tissue Database^31^, we examined the expression of the associated genes in 20 normal human donor eyes. The majority of genes identified were expressed in human retina, sclera, choroid or retinal pigment epithelium (RPE) (Supplementary Table 8). Among these genes, *GABRR1*, *ACP1* and *SNAT1* had the highest expression in the retina. The Probe Logarithmic Intensity Error-normalized messenger RNA expression levels in the retina ranged from 121.66 to 236.69. Of note, *MAP2K1* was widely expressed in the retina, sclera and choroid/RPE.

## Discussion

This study represents the most comprehensive genome-wide scan of gene and education interactions to date, for refractive error. Here we identified novel genetic loci associated with refractive error by testing the joint contribution of SNP main effects and SNP × education effects in large multi-ethnic populations. Three loci (*AREG*, *GABRR1* and *PDE10A*) showed strong interactions with education in populations of Asian descent, with larger genetic effects within participants who had a higher level of education compared with those with a lower education level; no interactions achieved statistical significance in Europeans for top JMA associations or known myopic loci. Apart from confirming known associations at 17 previous published loci, we identified six new loci (*FAM150B-ACP1*, *LINC00340*, *FBN1*, *DIS3L-MAP2K1*, *ARID2-SNAT1* and *SLC14A2*) significantly associated with spherical equivalent using the combined multi-racial cohort.

A recent meta-analysis of GWAS in multi-ethnic populations comprises 32 studies (*n*=45,756) from CREAM and a large GWAS in Europeans (*n*=45,771) have reported a total of 39 genetic loci associated with refractive phenotypes^7^,^8^. The current genome-wide meta-analysis included ∼5,000 more subjects than the previous GWAS of main effects. We identified nine additional novel loci using the JMA approach. These loci can be placed within the biological context of the visually evoked signalling cascade that begins in the retina and mediates sclera remodelling^32^. The newly identified genes are involved in retinal neurotransmission (*GABRR1* and *SNAT1*), extracellular matrix remodelling (*FBN1*, *MAP2K1* and *AREG*), circadian rhythm (*PDE10A*) and platelet-derived growth factor receptor signalling (*ACP1*) (Supplementary Table 9). Network analysis revealed that most of the novel genes may tend to be co-expressed and co-localized with the known myopia susceptibility genes through multiple biological networks such as *LAMA2*, *GJD2*, *RASGRF1*, *BMP3*, *RDH5*, *ZMAT4*, *RBFOX1*, *RDH5* and so on (Supplementary Fig. 4). Our data, in line with previous findings, substantiate the assumption of heterogeneity in the molecular mechanisms involved in refractive error and myopia.

Among the novel loci, *GABRR1* on chromosome 6q15 (53 kb), encoding GABA_C_ receptor subunit ρ1, is an interesting functional candidate suggestive of a role in myopia development. Modulation of synaptic plasticity via GABA-mediated inhibition would be well placed to alter the ‘gain' of the visually guided feedback system controlling refractive development^33^. The lead SNP rs13215566 in *GABRR1*, together with seven SNPs within the LD block (*r*^2^≥0.8), are intronic, potentially affecting regulatory motifs (such as zfp128 and gcm1) that may influence transcriptional regulation (Supplementary Table 10). The variant rs13215029, in LD (*r*^2^=1) with rs13215566, is associated with *cis*-acting expression of *GABRR1* (*P*=2.3 × 10^−4^) in skin tissues (Supplementary Table 9)^34^. A recent pharmacological study provided evidence that retinal dopaminergic and GABAergic neurotransmitters play a substantial role in the modulation of refractive development in form-deprivation myopia^35^,^36^,^37^. Stone *et al*.^35^ have reported that antagonists to GABA-A, -B and -C receptors inhibited form-deprivation myopia in chicks, with greatest effect in the equatorial dimension. GABA receptors, expressed in bipolar and ganglion neuron cells, also interact with dopamine pathways in the retina^36^. A recent proteomics study determined that levels of GABA transporter-1 were significantly reduced in myopic murine retina after atropine treatment, implying that GABA signalling is involved in the anti-myopic effects of atropine^37^. Altogether, these data suggest that GABA_C_ receptor ρ1 may regulate the development of myopia through functional feedback from RPE to neuron cells in the retina. Further studies are needed to investigate the effect of genetic deletion of *GABRR1* on refractive eye development and the role of the GABAergic pathway in myopia development using gene knockout mice. Therefore, our result in humans is in line with animal experiments, supporting the notion that the GABAergic neurotransmitter signalling pathway in the retina could be a potential factor in the progression of myopia.

SNP rs10889855 on chromosome 6 is an intronic variant within the *ARID2* gene (AT-rich interactive domain 2) and 500 kb downstream of *SNAT1* (solute carrier family 38, member, alias *SLC38A1*). SNAT1 is a transporter of glutamine, a precursor of GABA^38^. It is also highly expressed in human retina. In our previous meta-analysis in CREAM^8^, we identified variants in another glutamate receptor gene *GRIA4* (encoding glutamate receptor, ionotropic); altogether, current evidence supports the notion that retinal neurotransmitters GABA and glutamine may be involved in the refractive development.

The strongest association signal for gene and environment interactions was from rs12511037, located 14 kb downstream the *AREG* gene (amphiregulin). AREG is a ligand of the epidermal growth factor receptor promoting the growth of normal epithelial cells, which is critical for cell differentiation and proliferation such as regrowth of the wounded cornea^39^. A link has been found between the muscarinic acetylcholine receptors, dominant in myopia progression, and the epidermal growth factor receptor in muscarinic system^40^,^41^.

Another novel association, rs16949788 on chromosome 15, derives from a region that spans *DIS3L* and *MAP2K1*. *MAP2K1* encodes mitogen-activated protein kinase 1, which binds to muscarinic receptors during proliferation^42^ and inhibits the proliferation of human scleral fibroblasts exposed to all-*trans* retinoic acid^43^. All-*trans* retinoic acid is a modulator of ocular growth, inhibiting the proliferation of human scleral fibroblasts^44^.

*FBN1* (Fibrillin 1), a member of the fibrillin family, encodes a large extracellular matrix glycoprotein. Mutations in *FBN1* cause Marfan's syndrome, a disorder of connective tissue affecting the ocular, skeletal and cardiovascular systems^45^. As a candidate gene for myopia, attempts to study its association with myopia previously produced inconclusive results^46^,^47^, probably owing, in part, to underpowered studies with insufficient sample sizes. Using data from a large multi-ethnic population, our results support the role of *FBN1* in myopia development.

The genome-wide significant SNPs from the JMA approach did not exhibit any interactions with education in Europeans, in contrast to the significant interactive effect among Asians. In particular, the G × E interactions at *AREG*, *GABRR1* and *PDE10A* were only evident in Asian populations. There are a number of possible reasons for the observed differences. First, the variation of LD patterns and joint effects of genetic variants might affect the transferability of G × E signals across populations. Similar LD patterns were seen at *GABRR1* and *PDE10A* regions across populations whereas a long stretch of LD flanking AREG was present only in Asian populations (Supplementary Fig. 5). As the true causal variants transferrable across populations are probably not implicated in our study, the identified novel myopia risk loci provide a much-needed starting point for follow-up and functional downstream analyses. Second, we used education level in adults as a surrogate measure of the underlying risk factors for influencing refractive development. Ideally, near work intensity would be measured prospectively in children before the onset of myopia. Studies included in our analyses do not have additional data on relevant childhood exposures. Thus, the best available surrogate measure of cumulative near work exposure in adult cohorts is educational level. We nevertheless believe that education is a highly reliable proxy for the relevant exposures underlying refractive development and is universally associated with refractive error in our study. Third, the differences of G × E interactions in Asian versus Europeans may reflect quantitative differences in near work intensity during childhood. For example, 6- and 7- year-old children in England and Australia reported less near work activity outside of school (1.0–2.3 h per day)^48^,^49^ compared with children in Singapore and China (2.7–3.5 h per day)^50^,^51^. A similar trend was observed in older children^48^,^52^,^53^,^54^,^55^. We thus speculate that the total exposure to near work activity may be greater in East Asians compared with European-derived populations with the same levels of education; hence, G × E interaction estimates would tend to be inflated in Asian populations compared with European groups. Fourth, other environmental factors such as outdoor activities could also interact with genes. East Asian children tend to have less exposure to outdoor activities compared with their European peers^56^. However, the majority of adult cohorts did not report time outdoors and thus could not be accounted for in the current study. Finally, the population mean of refractive error is less myopic in Europeans (0.10 D) versus Asians (−0.60 D). Of note, for the previously known myopia loci, the magnitudes of interaction effects were fourfold larger on average in Asians than in Europeans (Supplementary Fig. 2). The impact of G × E interactions may be seen at certain severity levels of myopia.

The risk alleles of rs12511037 in *AREG*, rs1321556 in *GABRR1* and rs12206610 in *PDE10A* had no or weak influence on myopic shift in the lower education group compared with the higher education group. This suggests that the hereditary predisposition to myopia could be latent for the risk allele carriers, if they are less exposed to the myopiagenic environment associated with high-level education. A lack of strong SNP × near work associations in children might be due to the inadequate statistical power in paediatric cohorts of relatively small sample sizes, or the possibility that environmental risk exposures other than near work might underlie the SNP × education interaction seen in the adult Asian samples.

In summary, we identified nine novel loci associated with refractive error in a large multi-ethnic cohort study by GEWIS approach. Our data provide evidence that specific genetic variants interact with education, to influence refractive development, and further support a role for GABA neurotransmitter signalling in myopia development. These findings provide promising candidate genes for follow-up work and may lead to new genetic targets for therapeutic interventions on myopia.

## Methods

### Study participants

Thirty-four studies from members of CREAM, comprising 40,036 individuals of European ancestry from 25 studies and 10,315 individuals of Asian ancestry from 9 studies, were made available for this analysis (Table 1 and Supplementary Note 1). Individuals aged <20 years were excluded and so were those who had undergone cataract surgery, laser or other intra-ocular procedures that could alter refraction. Many of these studies were also included in the previous CREAM GWAS on spherical equivalent^8^. All studies adhered to the tenets of the Declaration of Helsinki and were approved by their local research ethics committees. The exact names of the Institutional Research Board committees can be found under Supplementary Note 2. All participants provided a signed consent form before the start of the study.

### Phenotyping and education levels

All participants underwent ophthalmological examinations (Supplementary Table 1). Non-cycloplegic refraction was measured by autorefraction and/or subjective refraction. Spherical equivalent was calculated as the sphere power plus half of the cylinder power for each eye. The mean spherical equivalent of the right and left eyes was used as a quantitative outcome. When data from only one eye was available, the spherical equivalent of that eye was used. For each study, the participants reported the highest level of education achieved or the years of schooling through a self-reported questionnaire, or in an interview.

We dichotomized education for all participants in each study. The higher education group consisted of those who had achieved the highest educational level of A-levels, high school (higher secondary education), vocational training (for example, diploma), university degree or those with ≥12 years spent in formal education (beginning from first grade). Those who had achieved the highest educational level of O-level, middle school (lower secondary education) or those with <12 years of formal education were classified into the lower education group. If both number of formal study years and education levels were available in the cohort, we classified participants based on years of formal education. For the four cohorts of relatively young European participants (BATS, DCCT, RAINE and WESDR; total *n*=2,349), almost all of them had completed 12 or more years of schooling. We thus chose to categorize individuals with tertiary or university education as the higher education group in these studies. Sensitivity analysis excluding these four cohorts did not appreciably change our meta-analysis results.

### Genotyping and imputation

Detailed information on the genotyping platforms and QC procedures for each study is provided in Supplementary Table 2 and Supplementary Note 1. Each study applied stringent QC filters for GWAS. In general, duplicate DNA samples, individuals with low call rate (<95%), gender mismatch or ethnic outliers were excluded. SNPs were excluded if low genotyping call rate (>5% missingness), monomorphic SNPs, with minor allele frequency (MAF) <1% or in Hardy–Weinberg disequilibrium (*P*<10^−6^). After QC filtering, the array genotypes of each study were imputed using the 1000 Genomes Project data as reference panels (build 37, phase 1 release, March 2012) with the software Minimac^57^ or IMPUTE2 (ref. 58). Approximately six million SNPs that passed imputation quality thresholds (MACH: *r*^2^>0.5 or IMPUTE info score >0.5) and with MAF ≥5% were eligible for the meta-analysis (Supplementary Table 2).

### Statistical models

For each study, a linear regression model for each genotyped or imputed SNP was constructed with the mean spherical equivalent as the outcome. We assumed an additive genetic model where the number of risk alleles is an ordinal variable (0, 1 and 2) for directly genotyped SNPs or a continuous variable of allele dosage probability ranging from 0 to 2 for imputed SNPs. The primary analytic model included SNP, education and SNP × education interaction term, as well as age and sex as covariates. Additional adjustments for the top principal components of genomic marker variations were performed in individual studies when applicable (that is, when there was evidence of population stratification).

We used the following additive genetic model to test for a joint effect of SNP (*β*_SNP_) and SNP × education interaction (*β*_SNP × education_) on mean spherical equivalent:


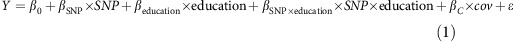


where *Y* is the mean spherical equivalent and education is a dichotomous variable (0=lower education group and 1=higher education group); *cov* is a set of covariates such as age, sex and first top five principal components when applicable. For family-based studies, the kinship matrix was estimated empirically from the SNP data and included as a random effect in the generalized mixed model^59^. To test an effect of SNP × education interaction, we assessed *β*_SNP × education_ from equation (1).

The linear regression analyses in each study were conducted with Quickes or ProbABEL for the unrelated samples and MixABEL for family-based data (see URLs). The command ‘robust' was used in the above software to calculate the robust (‘sandwich', Huber-White) s.e. of *β*_SNP_ and *β*_SNP × education_, and error covariance of *β*, to correct the potential inflation of false positive rate for the interaction *P*-value^60^.

In addition, each study also tested the main effect of education on spherical equivalent by adjusting for age and gender using the linear regression model:


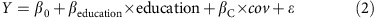


where the definition of each variable is the same as in equation (1). We performed meta-analysis of the education effects on mean spherical equivalent in Europeans, Asians (Singapore Chinese versus others) and combined data using a fixed-effect model with inverse-variance weighting (R package ‘meta').

### GEWIS join meta-analyses

We adopted the JMA approach^17^,^61^, to simultaneously test both SNP main effects and SNP × education interactions for spherical equivalent with a fixed-effect model, using SNP and SNP × education regression coefficients (*β*_SNP_ and *β*_SNP × education_, respectively) and a *β*'s covariance matrix from each study. A Wald's statistic, following a *χ*^2^-distribution with two degrees of freedom, was used to test the joint significance of the *β*_SNP_ and *β*_SNP × education_. The JMA was performed with METAL^62^, as previously described by Manning *et al*.^61^. A Cochran's *Q*-test was used to assess heterogeneity of the *β*-coefficients across studies for the SNP and interaction effects. To test for interaction between the SNP and education, we conducted a secondary meta-analysis of the SNP × education interaction effects for spherical equivalent (*β*_SNP × education_, one degree of freedom), with a fixed-effects model using inverse-variance weighting in METAL; this is a traditional meta-analysis to investigate SNP × education interactions *per se*. Effects and s.e. of the SNP effect on spherical equivalent in the lower education group (*β*_SNP_) and higher education group (*β*_SNP_+*β*_SNP × education_) were derived from the JMA output^61^. We used the *P*-value of 5 × 10^−8^ as a significant threshold for JMA test. For the SNP and SNP × education effects for the identified top loci, the *P*-value threshold for significance was set at 0.0055=0.05/9 (9 index SNPs underlying analyses).

We performed a meta-regression to explore sources of heterogeneity in our meta-analysis for three loci showing G × E interactions (R package ‘metafor'). Meta-regression included the following study-specific variables as covariates: study sample size, proportion of individuals in the higher education group, average spherical equivalent, education main effects on spherical equivalent (higher education level versus lower), ethnicity (Asian versus European), study design (independent samples versus family-based studies), study year and average age. Meta-regression was also conducted to test the fold changes of the interaction *β*-coefficients in Asians versus Europeans for the 39 known myopia loci.

The study-specific genomic control inflation factors *λ*_gc_ for the joint test for SNP and interaction terms ranged from 1.009 to 1.125 with an average of 1.019 (Supplementary Table 2), calculated by the ratio of the observed median *χ*^2^ divided by the expected median of the 2df *χ*^2^-distribution (1.382). Genomic control correction was applied to each individual study^63^. For studies of small sample sizes (*n*<500) with *λ*_gc_ >1.05, we further, before starting the meta-analysis, excluded SNPs showing significant joint *P*-value <1 × 10^−5^ but neither the SNP nor SNP × education effects supported such an association. Quantile–quantile plots showed only modest inflation of the test statistics in the JMA test (Europeans: *λ*_gc_=1.081; Asians: *λ*_gc_=1.053; Combined: *λ*_gc_=1.092; Supplementary Fig. 1), similar to previous GEWIS studies with comparable sample sizes^18^,^19^. We excluded a small number of markers in the meta-analysis with *P*_het_<0.0001. The *λ*_gc_ for the SNP × education interaction term in the individual studies ranged from 1.01 to 1.08, indicating little evidence of test statistic inflation on SNP × education effect for each study.

### Annotation of genetic variants and gene expression in humans

The coordinates and variant identifiers are reported on the NCBI B37 (hg19) genome build and annotated using UCSC Genome Browser^64^. We identified variants within each of the LD blocks (*r*^2^≥0.8) in European and Asian populations of the 1000 Genomes Project (100 kb flanking the top SNP at each locus), to apply functional annotations of transcription regulation using HaploReg^65^ and Encyclopedia of DNA Elements^66^ data. We also generated funcational association and co-expression network using GeneMANIA^67^, to determine whether the disease-related genes identified in this study and previous GWAS^7^,^8^ are functionally connected.

To assess gene expression in human tissues, we examined the Ocular Tissue Database and the EyeSAGE database^31^,^68^. The estimated gene and exome-level abundances are available online (see URL). Normalization of gene expression used the Probe Logarithmic Intensity Error method with genomic control-background correction^31^. Relationships between genotype and *cis* regulation of gene expression levels (Supplementary Table 9) were assessed using expression quantitative trait locus associations in multiple human tissues from UK samples^34^, as well as gene expression profiles obtained from GTExPortal database^69^.

## Additional information

**How to cite this article:** Fan, Q. *et al*. Meta-analysis of gene–environment-wide association scans accounting for education level identifies additional loci for refractive error. *Nat. Commun.* 7:11008 doi: 10.1038/ncomms11008 (2016).

## Supplementary Material

Supplementary Information Supplementary Figures 1-5, Supplementary Tables 1-10, Supplementary Notes 1-2 and Supplementary References

## Figures and Tables

**Table 1:** Characteristics of study participants.

Study	*N*	Study year	Age (s.d.)	Age range	Male (%)	Spherical equivalent
*Europeans (*n=*40,036)*						
ALIENOR	509	2006–2008	79.2 (4.1)	73–93	43.2	0.98 (1.98)
ALSPAC	1,865	1999–2000	45.9 (4.5)	32–59	0	−0.76 (2.16)
AREDS	1,842	1992	68.1 (4.7)	55–81	41.0	0.54 (2.15)
BATS	383	1992–2013	24.8 (7.8)	20–67	41.3	−0.67 (1.58)
BMES	1,896	1992–2009	66.8 (8.9)	49–94	43.8	0.58 (1.94)
CROATIA-Korcula	807	2007–2008	56.2 (13.3)	25–94	34.9	−0.13 (1.59)
CROATIA-Split	787	2008–2009	51.9 (13.0)	25–80	38.6	−1.27 (1.59)
DCCT	1,057	1982–1993	35.4 (5.8)	25–49	54.1	−1.47 (1.80)
EGCUT	904	2002–2013	56 (17.0)	25–99	38.8	0.33 (3.36)
EPIC	1,083	2004–2011	68.8 (7.5)	50–88	43.8	0.34 (2.27)
ERF	2,604	2002–2005	48.9 (14.4)	25–87	45.0	0.12 (2.03)
FES	2,479	1973–1975/ 1989–1991	54.8 (9.3)	28–84	55.3	0.27 (2.37)
FITSA	188	2000–2001	68.5 (3.3)	63–76	0	1.44 (2.08)
GHS1	3,178	2007–2008	55.3 (10.9)	35–74	50.4	−0.38 (2.47)
GHS2	1,354	2008	54.6 (10.8)	36–74	49.6	−0.39 (2.51)
KORA	2326	2004–2006	55.1 (11.8)	35–84	49.4	−0.26 (2.18)
OGP Talana	456	2002	52.6 (16.3)	25–89	57.3	−0.20 (0.24)
ORCADES	1,124	2009	56.5 (13.2)	29–92	39.1	0.10 (2.07)
RAINE	348	2010–2012	20.4 (0.34)	20–22	49.1	0.03 (1.29)
RS1	5,702	1991–1993	68.7 (8.7)	55–99	41.0	0.83 (2.55)
RS2	2,021	2000–2002	64.3 (7.9)	55–95	46.0	0.48 (2.51)
RS3	2,918	2006–2009	56.9 (6.6)	45–86	44.0	−0.28 (2.60)
TwinsUK	2,154	1998–2010	53.8 (11.4)	25–84	8.4	−0.96 (2.78)
WESDR	561	1979–2007	31.7 (7.0)	25–65	50.3	−1.65 (2.07)
** **YFS	1,490	2011	41.9 (5.0)	34–49	44.6	−1.09 (2.16)
						
*Asians (*n=*10,315)*						
BES	589	2006–2011	62.1 (8.5)	50–90	34.0	−0.06 (1.86)
Nagahama	723	2008–2010	49.2 (15.2)	30–74	33.6	−1.93 (2.46)
SCES-610K	1,710	2009–2011	57.5 (7.0)	44–84	51.6	−0.72 (2.69)
SCES-OmniE	543	2011–2012	59.3 (8.9)	46–83	51.2	−0.89 (2.74)
SiMES	2,256	2004–2006	46.8 (10.2)	40–80	49.1	−0.03 (1.81)
SINDI	2,088	2007–2009	55.8 (8.8)	43–84	51.5	0.04 (2.07)
SP2-1M	811	1992–1998	46.8 (10.2)	25–80	62.3	−1.80 (2.84)
SP2-610	854	1992–1998	48.4(11.3)	25–82	19.6	−1.44 (2.89)
STARS	741	2007–2009	38.5 (5.2)	26–58	52.4	−2.80 (2.85)

ALIENOR, antioxydants, lipids essentiels, nutrition et maladies oculaiRes; ALSPAC, avon longitudinal study of parents and children; AREDS, age-related eye disease study; BATS, Brisbane adolescent twins study; BMES, blue mountains eye study; DCCT, diabetes control and complications trial; EGCUT, estonian genome center of the university of Tartu; EPIC, EPIC-Norfolk eye study; ERF, erasmus rucphen family study; FES, Framingham eye study; FITSA, finnish twin study on aging; GHS, Gutenberg health study; KORA, cooperative health research in the region of Augsburg; OGP Talana, ogliastra genetic park, talana study; ORCADES, orkney complex disease study; RAINE, RAINE eye health study; RS, Rotterdam study; TwinsUK, Twins UK study; WESDR, Wisconsin epidemiologic study of diabetic retinopathy; YFS, young finns study; BES, Beijing eye study; SCES, Singapore Chinese eye study; SiMES, Singapore Malay eye study; SINDI, Singapore Indian eye study; SP2, Singapore prospective study program; STARS, strabismus, amblyopia and refractive error study in preschool singaporean children. s.d., standard deviation; age in years; spherical equivalent in dioptres.

Details of each study cohort are described in Supplementary Note 1.

**Table 2:** Six genetic loci associated with spherical equivalent from the JMA in the European populations and combined analyses.

SNP (Chr:BP)	Gene	Allele	FREQ	Subgroup	Europeans (*n*=40,036)			Asians (*n*=10,315)			All (*n*=50,351)		
					*β*	*P*-value	*P*_het_	*β*	*P*-value	*P*_het_	*β*	*P*-value	*P*_het_
rs60843830 (2:286756)	*FAM150B-ACP1*	C/G	0.66/0.74	JMA		3.71 × 10^−8^	0.086		0.031	0.980		1.27 × 10^−9^	0.395
				Lower education	−0.11	4.73 × 10^−8^		−0.09	0.010		−0.10	1.65 × 10^−9^	
				Higher education	−0.09	1.75 × 10^−6^		−0.06	0.509		−0.09	9.83 × 10^−7^	
rs10946507 (6:22100367)	*LINC00340 (6p22.3)*	A/G	0.47/0.16	JMA		3.07 × 10^−8^	0.213		0.433	0.396		2.24 × 10^−8^	0.249
				Lower education	−0.08	7.08 × 10^−7^		−0.04	0.313		−0.08	6.13 × 10^−7^	
				Higher education	−0.09	1.19 × 10^−8^		−0.08	0.450		−0.09	1.20 × 10^−8^	
rs8023401 (15:48703823)	*FBN1*	G/A	0.87/0.95	JMA		1.66 × 10^−9^	0.180		0.572	0.979		2.85 × 10^−9^	0.495
				Lower education	−0.15	4.40 × 10^−8^		−0.06	0.304		−0.13	8.17 × 10^−8^	
				Higher education	−0.16	7.61 × 10^−11^		−0.03	0.828		−0.14	2.02 × 10^−9^	
rs16949788 (15:66590037)	*DIS3L-MAP2K1*	T/C	0.91/0.94	JMA		1.34 × 10^−8^	0.721		0.0042	0.219		2.19 × 10^−8^	0.245
				Lower education	−0.15	1.35 × 10^−6^		0.21	0.103		−0.13	4.88 × 10^−6^	
				Higher education	−0.17	1.89 × 10^−9^		−0.59	0.014		−0.16	3.90 × 10^−9^	
rs10880855 (12:46144855)	*ARID2-SNAT1*	T/C	0.51/0.43	JMA		7.83 × 10^−7^	0.790		0.019	0.779		4.38 × 10^−8^	0.867
				Lower education	−0.09	1.26 × 10^−7^		−0.06	0.067		−0.09	8.42 × 10^−9^	
				Higher education	−0.07	1.60 × 10^−5^		−0.16	0.033		−0.07	3.55 × 10^−6^	
rs10853531 (18:42824449)	*SLC14A2*	G/A	0.80/0.83	JMA		7.82 × 10^−6^	0.052		0.0023	0.812		2.54 × 10^−8^	0.111
				Lower education	−0.11	1.27 × 10^−6^		−0.15	9.01 × 10^−4^		−0.11	3.38 × 10^−9^	
				Higher education	−0.08	2.12 × 10^−6^		−0.11	0.288		−0.09	7.14 × 10^−6^	

*β*, *β*-coefficient corresponds to the effect in spherical equivalent (dioptres) for 1 additional copy of the risk allele in the higher or lower education group. FREQ, allele frequency of the risk allele in European/Asian cohorts; JMA, joint meta-analysis on SNP effect and SNP × education interaction effect on spherical equivalent; *P*_het_, *P*-value for the test of heterogeneity at each SNP; SNP, single-nucleotide polymorphism. Allele, risk allele/other allele.

**Table 3:** Three genetic loci associated with spherical equivalent with a significant SNP × education interaction in Asians and results in European populations.

SNP (Chr:BP)	Gene	Allele	FREQ	Subgroup		Asians (*n*=10,315)			Europeans (*n*=40,036)	
					*β*	*P*-value	*P*_het_	*β*	*P*-value	*P*_het_
rs12511037* (4:75334864)	*AREG*	C/T	0.91/0.95	Lower education	0.07	0.243		−0.05	0.323	
				Higher education	−0.70	1.97 × 10^−10^		−0.03	0.579	
				SNP × education	−0.89	6.87 × 10^−11^	0.704	0.02	0.176	0.284
				JMA		5.55 × 10^−10^	0.405		0.527	0.186
rs13215566 (6:89918638)	*GABRR1*	C/G	0.94/0.84	Lower education	−0.13	0.030		−0.03	0.258	
				Higher education	−0.68	1.46 × 10^−8^		−0.01	0.817	
				SNP × education	−0.56	8.48 × 10^−5^	0.134	−0.02	0.459	0.457
				JMA		3.81 × 10^−8^	0.122		0.502	0.630
rs12206610 6:166016800	*PDE10A*	C/T	0.90/0.87	Lower education	0.16	0.008		0.01	0.759	
				Higher education	−0.59	8.16 × 10^−8^		0.01	0.810	
				SNP × education	−0.72	2.32 × 10^−8^	0.920	−0.002	0.421	0.111
				JMA		9.21 × 10^−9^	0.902		0.954	0.305

*β* (higher education/lower education), *β*-coefficient corresponds to the effect in spherical equivalent (dioptres) for 1 additional copy of the effect allele in the higher/lower education group; *β* (SNP × education), *β*-coefficient corresponds to the difference in spherical equivalent (dioptres) for 1 additional copy of the effect allele in the higher versus lower education group; FREQ, allele frequency of the effect allele in Asian/European cohorts; JMA, joint meta-analysis on SNP effect and SNP × education interaction effect on spherical equivalent; LD, linkage disequilibrium; *P*_het_, *P*-value for the test of heterogeneity; SNP, single-nucleotide polymorphism.

*β* and *P*-values for SNP × education interaction were calculated by the meta-analysis of conducting a 1df Wald's test of single interaction parameter. Allele is listed as effect allele/other allele.

^*^SNP rs12511037 was not present in European studies after quality control. Here we present the results of a proxy SNP rs1246413 (*T*/*G*, frequency of risk allele *T*=0.95) in LD with rs12511037 (*r*^2^=0.97).

## References

[b1] Morgan I. G., Ohno-Matsui K. & Saw S. M. Myopia. Lancet 379, 1739–1748 (2012). 22559900 10.1016/S0140-6736(12)60272-4

[b2] Saw S. M., Gazzard G., Shih-Yen E. C. & Chua W. H. Myopia and associated pathological complications. Ophthalmic Physiol. Opt. 25, 381–391 (2005). 16101943 10.1111/j.1475-1313.2005.00298.x

[b3] Wojciechowski R. Nature and nurture: the complex genetics of myopia and refractive error. Clin. Genet. 79, 301–320 (2011). 21155761 10.1111/j.1399-0004.2010.01592.x PMC3058260

[b4] Sanfilippo P. G., Hewitt A. W., Hammond C. J. & Mackey D. A. The heritability of ocular traits. Surv. Ophthalmol. 55, 561–583 (2010). 20851442 10.1016/j.survophthal.2010.07.003

[b5] Wong T. Y. . Prevalence and risk factors for refractive errors in adult Chinese in Singapore. Invest. Ophthalmol. Vis. Sci. 41, 2486–2494 (2000). 10937558

[b6] Kempen J. H. . The prevalence of refractive errors among adults in the United States, Western Europe, and Australia. Arch. Ophthalmol. 122, 495–505 (2004). 15078666 10.1001/archopht.122.4.495

[b7] Kiefer A. K. . Genome-wide analysis points to roles for extracellular matrix remodeling, the visual cycle, and neuronal development in myopia. PLoS Genet. 9, e1003299 (2013). 23468642 10.1371/journal.pgen.1003299 PMC3585144

[b8] Verhoeven V. J. . Genome-wide meta-analyses of multiancestry cohorts identify multiple new susceptibility loci for refractive error and myopia. Nat. Genet. 45, 314–318 (2013). 23396134 10.1038/ng.2554 PMC3740568

[b9] Verhoeven V. J. . Education influences the role of genetics in myopia. Eur. J. Epidemiol. 28, 973–980 (2013). 24142238 10.1007/s10654-013-9856-1 PMC3898347

[b10] Fan Q. . Education influences the association between genetic variants and refractive error: a meta-analysis of five Singapore studies. Hum. Mol. Genet. 23, 546–554 (2014). 24014484 10.1093/hmg/ddt431 PMC3869359

[b11] Goss D. A. Nearwork and myopia. Lancet 356, 1456–1457 (2000). 11081523 10.1016/S0140-6736(00)02864-6

[b12] Gwiazda J., Thorn F. & Held R. Accommodation, accommodative convergence, and response AC/A ratios before and at the onset of myopia in children. Optom. Vis. Sci. 82, 273–278 (2005). 15829855 10.1097/01.opx.0000159363.07082.7d

[b13] Strauss O. The retinal pigment epithelium in visual function. Physiol. Rev. 85, 845–881 (2005). 15987797 10.1152/physrev.00021.2004

[b14] Rymer J. & Wildsoet C. F. The role of the retinal pigment epithelium in eye growth regulation and myopia: a review. Vis. Neurosci. 22, 251–261 (2005). 16079001 10.1017/S0952523805223015

[b15] Cottriall C. L., Brew J., Vessey K. A. & McBrien N. A. Diisopropylfluorophosphate alters retinal neurotransmitter levels and reduces experimentally-induced myopia. Naunyn Schmiedebergs Arch. Pharmacol. 364, 372–382 (2001). 11683525 10.1007/s002100100460

[b16] Thomas D. Gene--environment-wide association studies: emerging approaches. Nat. Rev. Genet. 11, 259–272 (2010). 20212493 10.1038/nrg2764 PMC2891422

[b17] Aschard H., Hancock D. B., London S. J. & Kraft P. Genome-wide meta-analysis of joint tests for genetic and gene-environment interaction effects. Hum. Hered. 70, 292–300 (2010). 21293137 10.1159/000323318 PMC3085519

[b18] Manning A. K. . A genome-wide approach accounting for body mass index identifies genetic variants influencing fasting glycemic traits and insulin resistance. Nat. Genet. 44, 659–669 (2012). 22581228 10.1038/ng.2274 PMC3613127

[b19] Hancock D. B. . Genome-wide joint meta-analysis of SNP and SNP-by-smoking interaction identifies novel loci for pulmonary function. PLoS Genet. 8, e1003098 (2012). 23284291 10.1371/journal.pgen.1003098 PMC3527213

[b20] Parssinen O. . Heritability of spherical equivalent: a population-based twin study among 63- to 76-year-old female twins. Ophthalmology 117, 1908–1911 (2010). 20630598 10.1016/j.ophtha.2010.02.008

[b21] Biino G. . Ocular refraction: heritability and genome-wide search for eye morphometry traits in an isolated Sardinian population. Hum. Genet. 116, 152–159 (2005). 15611866 10.1007/s00439-004-1231-6

[b22] Age-Related Eye Disease Study Research, G. A randomized, placebo-controlled, clinical trial of high-dose supplementation with vitamins C and E, beta carotene, and zinc for age-related macular degeneration and vision loss: AREDS report no. 8. Arch. Ophthalmol. 119, 1417–1436 (2001). 11594942 10.1001/archopht.119.10.1417 PMC1462955

[b23] Foong A. W. . Rationale and methodology for a population-based study of eye diseases in Malay people: The Singapore Malay eye study (SiMES). Ophthalmic Epidemiol. 14, 25–35 (2007). 17365815 10.1080/09286580600878844

[b24] Nakata I. . Prevalence and characteristics of age-related macular degeneration in the Japanese population: the Nagahama study. Am. J. Ophthalmol. 156, 1002–1009 e2 (2013). 23938127 10.1016/j.ajo.2013.06.007

[b25] Rietveld C. A. . GWAS of 126,559 individuals identifies genetic variants associated with educational attainment. Science 340, 1467–1471 (2013). 23722424 10.1126/science.1235488 PMC3751588

[b26] Lee Y. Y., Lo C. T., Sheu S. J. & Lin J. L. What factors are associated with myopia in young adults? A survey study in Taiwan Military Conscripts. Invest. Ophthalmol. Vis. Sci. 54, 1026–1033 (2013). 23322575 10.1167/iovs.12-10480

[b27] Wong L., Coggon D., Cruddas M. & Hwang C. H. Education, reading, and familial tendency as risk factors for myopia in Hong Kong fishermen. J. Epidemiol. Community Health 47, 50–53 (1993). 8436895 10.1136/jech.47.1.50 PMC1059711

[b28] Saw S. M., Hong C. Y., Chia K. S., Stone R. A. & Tan D. Nearwork and myopia in young children. Lancet 357, 390 (2001). 11211020 10.1016/S0140-6736(05)71520-8

[b29] Zheng Y., Ding X., Chen Y. & He M. The Guangzhou Twin Project: an update. Twin Res. Hum. Genet. 16, 73–78 (2013). 23186635 10.1017/thg.2012.120

[b30] Fraser A. . Cohort Profile: the Avon Longitudinal Study of Parents and Children: ALSPAC mothers cohort. Int. J. Epidemiol. 42, 97–110 (2013). 22507742 10.1093/ije/dys066 PMC3600619

[b31] Wagner A. H. . Exon-level expression profiling of ocular tissues. Exp. Eye Res. 111, 105–111 (2013). 23500522 10.1016/j.exer.2013.03.004 PMC3664108

[b32] Young T. L., Metlapally R. & Shay A. E. Complex trait genetics of refractive error. Arch. Ophthalmol. 125, 38–48 (2007). 17210850 10.1001/archopht.125.1.38

[b33] Chen Y. P. . Selective breeding for susceptibility to myopia reveals a gene-environment interaction. Invest. Ophthalmol. Vis. Sci. 52, 4003–4011 (2011). 21436268 10.1167/iovs.10-7044

[b34] Grundberg E. . Mapping cis- and trans-regulatory effects across multiple tissues in twins. Nat. Genet. 44, 1084–1089 (2012). 22941192 10.1038/ng.2394 PMC3784328

[b35] Stone R. A. . GABA, experimental myopia, and ocular growth in chick. Invest. Ophthalmol. Vis. Sci. 44, 3933–3946 (2003). 12939312 10.1167/iovs.02-0774

[b36] Schmid K. L., Strasberg G., Rayner C. L. & Hartfield P. J. The effects and interactions of GABAergic and dopaminergic agents in the prevention of form deprivation myopia by brief periods of normal vision. Exp. Eye Res. 110, 88–95 (2013). 23474145 10.1016/j.exer.2013.02.017

[b37] Barathi V. A. . Involvement of GABA transporters in atropine-treated myopic retina as revealed by iTRAQ quantitative proteomics. J. Proteome Res. (2014). 10.1021/pr500558y PMC4227558 25211393

[b38] Albrecht J., Sidoryk-Wegrzynowicz M., Zielinska M. & Aschner M. Roles of glutamine in neurotransmission. Neuron Glia. Biol. 6, 263–276 (2010). 22018046 10.1017/S1740925X11000093

[b39] Wang K., Yamamoto H., Chin J. R., Werb Z. & Vu T. H. Epidermal growth factor receptor-deficient mice have delayed primary endochondral ossification because of defective osteoclast recruitment. J. Biol. Chem. 279, 53848–53856 (2004). 15456762 10.1074/jbc.M403114200 PMC2779713

[b40] Tsai W., Morielli A. D. & Peralta E. G. The m1 muscarinic acetylcholine receptor transactivates the EGF receptor to modulate ion channel activity. EMBO J. 16, 4597–4605 (1997). 9303304 10.1093/emboj/16.15.4597 PMC1170086

[b41] Duncan G. & Collison D. J. Role of the non-neuronal cholinergic system in the eye: a review. Life Sci. 72, 2013–2019 (2003). 12628451 10.1016/s0024-3205(03)00064-x

[b42] Wotta D. R., Wattenberg E. V., Langason R. B. & el-Fakahany E. E. M1, M3 and M5 muscarinic receptors stimulate mitogen-activated protein kinase. Pharmacology 56, 175–186 (1998). 9566019 10.1159/000028196

[b43] Huo L. . All-trans retinoic acid modulates mitogen-activated protein kinase pathway activation in human scleral fibroblasts through retinoic acid receptor beta. Mol. Vis. 19, 1795–1803 (2013). 23946634 PMC3742120

[b44] Arumugam B. & McBrien N. A. Muscarinic antagonist control of myopia: evidence for M4 and M1 receptor-based pathways in the inhibition of experimentally-induced axial myopia in the tree shrew. Invest. Ophthalmol. Vis. Sci. 53, 5827–5837 (2012). 22836762 10.1167/iovs.12-9943

[b45] Dietz H. C. & Pyeritz R. E. Mutations in the human gene for fibrillin-1 (FBN1) in the Marfan syndrome and related disorders. Hum. Mol. Genet. 1799–1809 (1995). 8541880 10.1093/hmg/4.suppl_1.1799

[b46] Farbrother J. E. . Linkage analysis of the genetic loci for high myopia on 18p, 12q, and 17q in 51 U.K. families. Invest. Ophthalmol. Vis. Sci. 45, 2879–2885 (2004). 15326098 10.1167/iovs.03-1156

[b47] Yip S. P. . A DNA pooling-based case-control study of myopia candidate genes COL11A1, COL18A1, FBN1, and PLOD1 in a Chinese population. Mol. Vis. 17, 810–821 (2011). 21527992 PMC3081793

[b48] French A. N., Morgan I. G., Mitchell P. & Rose K. A. Risk factors for incident myopia in Australian schoolchildren: the Sydney adolescent vascular and eye study. Ophthalmology 120, 2100–2108 (2013). 23672971 10.1016/j.ophtha.2013.02.035

[b49] Guggenheim J. A. . Time outdoors and physical activity as predictors of incident myopia in childhood: a prospective cohort study. Invest. Ophthalmol. Vis. Sci. 53, 2856–2865 (2012). 22491403 10.1167/iovs.11-9091 PMC3367471

[b50] Saw S. M. . Nearwork in early-onset myopia. Invest. Ophthalmol. Vis. Sci. 43, 332–339 (2002). 11818374

[b51] Guo Y. . Myopic shift and outdoor activity among primary school children: one-year follow-up study in Beijing. PLoS ONE 8, e75260 (2013). 24086484 10.1371/journal.pone.0075260 PMC3782472

[b52] Mutti D. O., Mitchell G. L., Moeschberger M. L., Jones L. A. & Zadnik K. Parental myopia, near work, school achievement, and children's refractive error. Invest. Ophthalmol. Vis. Sci. 43, 3633–3640 (2002). 12454029

[b53] Haworth C. M., Davis O. S. & Plomin R. Twins Early Development Study (TEDS): a genetically sensitive investigation of cognitive and behavioral development from childhood to young adulthood. Twin Res. Hum. Genet. 16, 117–125 (2013). 23110994 10.1017/thg.2012.91 PMC3817931

[b54] Lin Z. . Near work, outdoor activity, and their association with refractive error. Optom. Vis. Sci. 91, 376–382 (2014). 24637483 10.1097/OPX.0000000000000219

[b55] Lu B. . Associations between near work, outdoor activity, and myopia among adolescent students in rural China: the Xichang Pediatric Refractive Error Study report no. 2. Arch. Ophthalmol. 127, 769–775 (2009). 19506196 10.1001/archophthalmol.2009.105

[b56] French A. N., Ashby R. S., Morgan I. G. & Rose K. A. Time outdoors and the prevention of myopia. Exp. Eye Res. 114, 58–68 (2013). 23644222 10.1016/j.exer.2013.04.018

[b57] Howie B., Fuchsberger C., Stephens M., Marchini J. & Abecasis G. R. Fast and accurate genotype imputation in genome-wide association studies through pre-phasing. Nat. Genet. 44, 955–959 (2012). 22820512 10.1038/ng.2354 PMC3696580

[b58] Howie B. N., Donnelly P. & Marchini J. A flexible and accurate genotype imputation method for the next generation of genome-wide association studies. PLoS Genet. 5, e1000529 (2009). 19543373 10.1371/journal.pgen.1000529 PMC2689936

[b59] Aulchenko Y. S., Ripke S., Isaacs A. & van Duijn C. M. GenABEL: an R library for genome-wide association analysis. Bioinformatics 23, 1294–1296 (2007). 17384015 10.1093/bioinformatics/btm108

[b60] Voorman A., Lumley T., McKnight B. & Rice K. Behavior of QQ-plots and genomic control in studies of gene-environment interaction. PLoS ONE 6, e19416 (2011). 21589913 10.1371/journal.pone.0019416 PMC3093379

[b61] Manning A. K. . Meta-analysis of gene-environment interaction: joint estimation of SNP and SNP × environment regression coefficients. Genet. Epidemiol. 35, 11–18 (2011). 21181894 10.1002/gepi.20546 PMC3312394

[b62] Willer C. J., Li Y. & Abecasis G. R. METAL: fast and efficient meta-analysis of genomewide association scans. Bioinformatics 26, 2190–2191 (2010). 20616382 10.1093/bioinformatics/btq340 PMC2922887

[b63] Devlin B. & Roeder K. Genomic control for association studies. Biometrics 55, 997–1004 (1999). 11315092 10.1111/j.0006-341x.1999.00997.x

[b64] Kent W. J. . The human genome browser at UCSC. Genome Res. 12, 996–1006 (2002). 12045153 10.1101/gr.229102 PMC186604

[b65] Ward L. D. & Kellis M. HaploReg: a resource for exploring chromatin states, conservation, and regulatory motif alterations within sets of genetically linked variants. Nucleic Acids Res. 40, D930–D934 (2012). 22064851 10.1093/nar/gkr917 PMC3245002

[b66] Consortium E. P. . An integrated encyclopedia of DNA elements in the human genome. Nature 489, 57–74 (2012). 22955616 10.1038/nature11247 PMC3439153

[b67] Warde-Farley D. . The GeneMANIA prediction server: biological network integration for gene prioritization and predicting gene function. Nucleic Acids Res. 38, W214–W220 (2010). 20576703 10.1093/nar/gkq537 PMC2896186

[b68] Bowes Rickman C. . Defining the human macula transcriptome and candidate retinal disease genes using EyeSAGE. Invest. Ophthalmol. Vis. Sci. 47, 2305–2316 (2006). 16723438 10.1167/iovs.05-1437 PMC2813776

[b69] Rivas M. A. . Human genomics. Effect of predicted protein-truncating genetic variants on the human transcriptome. Science 348, 666–669 (2015). 25954003 10.1126/science.1261877 PMC4537935

