# Supplementary material for: Meta-analysis of gene–environment-wide association scans accounting for education level identifies additional loci for refractive error
Source: Nat Commun. 2016 Mar 29;7:11008. doi: 10.1038/ncomms11008 (PMC4820539; doi:10.1038/ncomms11008)
Supplement: Supplementary Information — Supplementary Figures 1-5, Supplementary Tables 1-10, Supplementary Notes 1-2 and Supplementary References [file ncomms11008-s1.pdf]

Supplementary Fig. 1 Quantile-quantile plots for the join meta-analysis (-log10(P_{JMA})) in Europeans, Asians and all cohorts

a) European Ancestry ($\lambda_{gc} = 1.081$)

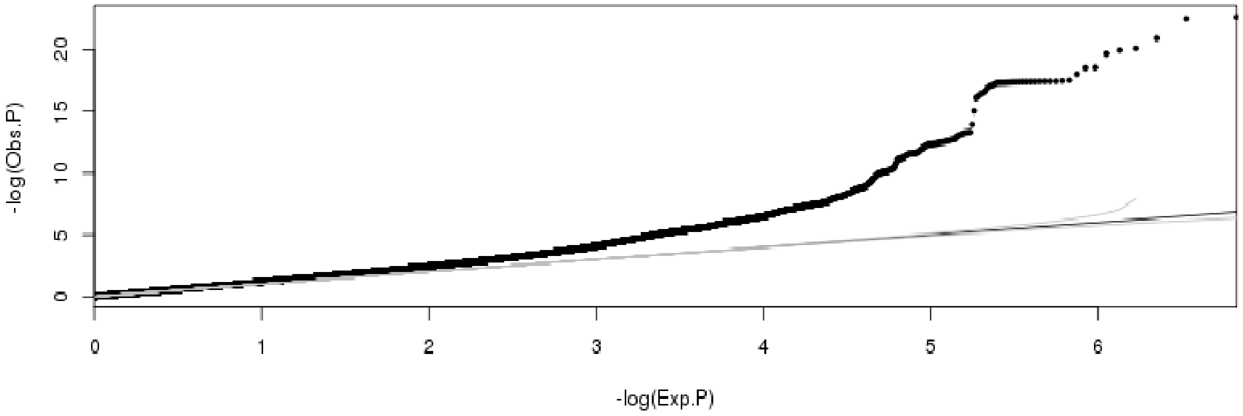

b) Asians ($\lambda_{gc} = 1.053$)

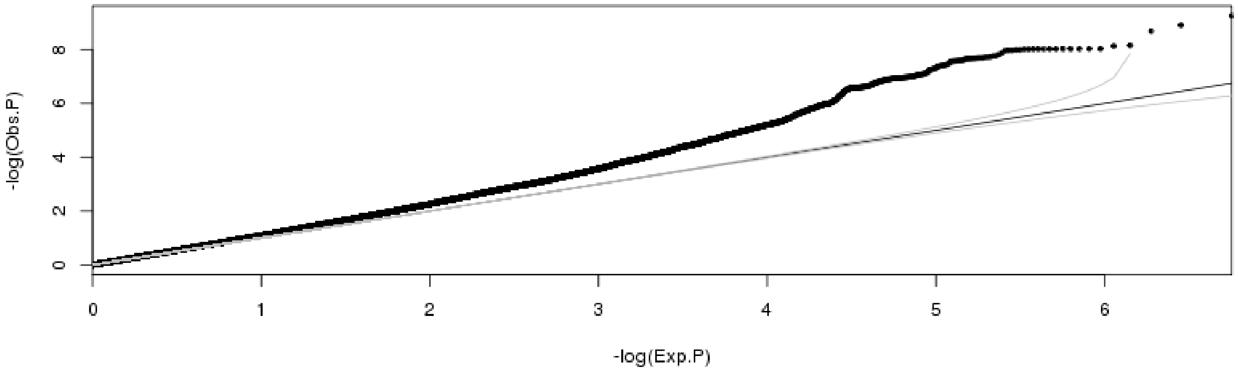

c) All ($\lambda_{gc} = 1.092$)

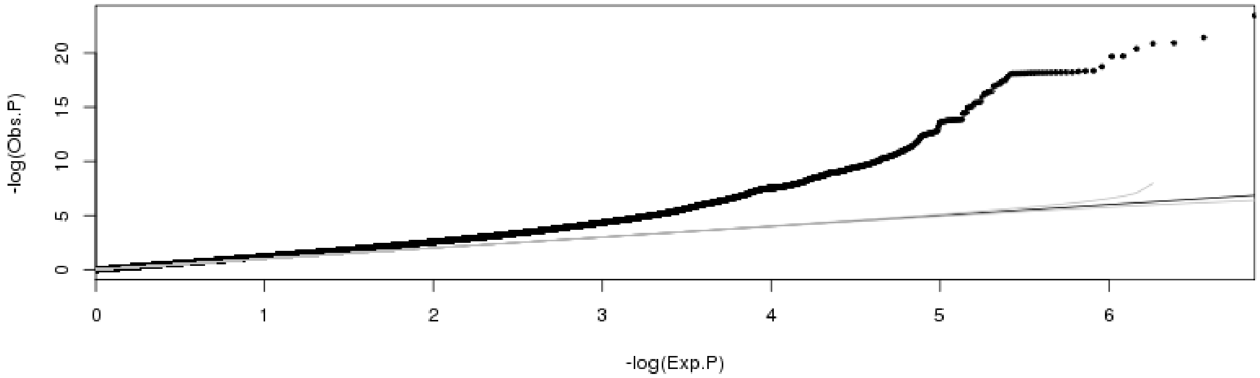

Supplementary Fig. 2 Regional association plots at the nine loci associated with spherical equivalent for the join meta-analysis testing (-log$_{10}$(P_{JAM})) in all (*a-f)* and Asian cohorts (*g-i*).

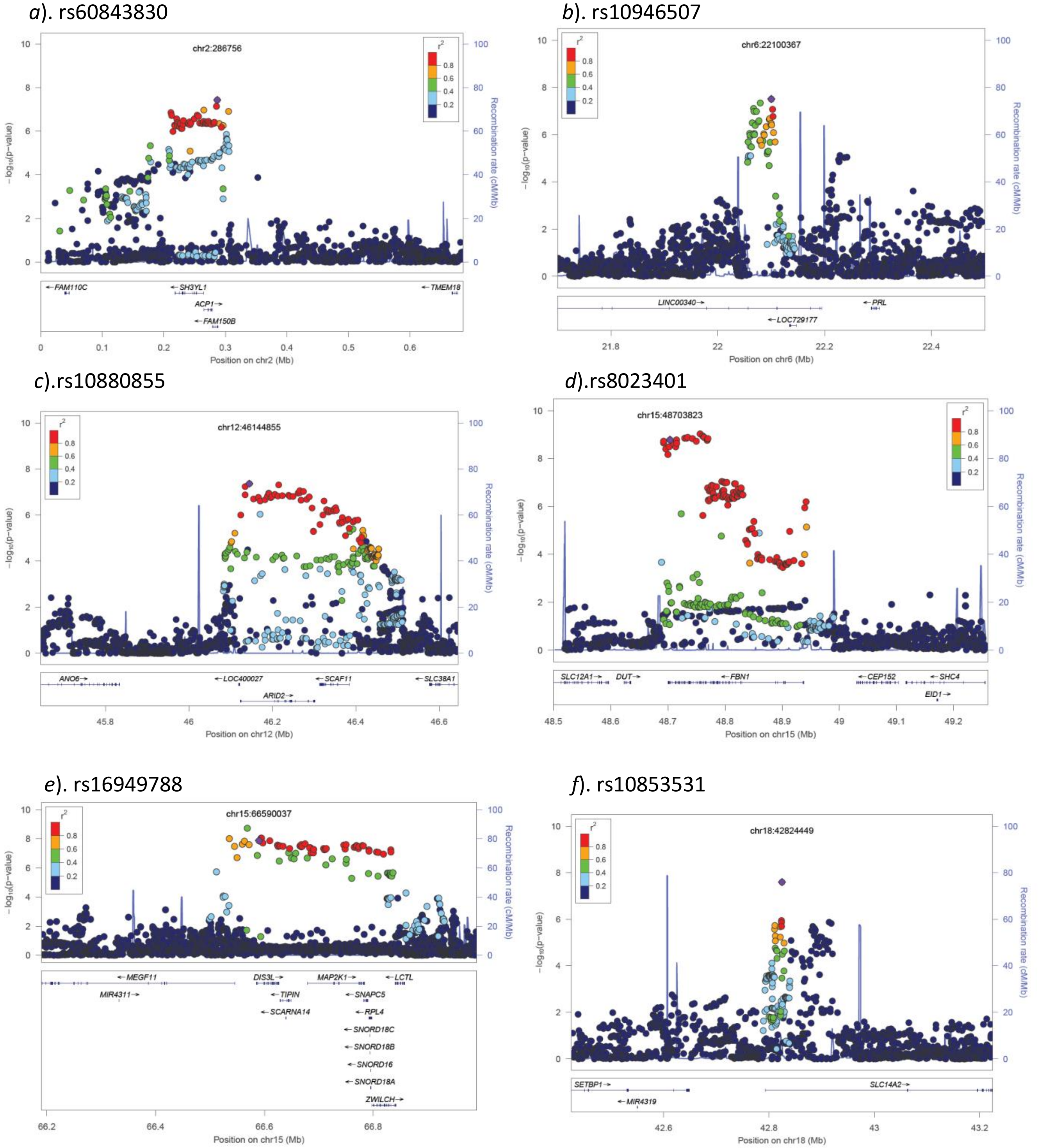

g). rs12511037

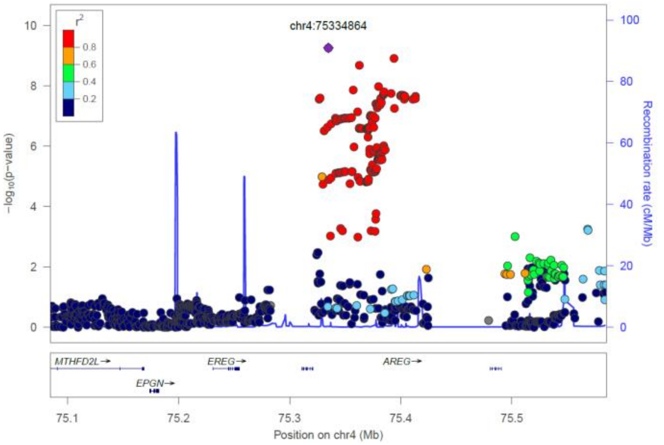

h). rs13215566

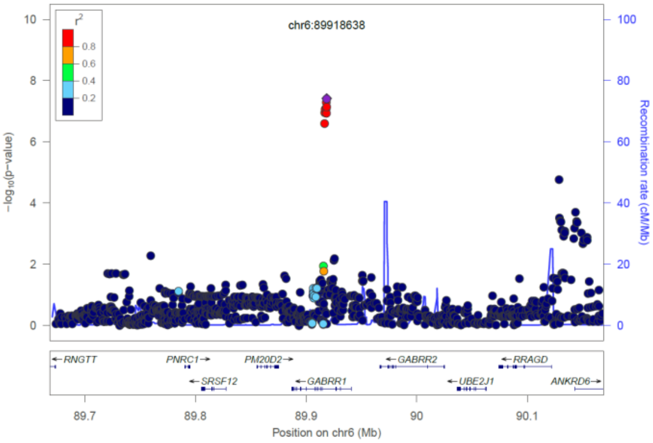

i). rs12206610

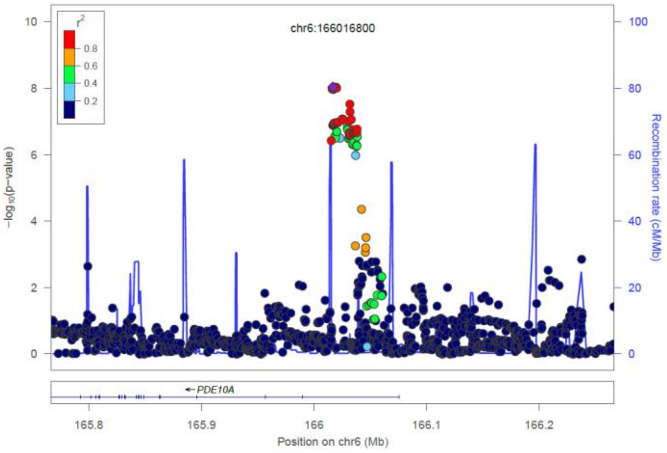

Supplementary Fig. 3 Scatter plot of SNP x education interaction effects for spherical equivalent at 39 known GWAS loci

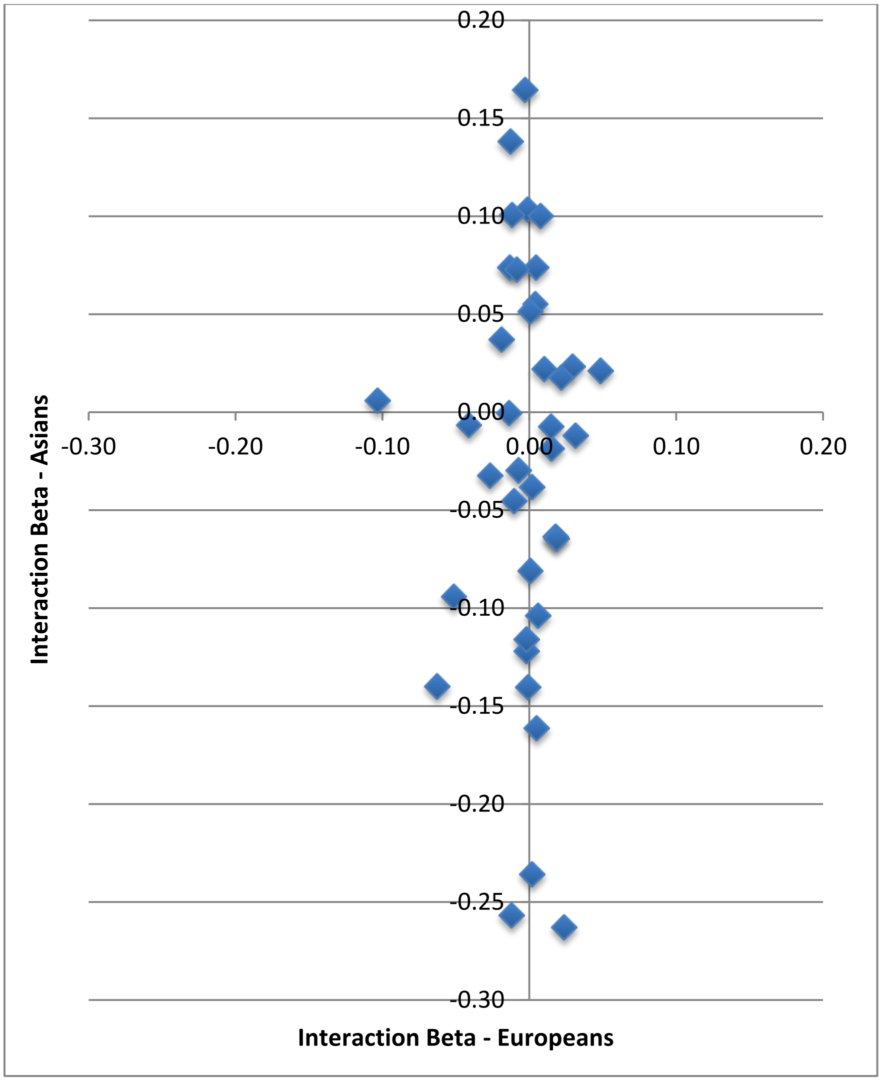

The interaction beta coefficient corresponds to the effect in diopters of one additional copy of the risk allele on spherical equivalent in the high versus low educational level. Thirty-nine index SNPs had larger SNP x education interaction effect on spherical equivalent in Asians versus Europeans (meta-regression *P* for fold changes < 0.001). For 20 SNPs with the same direction of the interaction effect, the magnitudes of interaction effects were 4-fold larger on average in Asians than in Europeans (*P* = 0.003). The *P*-value for the difference of interaction effects in Asian versus European samples was obtained from the meta-regression with the outcome as the fold-changes of the interaction beta coefficients in Asians as compared to Europeans.

Supplementary Fig. 4 Network analysis of the novel identified genes and known GWAS loci for myopia

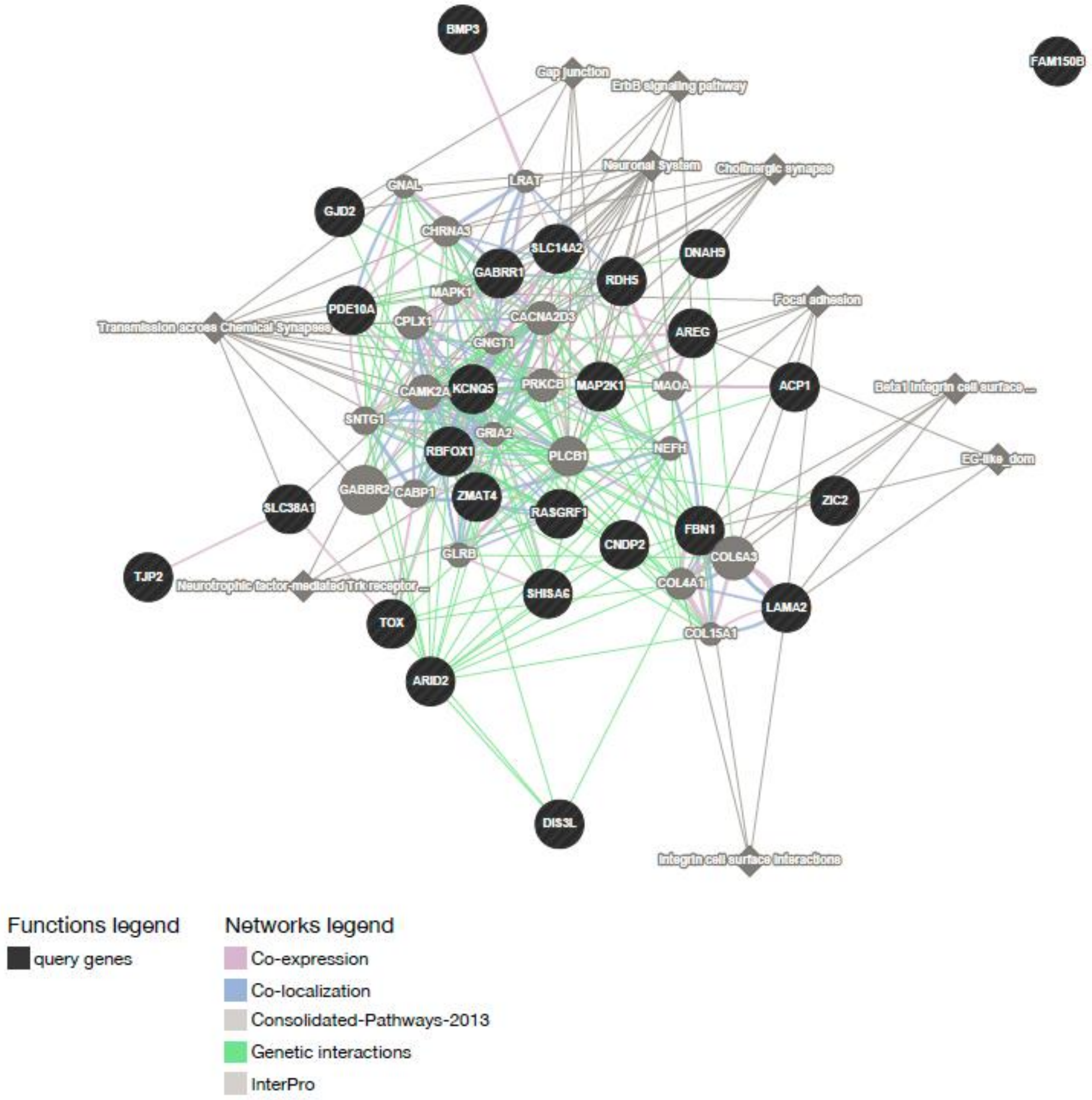

Network was generated based on the functional and biological connectivity, graphically represented by anodes (gene) and edges (connections), using the information provided by GeneMANIA database[1]. The novel 9 genetic loci (12 genes; *LINC00340* was not in database and hence omitted) identified in this study and 13 overlapping genes for spherical equivalent and age-at-onset of myopia GWAS from the CREAM and 23&Me[2; 3] were included for the analysis. The network weighting was assigned based on query genes, as the default method in GeneMANIA. The top three function categories are: ligand-gated channel activity (False Discovery Rate [FDR] = 0.175), neurotransmitter transport (FDR = 0.175) and extracellular matrix (FDR = 0.299). Connections are color-coded by interaction type. Purple line, co-expression (32.03%); Blue, co-localization (14.94%); Grey, shared standard pathway[4] (48.73%) or protein domain[5] (4.29%). The black circle represents the query gene, and grey circle represents additional gene predicated by GeneMANIA for the network.

Supplementary Fig. 5 Regional association plots of SNP x education interactions at three loci for spherical equivalent (-$\log_{10}(P_{int})$) in European (a, c, e) and Asian cohorts (b, d, f)

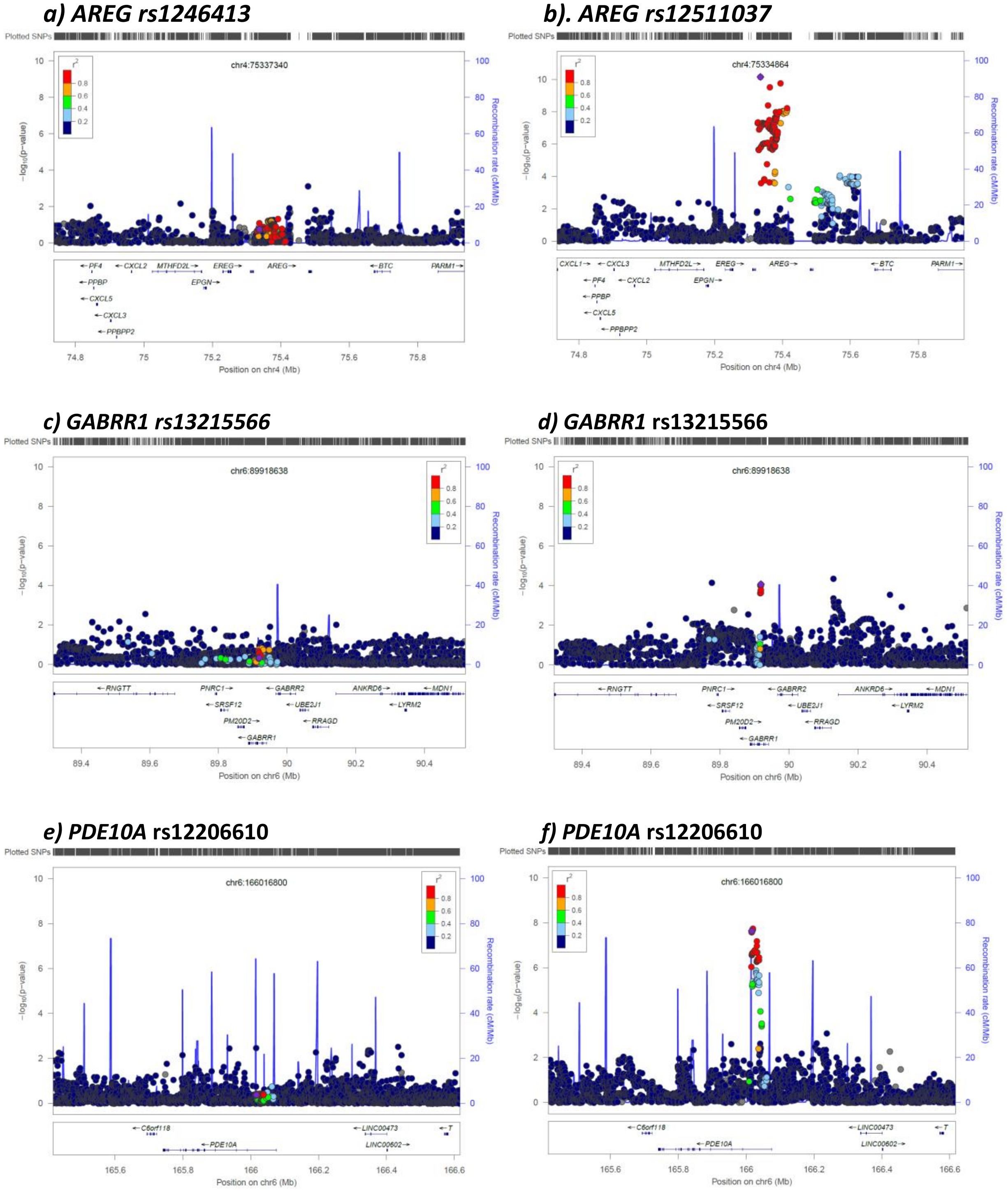

Note: SNP rs12511037 was absent in European genotype/imputed data and we thus presented the proxy SNP rs1246413 (T/G, frequency of risk allele T = 0.95) in LD with rs12511037 ($r^2 = 1$).

Supplementary Table 1. Description of study design, phenotyping and education levels

Study	Method of Measurement	Study design	Higher education (%)
**Europeans (*n* = 40,036)**			
ALIENOR	Speedy K Luneau, France	Population-based	45.4
ALSPAC	Canon R-50 autorefractor and subjective refraction	Family-based study	38.6
AREDS	Subjective Refraction	Population-based	94.4
BATS	Humphrey-598 Automatic Refractor (USA)	Twins	60.3
BMES	Humphrey autorefractor 530	Population-based	65.5
CROATIA-Korcula	Nidek ARK30 hand-held autorefractometer	Family-based	52.7
CROATIA-Split	Nidek ARK30 hand-held autorefractometer	Family-based	83.1
DCCT	Subjective Refraction	Clinic trial	86.2
EGCUT	Autorefraction measurement method; self-reported	Population-based	37.2
EPIC	Humphrey Auto-refractor 500	Population-based	62.7
ERF	Topcon RM-A2000 autorefractor	Family-based	29.5
FES	Subjective Refraction	Family-based	53.0
FITSA	Topcon AT (Tokyo, Japan)	Population-based	16.5
GHS1	Humphrey Automated Refractor/Keratometer (HARK) 599 (Germany)	Population-based	47.1
GHS2	Humphrey Automated Refractor/Keratometer (HARK) 599 (Germany)	Population-based	49.4
KORA	Nikon Retinomax	Population-based	26.5
OGP Talana	Topcon RK-8100 autorefractor	Family-based	16.5
ORCADES	Kowa KW 2000 autorefractometer	Family-based	54.0
RAINE	Nidek ARK-510A	Population-based	73.6
RS1	Topcon RM-A2000 autorefractor	Population-based	35.3
RS2	Topcon RM-A2000 autorefractor	Population-based	46.2
RS3	Topcon RM-A2000 autorefractor	Population-based	53.6
TwinsUK	ARM-10 autorefractor (Takagi Ltd)	Twins	46.7
WESDR	Subjective Refraction	Clinic trial	58.4
YFS	Nidek AR-310AR autorefractor	Population-based	85.7
**Asians (*n* = 10,315)**			
BES	Canon RK-5 Auto Ref-Keratometer	Population-based	14.0
Nagahama	NideK ARK-530A	Population-based	75.9
SCES-610K	Canon RK-5 Auto Ref-Keratometer	Population-based	21.3
SCES-OmniE	Canon RK-5 Auto Ref-Keratometer	Population-based	26.7
SiMES	Canon RK-5 Auto Ref-Keratometer	Population-based	6.7
SINDI	Canon RK-5 Auto Ref-Keratometer	Population-based	22.5
SP2-1M	Canon RK-5 Auto Ref-Keratometer	Population-based	45.0
SP2-610	Canon RK-5 Auto Ref-Keratometer	Population-based	37.4
STARS	Canon RK-5 Auto Ref-Keratometer	Population-based	55.7

A higher education group included those who had completed at least higher secondary education, polytechnic, or with ≥ 12 years spent in formal education (see Methods); cut-off >12 years of formal education was used for four cohorts of relatively young European participants (BATS, DCCT, RAINE and WESDER).

Supplementary Table 2. Description of genotyping, imputation, markers and genomic inflation factor (λ_{GC})

Study	Genotyping method	Imputation software	Analysis software	Markers	λ_{GC} for JMA
**Europeans**					
ALIENOR	Illumina HumanHap610-Quad	Minimac	Quicktest	6,463,430	1.049
ALSPAC	Illumina HumanHap660 W-Quad	Minimac	ProbABEL	6,182,596	1.009
AREDS	Illumina HumanOmni2.5-4v1_B	IMPUTE2	Quicktest	5,931,201	1.056
BATS	Illumina HumanHap610W Quad	Minimac	MIXABLE	4,130,050	1.125
BMES	Illumina HumanHap670 Quad	IMPUTE2	Quicktest	6,171,511	1.026
CROATIA-Korcula	Illumina Human370CNV-Quad	IMPUTE2	MIXABEL	6,434,958	1.054
CROATIA-Split	Illumina Human370CNV-Quad	IMPUTE2	MIxABEL	6,451,120	1.103
DCCT	Illumina Human1M-Omni	IMPUTE2	Quicktest	6,509,757	1.040
EGCUT	Illumina Human OMNIExpress	IMPUTE2	Quicktest	7,331,415	1.021
EPIC	Affymetrix GeneChip Human Mapping 500K	IMPUTE2	Quicktest	6,319,806	1.030
ERF	Illumina 6k, Illumina 318K, Illumina 370K and Affymetrix 250K	Minimac	MIXABEL	5,178,829	1.053
FES	Affymetrix 250K Mapping Nspl, 250K Mapping StyI, and HuGeneFocussed 50K	IMPUTE2	MIXABEL	5,702,554	1.012
FITSA	Illumina HumanHap300	IMPUTE2	Quicktest	5,681,882	1.109
GHS1	Affymetrix 6.0	IMPUTE2	ProbABEL	6,172,064	1.017
GHS2	Affymetrix 6.0	IMPUTE2	ProbABEL	6,180,291	1.021
KORA	Illumina HumanOmni2.5-4v1_B	IMPUTE2	Quicktest	6,491,491	1.030
OGP Talana	Affymetrix 500k array Chip	IMPUTE2	MIXABEL	6,065,503	1.115
ORCADES	Illumina HumanHap300 & Human370CNV-Quad	IMPUTE2	MIXABEL	5,839,085	1.043
RAINE	Illumina HumanHap610/660 Quad	Minimac	ProbABEl	5,840,716	1.097
RS1	Illumina Infinium II & HumanHap550	Minimac	ProbABEL	6,164,928	1.046
RS2	Illumina HumanHap550 Duo & HumanHap610-Quad	Minimac	ProbABEL	6,155,800	1.022
RS3	Illumina HumanHap610-Quad	Minimac	ProbABEL	5,786,654	1.024
TwinsUK	Illumina HumanHap300K-Duo & HumanHap610-Quad	IMPUTE2	Quicktest	6,438,502	1.021
WESDR	Illumina Human Omni 1-Quad	IMPUTE2 v2.3.0	Quicktest	6,491,238	1.047
YFS	Illumina HumanHap 670k BeadChip	IMPUTE2	Quicktest	6,512,211	1.038
**Asians**					
BES	Illumina HumanHap610-Quad	Minimac	Quicktest	5,612,061	1.093
Nagahama	HumanHap610KQuad, HumanOmni2.5M, HumanExome	Minimac	Quicktest	5,093,159	1.047
SCES-610K	Illumina HumanHap610-Quad	Minimac	Quicktest	5,581,092	1.052
SCES-OmniE	Illumina HumanOmnioExpress	Minimac	Quicktest	5,617,278	1.072
SiMES	Illumina HumanHap610-Quad	Minimac	Quicktest	5,672,571	1.049
SINDI	Illumina HumanHap610-Quad	Minimac	Quicktest	6,149,366	1.046

SP2-1M	Illumina HumanHap610-Quad	Minimac	Quicktest	5,717,698	1.022
SP2-610	Illumina HumanHap610-Quad	Minimac	Quicktest	5,586,915	1.043
STARS	Illumina HumanHap610-Quad	Minimac	Quicktest	5,584,216	1.022

JMA – Joint meta-analysis

Supplementary Table 3. Previously 17 implicated loci identified from the joint meta-analysis in the combined data

SNP	CHR	POS	Gene	Allele	All (n = 50,351) MAF	All (n = 50,351) P_{JMA}	Europeans (n=40,036) MAF	Europeans (n=40,036) P_{JMA}	Asians (n = 10,315) MAF	Asians (n = 10,315) P_{JMA}
rs891378	1	207490319	CD55	A/G	0.43	3.17E-12	0.43	1.72E-08	0.41	1.30E-04
rs2573210	2	233280565	CHRNG-ALPPL2	A/G	0.33	2.79E-10	0.38	2.55E-10	0.12	2.97E-01
rs7744813	6	73643289	KCNQ5	A/C	0.43	3.50E-19	0.47	9.43E-16	0.27	2.46E-04
rs12193446*	6	129820038	LAMA2	A/G	0.09	1.20E-21	0.09	1.20E-21	0.09	9.33E-01
rs2137277	8	40734662	ZMAT4	A/G	0.18	3.99E-09	0.20	1.18E-07	0.10	1.62E-03
rs10089517	8	60178721	TOX	A/C	0.36	8.48E-18	0.35	1.17E-14	0.38	6.74E-04
rs11145488	9	71770939	TJP2	A/G	0.21	1.56E-09	0.22	1.65E-09	0.09	8.56E-01
rs1649081	10	60292444	BICC1	A/G	0.49	2.66E-09	0.48	2.99E-07	0.48	6.60E-03
rs3138142	12	56115585	RDH5	T/C	0.20	1.14E-08	0.20	3.57E-08	0.13	2.11E-01
14:54579969:A_AC	14	54579969	BMP4	D/R	0.32	9.62E-09	0.34	2.52E-06	0.37	2.19E-03
rs2753462	14	60850703	SIX6	C/G	0.29	4.37E-09	0.26	3.88E-08	0.38	3.39E-02
rs524952	15	35005886	GJD2	A/T	0.46	1.01E-25	0.47	2.23E-23	0.45	1.31E-04
rs6495367	15	79375347	RASGRF1	A/G	0.46	3.89E-08	0.42	5.34E-06	0.39	8.62E-04
rs6500957	16	7462045	A2BP1	C/G	0.34	2.86E-09	0.38	3.40E-08	0.12	2.76E-02
rs2908972	17	11407259	DNAH9	A/T	0.42	2.73E-12	0.41	2.63E-10	0.46	4.60E-04
rs72483203	17	30463885	MYO1D-TMEM98	A/G	0.09	8.81E-09	0.06	4.82E-10	0.17	9.32E-06
rs929474	17	68724036	KCNJ2	A/G	0.42	1.66E-09	0.43	2.58E-11	0.40	3.37E-01

CHR, chromosome; MAF, minor allele frequency. P_{JMA}, *p*-value for join meta-analysis. Alleles are presented as effect allele/other allele.
**LAMA2* rs12193446 was omitted in Asian cohorts due to low MAF (MAF < 0.01). A proxy SNP rs9402138 was used for the JMA testing in Asians.

Supplementary Table 4. Results of meta-regression showing the associations of study-level characteristics with the SNP × education interaction effect on spherical equivalent

**Study-level characteristics**	***GABRR1* (rs13215566)**		***PDE10A* (rs12206610)**	
	**Effect**	***P***	**Effect**	***P***
Sample size	-	0.662	-	0.636
Average spherical equivalent, D	-	0.205	-	0.025
Proportion of high education group, %	+	0.480	-	0.064
Ethnicity, Asian vs. European	-	0.006	-	0.042
Study year	-	0.409	+	0.397
Study design	+	0.990	-	0.836
Average age ≥ 40 vs. <40, years	+	0.057	-	0.285
Education main effect on spherical equivalent, higher vs. lower education	-	0.158	-	0.138

The *p*-values were obtained from the meta-regression model, including all the covariates listed above. Study year, the year in the middle of the study period; Study design, independent samples from population-based studies/clinic trials vs. related samples from family-based studies/twin studies. Meta-regression analysis included all 34 studies listed in Table 1.

Supplementary Table 5. Associations between three G x E loci and education in Asian cohorts

**SNP**	**A1/A2**	**Gene**	**OR**	**95% CI of OR**		***P***	**P_{het}**
rs12511037	C/T	AREG	0.90	0.79	1.02	0.102	0.353
rs13215566	C/G	*GABRR1*	0.95	0.81	1.10	0.467	0.170
rs12206610	C/T	*PDE10A*	0.95	0.84	1.06	0.355	0.900

Logistic regression for education on three SNPs was performed in Asian studies (total *n* = 10,315): SCES-610K, SCES-OmniE, SiMES, SINDI, SP2-1M, SP2-610, STARS, BES and Nagahama study adjusted for age, gender, and population stratification (SiMES and SINDI). The odds ratio (OR) was estimated from the meta-analysis of the results from above studies. Education level was defined as 1 = higher education, 0 = lower education. A1/A2: Effect allele/reference allele.

Supplementary Table 6. SNP x education interaction for spherical equivalent at GWAS identified top loci

						Europeans (n = 40,036)				Asians (n = 10,315)				All (n = 50,351)			
**SNP**	**CHR**	**BP**	**Gene**	**A1**	**A2**	**MAF**	β_{int}	**s.e**	P_{int}	**MAF**	β_{int}	**s.e**	P_{int}	**MAF**	β_{int}	**s.e**	P_{int}
rs1652333	1	207470460	*CD55*	G	A	0.32	0.002	0.027	0.948	0.40	0.122	0.088	0.165	0.35	-0.012	0.026	0.637
rs4373767	1	219759682	*ZC3H11B*	T	C	0.42	0.010	0.019	0.591	0.38	0.022	0.087	0.802	0.41	0.011	0.018	0.563
rs17412774	2	146773948	*PABPCP2*	A	C	0.45	-0.052	0.024	0.029	0.36	-0.094	0.090	0.294	0.43	-0.054	0.023	0.017
rs17428076	2	172851936	*DLX1*	C	G	0.33	0.032	0.023	0.176	0.16	-0.012	0.155	0.938	0.28	0.031	0.023	0.185
rs1898585	2	178660450	*PDE11A*	T	C	0.28	0.018	0.027	0.515	0.32	-0.064	0.098	0.518	0.29	0.012	0.026	0.649
rs1656404	2	233379941	*PRSS56*	A	G	0.29	0.001	0.035	0.988	0.13	-0.081	0.255	0.751	0.25	-0.001	0.035	0.977
rs1881492	2	233406998	*CHRNG*	T	G	0.23	-0.001	0.035	0.975	0.13	-0.140	0.157	0.370	0.20	-0.008	0.034	0.821
rs14165	3	53847408	*CACNA1D*	G	A	0.33	-0.002	0.022	0.937	0.01				0.27	0.000	0.022	0.989
rs13091182	3	141133960	*ZBTB38*	G	A	0.40	-0.034	0.025	0.162	0.00				0.32	0.042	0.024	0.087
rs9307551	4	80530671	*LOC100506035*	A	C	0.25	-0.008	0.037	0.840	0.47	-0.030	0.086	0.729	0.31	-0.011	0.034	0.747
rs5022942	4	81959966	*BMP3*	A	G	0.25	-0.013	0.029	0.646	0.40	0.074	0.092	0.423	0.29	-0.006	0.028	0.843
rs7744813	6	73643289	*KCNQ5*	A	C	0.42	-0.027	0.028	0.336	0.30	-0.032	0.098	0.742	0.38	-0.027	0.027	0.310
rs9492338	6	129842538	*LAMA2*	C	G	0.23	-0.041	0.030	0.173	0.09	-0.007	0.161	0.967	0.19	-0.040	0.030	0.178
rs7829127	8	40726394	*ZMAT4*	A	G	0.31	-0.063	0.035	0.068	0.10	-0.140	0.141	0.322	0.25	-0.067	0.034	0.045
rs7837791	8	60179086	*TOX*	G	T	0.49	-0.018	0.023	0.426	0.43	0.065	0.089	0.466	0.47	0.013	0.022	0.558
rs4237036	8	61701057	*CHD7*	T	C	0.35	0.005	0.023	0.842	0.38	0.074	0.105	0.484	0.36	0.008	0.022	0.732
rs11145488	9	71770939	*TJP2*	A	G	0.31	-0.104	0.038	0.007	0.09	0.006	0.274	0.983	0.24	-0.101	0.038	0.008
rs7042950	9	77149837	*RORB*	G	A	0.31	-0.048	0.033	0.139	0.31	-0.021	0.098	0.829	0.31	0.046	0.031	0.141
rs7084402	10	60265404	*BICC1*	G	A	0.48	-0.029	0.025	0.238	0.47	-0.023	0.087	0.789	0.48	0.029	0.024	0.227
rs6480859	10	79081948	*KCNMA1*	T	C	0.42	0.022	0.020	0.291	0.15	0.018	0.120	0.883	0.34	0.022	0.020	0.287
rs745480	10	85986554	*RGR*	G	C	0.49	0.019	0.019	0.312	0.41	-0.037	0.087	0.668	0.47	-0.017	0.019	0.370
rs10882165	10	94924324	*CYP26A1*	T	A	0.44	0.002	0.020	0.914	0.24	0.116	0.171	0.498	0.38	-0.004	0.020	0.854
rs1381566	11	40149607	*LRRC4C*	G	T	0.30	-0.004	0.038	0.917	0.33	-0.055	0.110	0.613	0.31	0.009	0.036	0.792
rs2155413	11	84634790	*DLG2*	A	C	0.47	-0.001	0.024	0.960	0.26	0.104	0.104	0.318	0.41	0.004	0.023	0.859
rs11601239	11	105556598	*GRIA4*	C	G	0.47	0.015	0.017	0.384	0.42	-0.019	0.087	0.832	0.46	0.014	0.017	0.417
rs3138142	12	56115585	*RDH5*	C	T	0.33	-0.024	0.034	0.491	0.05	0.263	0.297	0.376	0.28	0.020	0.034	0.560
rs12229663	12	71249996	*PTPRR*	A	G	0.32	-0.011	0.023	0.639	0.38	-0.045	0.091	0.619	0.34	-0.013	0.022	0.566
rs8000973	13	100691367	*ZIC2*	C	T	0.48	0.014	0.027	0.606	0.27	0.000	0.103	0.997	0.42	-0.013	0.026	0.617
rs2184971	13	100818092	*PCCA*	A	G	0.46	-0.013	0.024	0.598	0.28	0.138	0.098	0.159	0.41	-0.004	0.024	0.863
rs66913363	14	54413001	*BMP4*	G	C	0.49	-0.015	0.020	0.461	0.34	0.007	0.105	0.945	0.44	0.014	0.020	0.477
rs1254319	14	60903757	*SIX6*	A	G	0.36	0.000	0.026	0.987	0.36	0.051	0.090	0.569	0.36	0.004	0.025	0.863

rs524952	15	35005886	*GJD2*	A	T	0.47	0.006	0.023	0.802	0.44	-0.104	0.087	0.231	0.46	-0.002	0.022	0.947
rs4778879	15	79372875	*RASGRF1*	G	A	0.45	-0.007	0.026	0.779	0.42	-0.100	0.090	0.264	0.44	0.015	0.025	0.560
rs17648524	16	7459683	*A2BP1*	C	G	0.40	0.005	0.023	0.833	0.20	-0.161	0.147	0.273	0.34	0.001	0.023	0.970
rs2969180	17	11407901	*SHISA6-DNAH9*	A	G	0.39	-0.012	0.028	0.667	0.46	-0.257	0.089	0.004	0.41	-0.034	0.027	0.203
rs17183295	17	31078272	*MYO1D*	T	C	0.29	-0.012	0.028	0.672	0.01				0.26	-0.011	0.028	0.704
rs4793501	17	68718734	*KCNJ2*	T	C	0.43	-0.003	0.023	0.898	0.42	0.164	0.089	0.064	0.43	0.008	0.023	0.731
rs12971120	18	72174023	*CNDP2*	A	G	0.32	0.002	0.024	0.942	0.29	-0.038	0.094	0.684	0.31	-0.001	0.023	0.976
rs235770	20	6761765	*BMP2*	T	C	0.39	-0.009	0.022	0.696	0.31	0.073	0.103	0.480	0.37	-0.005	0.022	0.815

β_{int}, Beta regression coefficient for SNP and education interaction on spherical equivalent; P_{int}, *p-value* for interaction between SNP and education on spherical equivalent; A1-risk allele; A2- reference allele.

Supplementary Table 7. Meta-analysis of SNP x nearwork interaction for spherical equivalent in pediatric cohorts at three index SNPs

SNP	Gene	A1	A2	Effect	s.e.	P_{int}	Direction	P_{het}
rs12511037	*AREG*	C	T	0.045	0.173	0.795	+-+	0.062
rs13215566	*GABRR1*	C	G	-0.088	0.066	0.309	---	0.655
rs12206610	*PDE10A*	C	T	-0.189	0.088	0.032	---	0.658

Meta-analysis of SNP x near work was performed in Chinese children from SCORM[6] (n = 988), Guangzhou twins[7] (n = 1,055) and European children in ALSAPC[8; 9] (n = 3,792). Near work is a binary variable, defined as 0 = low and 1= high, relative to the median number of hours per week spent reading, writing, computer or video games. Only near work activity outside of the regular school day was included. SCORM: Singapore Cohort study Of the Risk factors for Myopia; ALSPAC: Avon Longitudinal Study of Parents and Children. Genotyping GWAS were available from three cohorts.

Supplementary Table 8. Gene expression of identified loci in human ocular tissues

	Expression (PLIER)			
**GENE**	**Retina**	**Sclera**	**Choroid/RPE**	**Cornea**
FAM150B	29.94	62.13	333.33	33.62
ACP1	155.25	113.54	103.53	244.63
SH3YL1	34.29	42.23	65.09	101.85
LINC00340	na	na	na	na
FBN1	12.88	75.26	47.08	23.99
MAP2K1	85.72	91.26	183.61	78.81
DIS3L	43.20	32.95	42.16	40.83
ARID2	na	na	na	na
SNAT1 (SLC38A1)	236.69	40.76	71.16	190.82
SLC14A2	29.96	34.87	33.69	34.52
AREG	21.31	26.04	29.64	27.30
GABRR1	121.66	21.48	31.43	21.19
PDE10A	28.19	18.87	21.46	14.74

Expression data was obtained from Ocular Tissue Database[10], indicated as Affymetrix Probe Logarithmic Intensity Error (PLIER) normalized levels. The normalization of gene expression was calculated at both the probe set and metaprobe set levels with GC-background correction. The Affymetrix GeneChip Human Exon 1.0 ST (HuEx 1.0) microarrays were used to assess gene expression.

Supplementary Table 9. Genes harboring index SNPs or nearest genes of biological interest for myopia

SNP	Gene	Function
rs60843830	*FAM150B-ACP1*	*FAM150B* encodes family with sequence similarity 150, member B. It stimulates leukocyte tyrosine kinase (LTK) phosphorylation[11]. ACP1 (acid phosphatase 1) belongs to the phosphoprotein tyrosine phosphatase family that dephosphorylates platelet-derived growth factor receptor (PDGFR)[12].PDGFR is implicated in corneal proliferation[13]. The index SNP is in strong LD (r^2 = 0.99) with a missense coding variant rs11553746 (Thr95IIe) in *ACP1*.
rs10946507	*LINC00340 (6p22.3)*	*LINC00340* (Aliases: *CASC15*) is a non-protein coding RNA 340. Diseases associated with *LINC00340* include neuroblastoma[14].
rs8023401	*FBN1*	*FBN1* (fibrillin 1) encodes the extracellular matrix glycoprotein, a structural component of calcium-binding microfibrils[15]. The variant rs16960901, in moderate LD with the index SNP rs8023401 (r^2 = 0.45), is reported to have a cis-acting association with *FBN1* transcript levels (P = 7.5×10^{-7}) in whole blood[16].
rs16949788	*DIS3L-MAP2K1*	*DIS3L* encodes DIS3 like exosome 3'-5' exoribonuclease, a putative cytoplasm-specific catalytic component of the RNA exosome complex, involving in 3'-5'-exoribonuclease activity and RNA binding. *MAP2K1* encodes a mitogen-activated protein (MAP) kinase. MAP is involved in many cellular processes such as scleral fibroblasts, proliferation and transcription regulation[17].
rs10880855	*ARID2-SNAT1*	*ARID2* (AT rich interactive domain 2) facilitates ligand-dependent transcriptional activation. SNP rs10880855 has a nominal association with *ARID2* transcript level (P = 0.047) in skin tissues[18]. *SNAT1* (Aliases: *SLC38A1*) supplies glutamine to the synthesis of glutamatergic and GABAergic neurons[19]. The variant rs12827763 is associated with trans-acting expression of *SNAT1* (P = 1.3×10^{-8}) in muscle skeletal tissues[20], and is in low LD with the index SNP rs10880855 (r^2 = 0.15).
rs10853531	*SLC14A2*	*SLC14A2* (Aliases: *SETBP1*) encodes solute carrier family 14 member 2. SLC14A2 has the role as the transport of glucose, organic acids, metal irons and amine compounds.
rs12511037	*AREG*	*AREG* encodes amphiregulin, a ligand of the epidermal growth factor receptor (EGFR). EGFR promotes the growth of normal epithelial cells and is implicated in myopia progression through the muscarinic system[21; 22].
rs13215566	*GABRR1*	*GABRR1* encodes gamma-aminobutyric acid (GABA) C receptor ρ1. $GABA_C$ ρ1 is involved in the neurotransmission in the retina. The variant rs13215029, in perfect LD (r^2 = 1) with the index SNP rs13215566, is associated with cis-acting expression of *GABRR1* (P = 2.3×10^{-4}) in skin tissues[18]. Another variant rs6902106 (r^2 = 0.45) is associated with cis-acting expression of *GABRR1* (P = 2.5×10^{-7}) in artery tibial tissues [16].
rs12206610	*PDE10A*	*PDE10A* encodes phosphodiesterase, hydrolyzing both cAMP and cGMP to the monophosphate[23]. The levels of PDE10A protein display circadian rhythms at retinal photoreceptors[24], suggesting its potential roles in the visual circle.

Supplementary Table 10. Regulatory function for the index SNP and SNPs in the linkage disequilibrium ($r^2 \geq 0.8$)

Query SNP: rs12511037 and variants with r^2 >= 0.8 in Asians

variant	Promoter histone marks	Enhancer histone marks	DNAse	Proteins bound	Motifs changed	GENCODE genes	dbSNP func annot
rs1691275				KAP1	DMRT5,Foxp1,Nkx3	5.9kb 3' of AREG	
rs1797569					7 altered motifs	6.8kb 3' of AREG	
rs1691276					5 altered motifs	9.2kb 3' of AREG	
rs1389963					CEBPB,Hoxd8,Pax-4	10kb 3' of AREG	
rs1797577					CHOP::CEBPalpha,ZEB1	13kb 3' of AREG	
**rs12511037**					CEBPG	14kb 3' of AREG	
rs2609203					5 altered motifs	15kb 3' of AREG	
rs3913031	NHEK	HMEC			BHLHE40,E2A	16kb 3' of AREG	
rs1246414		HMEC, NHEK, K562	NHEK,HPDE6-E6E7,HEEpiC		CEBPG	16kb 3' of AREG	
rs1246413		NHEK, K562, HMEC				17kb 3' of AREG	
rs2609204		NHEK, HMEC			6 altered motifs	21kb 3' of AREG	
rs1494876		NHEK			TATA,TCF12	21kb 3' of AREG	
rs1494877		NHEK			CTCF,Foxo	21kb 3' of AREG	
rs1269733						22kb 3' of AREG	
rs200981664					6 altered motifs	22kb 3' of AREG	
rs1811651						22kb 3' of AREG	
rs1246407					7 altered motifs	23kb 3' of AREG	
rs1246406		HepG2	LNCaP	5 bound proteins	5 altered motifs	24kb 3' of AREG	
rs1826695					NF-AT,NF-I,RBP-Jkappa	25kb 3' of AREG	
rs1389959					4 altered motifs	25kb 3' of AREG	
rs2125399		NHEK, HMEC				25kb 3' of AREG	
rs1546145		HMEC			Nkx3,Zec	26kb 3' of AREG	
rs12507794					5 altered motifs	27kb 3' of AREG	
rs4694686					CIZ,Nkx2,PLZF	27kb 3' of AREG	
rs2643006		K562, HMEC, NHEK		GATA2,TAL1	FXR	28kb 3' of AREG	
rs2609201		HMEC, K562, NHEK	HEEpiC,PrEC,SAEC	PU1,EGR1	6 altered motifs	29kb 3' of AREG	
rs1246396					4 altered motifs	29kb 3' of AREG	
rs1027371					Pax-4,Sox,TCF4	30kb 3' of AREG	
rs1027372						30kb 3' of AREG	
rs1246398					6 altered motifs	33kb 3' of AREG	
rs1845570						33kb 3' of AREG	
rs1494872					4 altered motifs	34kb 3' of AREG	
rs140559873		NHEK			Evi-1	35kb 3' of AREG	
rs1826696		NHEK			4 altered motifs	35kb 3' of AREG	
rs2643007		NHEK			Dobox4,SIX5	35kb 3' of AREG	
rs959490		HMEC, NHEK			5 altered motifs	35kb 3' of AREG	
rs1494891		HMEC, NHEK				36kb 3' of AREG	
rs1494892		HMEC, NHEK			CCNT2,GATA	36kb 3' of AREG	
rs2201455		HMEC, NHEK			4 altered motifs	37kb 3' of AREG	
rs2643009					Cdx2,Pdx1	37kb 3' of AREG	
rs202222740					Foxo,Foxp1,TATA	40kb 3' of AREG	
rs2934879					Foxp1,Pou1f1,Pou3f2	41kb 3' of AREG	
rs2643001					8 altered motifs	41kb 3' of AREG	
rs1603131					11 altered motifs	42kb 3' of AREG	
rs1603132					Foxa,HNF1	42kb 3' of AREG	
rs79077380					TATA	42kb 3' of AREG	
rs1797586					Nkx2	42kb 3' of AREG	
rs969911					5 altered motifs	43kb 3' of AREG	
rs2643002					Fox,Foxc1,Pou2f2	43kb 3' of AREG	
rs1246405						44kb 3' of AREG	
rs1389955		HSMM, HMEC, Huvec				48kb 3' of AREG	
rs1389956		HSMM, HMEC, Huvec			STAT	48kb 3' of AREG	
rs1389957		HSMM, HMEC, Huvec			Brachyury,Mef2	48kb 3' of AREG	
rs1389958		5 cell types			TATA	48kb 3' of AREG	
rs1039011		5 cell types	18 cell types	GATA2	BDP1,EBF,p53	49kb 3' of AREG	
rs1021519		5 cell types	48 cell types	4 bound proteins	RBP-Jkappa,SETDB1,Znf143	49kb 3' of AC142293.3	
rs1021520		5 cell types			13 altered motifs	49kb 3' of AC142293.3	
rs1021521		5 cell types	HBMEC		Foxp1,Hoxb13,Sox	48kb 3' of AC142293.3	

rs3113925		5 cell types	21 cell types	CJUN,STAT3	Irf,Pou5f1	48kb 3' of AC142293.3
rs3113926		5 cell types	33 cell types	CJUN,STAT3	4 altered motifs	48kb 3' of AC142293.3
rs3113927		4 cell types	11 cell types	CJUN,STAT3	E2F	48kb 3' of AC142293.3
rs3113928		4 cell types	AoAF,HConF,HMVEC-LLy	CJUN,STAT3	GR,Pou1f1,Pou2f2	48kb 3' of AC142293.3
rs3113929		4 cell types		CJUN	FAC1,HNF4,SIX5	48kb 3' of AC142293.3
rs1587079		Huvec, NHLF, HMEC			6 altered motifs	48kb 3' of AC142293.3
rs76884596		Huvec, NHLF, HMEC			14 altered motifs	48kb 3' of AC142293.3
rs1587081		Huvec, NHLF, HMEC			22 altered motifs	48kb 3' of AC142293.3
rs4566628		Huvec, NHLF, HMEC			Spz1	47kb 3' of AC142293.3
rs3104112		Huvec, NHLF, HMEC			MZF1::1-4	47kb 3' of AC142293.3
rs3113934		Huvec, NHLF, HMEC			SETDB1	47kb 3' of AC142293.3
rs3104113		Huvec, NHLF, HMEC				47kb 3' of AC142293.3
rs3113935	HepG2	5 cell types	5 cell types	CFOS	Pax-4	47kb 3' of AC142293.3
rs4257635	HepG2	Huvec			CTCF	47kb 3' of AC142293.3
rs1845567	HepG2	Huvec				46kb 3' of AC142293.3
rs1845568					Ik-2,NF-AT,p300	46kb 3' of AC142293.3
rs3104116					SRF	46kb 3' of AC142293.3
rs3104117						46kb 3' of AC142293.3
rs3113938					PEBP	46kb 3' of AC142293.3
rs150324694					Gm397	45kb 3' of AC142293.3
rs113523577					12 altered motifs	45kb 3' of AC142293.3
rs145095604					4 altered motifs	45kb 3' of AC142293.3
rs72862607					Gm397	45kb 3' of AC142293.3
rs115834274						45kb 3' of AC142293.3
rs4694688					6 altered motifs	45kb 3' of AC142293.3
rs3113939					Pou3f2,p300	44kb 3' of AC142293.3
rs145593785					CEBPD,NF-Y	44kb 3' of AC142293.3
rs72862614		K562		JUND	6 altered motifs	43kb 3' of AC142293.3
rs140937898		K562	5 cell types	PU1,CJUN,JUND	Foxp3	43kb 3' of AC142293.3
rs138303263						43kb 3' of AC142293.3
rs116834404						43kb 3' of AC142293.3
rs143145182					7 altered motifs	42kb 3' of AC142293.3
rs114334721					RFX5	42kb 3' of AC142293.3
rs147478385					5 altered motifs	42kb 3' of AC142293.3
rs3113917					5 altered motifs	41kb 3' of AC142293.3
rs3104121					15 altered motifs	41kb 3' of AC142293.3
rs3104122					4 altered motifs	41kb 3' of AC142293.3
rs3113918					6 altered motifs	41kb 3' of AC142293.3
rs1971299					NRSF	41kb 3' of AC142293.3
rs3104123					Mef2,Pou5f1	40kb 3' of AC142293.3
rs1908423					Irf,RXRA	40kb 3' of AC142293.3
rs3113919					13 altered motifs	40kb 3' of AC142293.3
rs1494884					5 altered motifs	40kb 3' of AC142293.3
rs1494885					Foxa	40kb 3' of AC142293.3
rs143649097					4 altered motifs	40kb 3' of AC142293.3
rs1494886					Brachyury	40kb 3' of AC142293.3
rs3104124					Osr,VDR	39kb 3' of AC142293.3
rs3104125					Ets,Irf,PU.1	39kb 3' of AC142293.3
rs3104126					Irf,STAT	39kb 3' of AC142293.3
rs3104127					Arid5a,Pou2f2,Pou3f1	38kb 3' of AC142293.3
rs3104128					TCF12	38kb 3' of AC142293.3
rs3104129					GR,NF-I	38kb 3' of AC142293.3
rs3104130					GR,NF-I	38kb 3' of AC142293.3
rs3104133					7 altered motifs	38kb 3' of AC142293.3
rs3104134					8 altered motifs	38kb 3' of AC142293.3
rs1817909					NRSF,Pax-4,Znf143	37kb 3' of AC142293.3
rs1817910					Ets,Gm397	37kb 3' of AC142293.3
rs2367846					HDAC2,STAT,Zfp105	37kb 3' of AC142293.3
rs78461462					HDAC2,Zfp105	37kb 3' of AC142293.3
rs1353294					14 altered motifs	37kb 3' of AC142293.3
rs3113920					8 altered motifs	37kb 3' of AC142293.3
rs3104135					8 altered motifs	37kb 3' of AC142293.3
rs3104136					CEBPB,Foxa,Irx	37kb 3' of AC142293.3
rs3113921		HMEC			9 altered motifs	36kb 3' of AC142293.3
rs3104137		HMEC, NHEK	34 cell types	CEBPB,CJUN,P300	E4BP4,Myc	36kb 3' of AC142293.3

rs3104138	HMEC, NHEK	HAEpiC			36kb 3' of AC142293.3	
rs3104139	HMEC, NHEK	HAEpiC			36kb 3' of AC142293.3	
rs3113922				Nkx2,Nkx3	36kb 3' of AC142293.3	
rs3113923				PLZF,Sox	35kb 3' of AC142293.3	
rs111687808					35kb 3' of AC142293.3	
rs113683669				Zbtb3	35kb 3' of AC142293.3	
rs3104120				Nkx3	35kb 3' of AC142293.3	
rs6857048					34kb 3' of AC142293.3	
rs6840142				DMRT5,ERalpha-a,GR	34kb 3' of AC142293.3	
rs2172797				AhR	34kb 3' of AC142293.3	
rs6835199				8 altered motifs	34kb 3' of AC142293.3	
rs6858801				E2A,Myf	34kb 3' of AC142293.3	
rs6810468				Hand1	34kb 3' of AC142293.3	
rs73826928				RXRA	33kb 3' of AC142293.3	
rs1353293				7 altered motifs	33kb 3' of AC142293.3	
rs1389962				21 altered motifs	32kb 3' of AC142293.3	
rs1494888				19 altered motifs	27kb 3' of AC142293.3	
rs78293098		FibroP		8 altered motifs	24kb 3' of AC142293.3	
rs55994507				14 altered motifs	24kb 3' of AC142293.3	
rs1389965				5 altered motifs	19kb 3' of AC142293.3	
rs1994940	NHLF				18kb 3' of AC142293.3	
rs1994941	NHLF			10 altered motifs	18kb 3' of AC142293.3	
rs72862679					16kb 3' of AC142293.3	
rs7674324	HMEC			11 altered motifs	15kb 3' of AC142293.3	
rs7658108	HMEC				15kb 3' of AC142293.3	
rs12498998	HMEC, HSMM				15kb 3' of AC142293.3	
rs12501733	HMEC			CEBPB,Mef2,p53	15kb 3' of AC142293.3	
rs72864210	5 cell types	26 cell types	GR,JUND		7.4kb 3' of AC142293.3	
rs12506577	HMEC, Huvec, NHEK	HCT-116		Fox	5.1kb 3' of AC142293.3	
rs4694198	HMEC, Huvec, NHEK			Dlx3,Sox	5kb 3' of AC142293.3	

Query SNP: rs13215566 and variants with r^2 >= 0.8 in Asians

rs35953049		Medullo		4 altered motifs	GABRR1	intronic
rs13196063				4 altered motifs	GABRR1	intronic
rs13196423				13 altered motifs	GABRR1	intronic
rs13215017		SK-N-MC		Rad21,YY1	GABRR1	intronic
rs13215029		HRPEpiC,SK-N-MC		5 altered motifs	GABRR1	intronic
rs13215160		HRPEpiC,SK-N-MC		5 altered motifs	GABRR1	intronic
rs13201083				CTCF,NERF1a,RFX5	GABRR1	intronic
**rs13215566**				Gcm1,Pax-6,Zfp128	GABRR1	intronic

Query SNP: rs12206610 and variants with r^2 >= 0.8 in Asians

rs12216245				DMRT3	PDE10A	intronic
rs62426699					PDE10A	intronic
rs62426700				Evi-1,Gfi1	PDE10A	intronic
rs12214904				TLX1::NFIC	PDE10A	intronic
**rs12206610**				Foxd3,Sox,Zfp105	PDE10A	intronic
rs12215013				Foxa	PDE10A	intronic
rs12192968				LUN-1	PDE10A	intronic
rs12206770				ERalpha-a,Spz1,TCF12	PDE10A	intronic
rs62426701				Foxp3	PDE10A	intronic
rs62426702				ATF3,Pou2f2,TCF11::MafG	PDE10A	intronic
rs76154906					PDE10A	intronic
rs76510607				Dobox4,SIX5	PDE10A	intronic
rs76914213				Mrg1::Hoxa9	PDE10A	intronic
rs11751207				5 altered motifs	PDE10A	intronic
rs199547339				12 altered motifs	PDE10A	intronic
rs78291302					PDE10A	intronic
rs11751728				4 altered motifs	PDE10A	intronic
rs12210339		HMVEC-LLy			PDE10A	intronic
rs12190475		4 cell types		4 altered motifs	PDE10A	intronic
rs12191985		4 cell types		GR,HNF4	PDE10A	intronic
rs12210393		4 cell types		4 altered motifs	PDE10A	intronic
rs12192105		Jurkat		EWSR1-FLI1,TATA,p300	PDE10A	intronic

rs12210507			Jurkat,RPTEC		DMRT7,YY1	PDE10A	intronic
rs12212289		HSMM			AP-1,Mef2	PDE10A	intronic
rs12198402		HSMM	HCPEpiC		Pou3f3	PDE10A	intronic
rs12198517		HSMM	Jurkat		4 altered motifs	PDE10A	intronic
rs11752590					PLZF	PDE10A	intronic
rs12195874					NRSF	PDE10A	intronic
rs12195883					Hltf,Pou1f1,Pou5f1	PDE10A	intronic
rs828571					9 altered motifs	PDE10A	intronic
rs12213759					E2F,TATA,YY1	PDE10A	intronic
rs12209263					Pax-4,SIX5,Znf143	PDE10A	intronic
rs12204986					PTF1-beta	PDE10A	intronic
rs12196646					7 altered motifs	PDE10A	intronic
rs12196655					7 altered motifs	PDE10A	intronic
rs12206474					7 altered motifs	PDE10A	intronic
rs12206582			10 cell types		5 altered motifs	PDE10A	intronic
rs12198136			10 cell types		6 altered motifs	PDE10A	intronic
rs12211245			5 cell types		5 altered motifs	PDE10A	intronic
rs142625747						PDE10A	intronic
rs12205255					HNF4,Sox	PDE10A	intronic
rs12200612					5 altered motifs	PDE10A	intronic
rs62424870						PDE10A	intronic
rs60457032					6 altered motifs	PDE10A	intronic
rs57345708			HMVEC-dBl-Neo		NF-I	PDE10A	intronic
rs12212598					PLZF	PDE10A	intronic
rs12206551			Osteobl		Ik-1,Spz1,Zec	PDE10A	intronic
rs12208043						PDE10A	intronic
**Query SNP: rs60843830 and variants with r^2 >= 0.8 in Europeans**							
rs62114494					37 altered motifs	6.4kb 3' of SH3YL1	
rs2126129					7 altered motifs	5.2kb 3' of SH3YL1	
rs62114497		NHEK			MIZF	2.9kb 3' of SH3YL1	
rs6709534					5 altered motifs	395bp 3' of SH3YL1	
rs56350804			PanIsletD		9 altered motifs	169bp 3' of SH3YL1	
rs200781940			PanIsletD		10 altered motifs	167bp 3' of SH3YL1	
rs9213					Ets,SIX5	SH3YL1	3'-UTR
rs3828165					5 altered motifs	SH3YL1	intronic
rs60484953					6 altered motifs	SH3YL1	intronic
rs3791224					4 altered motifs	SH3YL1	intronic
rs3791223					Pou5f1,RBP-Jkappa	SH3YL1	intronic
rs2290911					BRCA1,NF-I,RFX5	SH3YL1	synonymous
rs3791221						SH3YL1	intronic
rs3791220					4 altered motifs	SH3YL1	intronic
rs17713396						SH3YL1	intronic
rs57542652			Th2		Foxp3,NF-AT1	SH3YL1	intronic
rs7601944					11 altered motifs	SH3YL1	intronic
rs2306060					PRDM1	SH3YL1	intronic
rs62114501			Hepatocytes		4 altered motifs	SH3YL1	intronic
rs3838489					19 altered motifs	SH3YL1	intronic
rs6710091					4 altered motifs	SH3YL1	intronic
rs4497901					GR	SH3YL1	intronic
rs17713568					6 altered motifs	SH3YL1	intronic
rs62114505					Evi-1	SH3YL1	intronic
rs55753056					4 altered motifs	SH3YL1	intronic
rs17713729		HepG2			DMRT3,DMRT4,DMRT5	SH3YL1	intronic
rs17713879		K562			Nkx2	SH3YL1	intronic
rs62114538					Foxp1	SH3YL1	intronic
rs55936726		HepG2		SETDB1		SH3YL1	intronic
rs36216559	NHEK	HepG2, HMEC	6 cell types	HEY1,POL2	GR,Nkx2	SH3YL1	intronic
rs7595075	8 cell types	HepG2	19 cell types	5 bound proteins	AP-2,BDP1	SH3YL1	5'-UTR

rs7584915	8 cell types	HepG2	H1-hESC,8988T,Th2	ZEB1,POL2	BDP1,ELF1,HNF4	ACP1	
rs58461606	K562, GM12878	HepG2, NHLF, HMEC			GR	ACP1	intronic
rs56321614	K562	HepG2, GM12878	CMK,HL-60		Irf,TAL1	ACP1	intronic
rs55946380	K562, GM12878	HepG2	HL-60		Cdx,p300	ACP1	intronic
rs62114544	GM12878				Znf143	ACP1	intronic
rs59937473						ACP1	intronic
rs11553746			Th1,Fibrobl,HL-60		4 altered motifs	ACP1	missense
rs62114548			6 cell types	CTCF,RAD21,AP2GAMMA	AP-2,ZEB1	ACP1	intronic
rs7605824					E2F	FAM150B	intronic
rs7566279			Fibrobl			FAM150B	intronic
rs56167434			Fibrobl	POL2	NRSF,Sin3Ak-20,p53	FAM150B	intronic
rs60149603		H1, NHLF			4 altered motifs	FAM150B	intronic
rs17714252		H1, NHLF				FAM150B	intronic
**rs60843830**	H1		WI-38		ERalpha-a,Pbx-1	FAM150B	intronic
rs79154857					CTCFL,TAL1	AC079779.4	
**Query SNP: rs10946507 and variants with r^2 >= 0.8 in Europeans**							
**rs10946507**			7 cell types		GCNF,NF-I,Pou1f1	LINC00340	intronic
rs5874850					Foxp1,HMG-IY,Zfp105	LINC00340	intronic
rs964461					BCL	LINC00340	intronic
rs12216030		GM12878, NHLF		4 bound proteins	PPAR	LINC00340	intronic
**Query SNP: rs8023401 and variants with r^2 >= 0.8 in Europeans**							
rs201102733					6 altered motifs	9.9kb 3' of FBN1	
rs8032307		HSMM, NHLF	15 cell types		CDP,HNF1	7.9kb 3' of FBN1	
rs8032308		HSMM, NHLF	14 cell types		CDP,HNF1	7.9kb 3' of FBN1	
rs12592059					CEBPG,E2F,Pou3f2	4.1kb 3' of FBN1	
rs2899417					GATA,Rad21	399bp 3' of FBN1	
rs13598						FBN1	3'-UTR
**rs8023401**						FBN1	intronic
rs13379564					5 altered motifs	FBN1	intronic
rs1820488		H1				FBN1	intronic
rs8028152						FBN1	intronic
rs9920665		HMEC, NHEK			GR,Maf	FBN1	intronic
rs2042746			15 cell types		Nkx2	FBN1	intronic
rs8029557		NHLF, H1			Zic	FBN1	intronic
rs2278185			5 cell types		6 altered motifs	FBN1	intronic
rs201882828					HNF1,Mef2	FBN1	intronic
rs2466791		Huvec			GR,Sox	FBN1	intronic
rs2017765					STAT,Znf143	FBN1	intronic
rs34539187						FBN1	intronic
rs11855195					4 altered motifs	FBN1	intronic
rs75227249					Mef2,ZBTB33	FBN1	intronic
rs12907167					Pou2f2,Pou3f2	FBN1	intronic
rs17361098					4 altered motifs	FBN1	intronic
rs34215103					Pou2f2	FBN1	intronic
rs16960982					4 altered motifs	FBN1	intronic
rs12917479					8 altered motifs	FBN1	intronic
rs71467652		H1			Hoxa5	FBN1	intronic
rs34070783					5 altered motifs	FBN1	intronic
rs16960997		NHLF			5 altered motifs	FBN1	intronic
rs17458846					HNF1	FBN1	intronic
rs12915497					Ets,TLX1::NFIC,YY1	FBN1	intronic

rs12915240					TLX1::NFIC,YY1	FBN1	intronic
rs12901992						FBN1	intronic
rs12907671					7 altered motifs	FBN1	intronic
rs34837775		HSMM, NHLF			Nkx2	FBN1	intronic
rs12914007		GM12878			CEBPB,p300	FBN1	intronic
rs35464791					4 altered motifs	FBN1	intronic
rs35716640				CTCF	AIRE,Pax-4,Sin3Ak-20	FBN1	intronic
rs11854914		Huvec				FBN1	intronic
rs12909189					6 altered motifs	FBN1	intronic
rs34054358					Hoxa5,Sin3Ak-20	FBN1	intronic
rs17460049						FBN1	intronic
rs17362691				CTCF	GATA,ZEB1,Zfp410	FBN1	intronic
rs2279237						FBN1	intronic
rs1871483					HNF4	FBN1	intronic
**Query SNP: rs16949788 and variants with r^2 >= 0.8 in Europeans**							
**rs16949788**					18 altered motifs	DIS3L	intronic
rs76878359					Maf,NRSF,PLZF	DIS3L	intronic
rs16949793			HeLa-S3		Mrg,Nanog,Sox	DIS3L	intronic
rs9806600			H7-hESC			DIS3L	intronic
rs142910616					Mef2,ZBTB33	DIS3L	intronic
rs8035939					Pbx3	DIS3L	intronic
rs28723485					EBF,Ik-1	DIS3L	intronic
rs11071885					Ets,Irf	DIS3L	synonymous
rs62625678					17 altered motifs	487bp 3' of TIPIN	
rs62625675					Foxo,HDAC2,YY1	TIPIN	3'-UTR
rs62627323					GR,Smad	TIPIN	intronic
rs9806474			Hepatocytes		AP-1,GATA,Smad4	TIPIN	intronic
rs12443313		HepG2			BCL,CHD2,E2F	TIPIN	
rs8042604						TIPIN	
rs12323975					Nanog	TIPIN	
rs16949849		6 cell types	Hepatocytes,Osteob l		6 altered motifs	MAP2K1	intronic
rs80298548					4 altered motifs	MAP2K1	intronic
**Query SNP: rs10880855 and variants with r^2 >= 0.8 in Europeans**							
rs67133230	9 cell types		61 cell types	5 bound proteins	4 altered motifs	ARID2	intronic
rs7138997					4 altered motifs	ARID2	intronic
rs2193749					DMRT1	ARID2	intronic
**rs10880855**					10 altered motifs	ARID2	intronic
rs1468993						ARID2	intronic
rs12320533					4 altered motifs	ARID2	intronic
rs12319077						ARID2	intronic
rs11183201					13 altered motifs	ARID2	intronic
rs201967811					5 altered motifs	ARID2	intronic
rs142543635					6 altered motifs	ARID2	intronic
rs10748432					Cdx,STAT	ARID2	intronic
rs201070908					5 altered motifs	ARID2	intronic
rs79637844					4 altered motifs	ARID2	intronic
rs7132422					10 altered motifs	ARID2	intronic
rs7955891					5 altered motifs	ARID2	intronic
rs2408435						ARID2	intronic
rs35671385					18 altered motifs	ARID2	intronic
rs201994368					5 altered motifs	ARID2	intronic
rs72215781						ARID2	intronic
rs10880859					34 altered motifs	ARID2	intronic
rs1863127					4 altered motifs	ARID2	intronic
rs6582574						ARID2	intronic
rs10880860					7 altered motifs	ARID2	intronic
rs2059404						ARID2	intronic
rs141510569					5 altered motifs	ARID2	intronic
rs7976870					Ik-2,Irf,TCF4	ARID2	intronic
rs247930						ARID2	intronic
rs35117			HMVEC-LBl		Irf,Pax-4,STAT	ARID2	intronic

variant	Promoter histone marks	Enhancer histone marks	DNAse	Proteins bound	Motifs changed	GENCODE genes	dbSNP func annot
rs35115					KAP1	ARID2	intronic

Query SNP: rs10853531 and variants with r^2 >= 0.8 in Europeans

variant	Promoter histone marks	Enhancer histone marks	DNAse	Proteins bound	Motifs changed	GENCODE genes	dbSNP func annot
rs11659892					DMRT5	175kb 3' of SETBP1	intronic
rs11659914					GATA,HDAC2	175kb 3' of SETBP1	intronic
rs16978310		HSMM, NHLF, NHEK	6 cell types		YY1	175kb 3' of SETBP1	intronic
rs7235910		NHEK, HMEC	BJ	GATA3	Egr-1,Hbp1	176kb 3' of SETBP1	intronic
**rs10853531**					CACD,NRSF,Pax-4	176kb 3' of SETBP1	intronic

Supplementary Note 1 Study Description

ALIENOR

The Alienor study is a population-based study in residents of Bordeaux, France[25]. The 963 participants, aged 73 years or more, were recruited from an ongoing population-based study (3C Study) [26]. They underwent an ophthalmological examination, including a recording of ophthalmological history, measures of visual acuity, refraction, two 45° non mydriatic colour retinal photographs (one centred on the macula, the other centred on the optic disc), measures of intraocular pressure and central corneal thickness and break-up time test. Refraction was measured first using autorefractometer (Speedy K, Luneau, France) and secondly by measuring subjective measurement, which was used in the analysis. This research followed the tenets of the Declaration of Helsinki. Participants gave written consent for the participation in the study. The design of this study has been approved by the Ethical Committee of Bordeaux (Comité de Protection des Personnes Sud-Ouest et Outre-Mer III) in May 2006.

After exclusion of subjects operated for cataract and other eye procedures and diseases that could alter refraction, 618 subjects were available, among which 529 were genotyped at the French national centre for genotyping (CNG) using Illumina Human 610-Quad BeadChip. Among them, 509 individuals had good genotype QC (individuals of European ancestry, unrelated with other individuals, without discrepancy between clinical and genetic gender and with missingness < 5%) and had imputation data. In addition, 2 subjects had missing education data, leaving 507 subjects in the statistical analysis. Imputation was performed in two steps: pre-phasing with SHAPEIT2, followed by imputation with IMPUTE2 using 1000 Genomes(March 2012, MACGT1) as reference panel. SNPs were used in the imputation process if call rate > 98%, HWE p-value > 1×10^{-6}, MAF> 1%. Analysis was performed using Quicktest, with adjustment on age, gender, education, PC1 and PC2 and modelling of interaction between SNP and education, using robust variance estimates. No SNP exclusion was applied on imputed SNPs.

Avon Longitudinal Study of Parents and Children (ALSPAC)

Details of ALSPAC cohorts have been published previously[8; 9]. The research adhered to the tenets of the Declaration of Helsinki. Ethical approval for the study was obtained from the ALSPAC Law and Ethics committee and three local research ethics committees. Pregnant women with an expected date of delivery between 1st April 1991 and 31st December 1992, resident in the former Avon health authority area in Southwest England, were eligible to participate in this birth cohort study. 13,761 women were recruited. Data collection has been via various methods including self-completion questionnaires sent to the mother, to her partner and after age 5 to the child; direct assessments and interviews in a research clinic. As well as investigating the health and well-being of the children in the birth cohort, the health of the mothers is also an important area of investigation. For mothers, DNA was extracted from blood samples collected as part of routine antenatal care, during attendance at ALSPAC research clinics, or from immortalized lymphoblastoid cell lines, for a total of 10,321 of the mothers. Non-cycloplegic autorefraction (Canon R50 instrument) was performed opportunistically when mothers accompanied their child to a research clinic visit, and/or by a researcher visiting their optician to obtain their spectacle prescription. The design of this study has been approved by the ALSPAC Ethics Committee and National Health Service Research Ethics Committee.

Non-cycloplegic autorefraction data was used in preference to subjective refraction data when available. DNA samples were available for 11,343 children, prepared from either blood samples or lymphoblastoid-transformed cell lines. Non-cycloplegic autorefraction (Canon R50 instrument) was performed during attendance at an ALSPAC research clinic visit when the children were approximately 15 years old. Genotyping was performed using Illumina 660 W-quad (mothers) or Illumina HumanHap 550 (children) bead arrays. Samples that did not cluster with HapMap CEU individuals on IBS plots, with excessive missingness (>5%), minimal or excessive autosomal heterozygosity, cryptic relatedness (>10% IBD) or with a sex-mismatch were excluded. SNPs with call

rate <95%, minor allele frequency <1%, or Hardy-Weinberg P value < 10^{-7} were excluded. Genotypes were available for 8340 mothers and 8365 children. Imputation was carried out separately for Mothers and Children. For mothers, individual chromosomes were pre-phased with ShapeIt v2 using the b37 genetic map, and imputation was performed with minimac-omp using the GIANT phase1 release v3 (2010-11-23) 1000 Genomes reference panel. For children, phasing was carried out using MACH and imputation with minimac, against the same reference panel. Genotype and phenotype data were available for 1865 mothers and 3792 children. SNP x education interaction was performed using Probabel for mothers. In children, tests for SNP main effect and SNP x near work interaction were carried out using R for 3 SNPs that showed evidence of SNP x Education interaction effects in the meta-analysis of Asian adults.

AREDS

The Age-Related Eye Disease Study (AREDS) was initially designed as a long-term multicenter, prospective study of the clinical course of age-related macular degeneration (AMD) and age-related cataract [27; 28]. In addition to collecting natural history data, AREDS included a randomized clinical trial of high-dose vitamin and mineral supplements for AMD and a clinical trial of high-dose vitamin supplements for cataract [27-29]. Prior to study initiation, the protocol was approved by Institutional Review Boards for each of the 11 clinical centers in 1992: Eye Center at Memorial Albany, New York; Associated Retinal Consultants, Michigan; Devers Eye Institute, Oregon; Emory University, Georgia; Massachusetts Eye and Ear Infirmary, Massachusetts; National Eye Insititute, Maryland; Univeristy of Pittsburgh Eye and Ear Institute, Pennsylvania; Ingalls Memorial Hospital, Illinois; Johns Hopkins Medical Institutions, Maryland; Elman Retina Group, Maryland; University of Wisconsin, Wisconsin. Written informed consent was obtained from all participants before enrollment in accordance with the Declaration of Helsinki. AREDS participants were 55 to 80 years of age at enrollment and had to be free of any illness or condition that would make long-term follow-up or compliance with study medications unlikely or difficult. On the basis of fundus photographs graded by a central reading center, best-corrected visual acuity and ophthalmologic evaluations, 4,757 participants were enrolled in one of several AMD categories, including persons with no AMD (control group). Visual acuity measurement of all participants was performed with the standard procedure developed for the Early Treatment of Diabetic Retinopathy Study (ETDRS). A refraction measurement was performed for participants at the randomization visit and each annual visit. For those who experience a decrease of 10 letters from baseline visual acuity, refractions were also conducted at the non-annual visits. Blood samples were collected at baseline and longitudinally, and cell lines were established. DNA was extracted from cell lines according to standard protocols when the initial DNA supply has been depleted.

For the current analysis, 1865 participants were included from the AREDS 1c population. Refractive error which was measured by a refraction protocol at baseline enrollment into the AREDS study [27-30] was utilized for the definition of astigmatism. For AREDS 1c, genotyping of SNPs was performed using the Illumina HumanOmni2.5-4v1_B chip array and a genome-wide association study of astigmatism using the Illumina 2.5M chip was performed using a subset of the control group from the original AREDS study. These control individuals are all Caucasians, who do not have age-related macular degeneration (AMD) and were further screened to also exclude individuals with cataracts, retinitis pigmentosa or other retinal degenerations, color blindness, other congenital eye problems, LASIK, artificial lenses, and other eye surgery. For all studies, samples with low call rate (<98%), with low mean confidence scores over all non-missing genotypes, with chromosome anomalies, or with sex-mismatch were excluded. No samples exhibited excess heterozygosity rates (1.5 interquartile ranges above or below the upper/lower quartile ranges). Cryptic relatedness was detected by estimating IBD sharing and kinship coefficients among all possible pairs and one member of each pair exhibiting a first cousin or closer relationship was dropped from the analysis. SNPs were dropped from the analysis if they exhibited more than 1 blind duplicate error, more than 1 HapMap control error or more than 1 error in HapMap control trios, a genotype call rate < 99%, minor allele frequency < 0.01, or Hardy-Weinberg P-value < 1×10^{-4}. Tests for batch effects were not significant. No sex-specific differences in allelic frequency ($P > 0.2$) or heterozygosity ($P > 0.3$)

were detected. Imputation was performed with the IMPUTE version 2 software (imputed to plus strand of NCBI build 37, 1000 Genomes worldwide reference panel of 1,092 samples from phase I integrated variant set (v3, release March 2012)). For each imputed SNP, info, a measure of imputation quality was calculated. Info typically ranges between 0 and 1, 1 indicating no uncertainty in imputed genotypes. Quicktest was used for analyses including age, sex and the first two principal components (to adjust for population stratification) as covariates. Genotype data from AREDS 1c are publicly available through the database of Genotype and Phenotype under the name of either the MMAP study or the AREDS study.

BATS

The Brisbane Adolescent Twins Study (BATS) is a part of the Australian Twin Eye Study[31]. Ethical approval was obtained from the QIMR Berghofer Medical Research Institute-Human Research Ethics Committee. In all subjects post-cycloplegic (following instillation of tropicamide 1%) refraction for both eyes was measured using a Humphrey-598 automatic refractor (Carl Zeiss Meditec, Inc., Miami, Florida, USA). These measurements were used to determine the spherical equivalence trait analysed here. Education data in BATS were collected as part of the 19UP study, through either telephone interviews or online questionnaires. We restricted the analyses to those of 20-year-old or above.

DNA was extracted from blood leucocytes according to standard procedures. The Australian cohorts were genotyped on the Illumina Human Hap610 Quad array. SNPs with a genotype success rate of 0.95 or above was required for inclusion of the SNP into further steps of the analysis. Only SNPs in Hardy-Weinberg equilibrium were processed: the HWE inclusion threshold was P>10x10-6. The minimum minor allele frequency required for inclusion of individual SNPs was 0.01. Ancestral outliers were defined as having the first two principal components more than six standard deviations from the mean values of HapMap European samples, and therefore were subsequently excluded from the analyses. Imputation was performed against version 3 of the November 23, 2010 version of the publicly released 1000 Genomes Project genotyping, using MACH for phasing and minimac for imputation We used the two-step score test for this analysis, with the first step fitting a mixed model for spherical equivalence adjusted for age, sex and kinship matrix using GenABLE, and the second step using the GWFGLS function in MixABEL which fits a linear model for the residual of spherical equivalence from the first step and tests the main SNP effect and the SNP x education interaction term.

Blue Mountains Eye Study (BMES)

The Blue Mountains Eye Study (BMES) is a population-based cohort of a predominantly white population in west of Sydney, Australia. At baseline (1992-94), 3,654 permanent residents aged 49 years or older participated (participation rate of 82.4%[9]. During 1997-99 (BMES II A), 2,335 participants (75.1% of survivors) returned for examinations after 5 years. During 1999-2000, 1,174 (85.2%) new participants took part in an Extension Study of the BMES (BMES IIB). BMES cross-section II thus includes BMES IIA (66.5%) and BMES IIB (33.5%) participants (n=3,509)[32]. From the BMES cross section II who had blood samples collected, DNA was extracted for 3,189 (90.1 %) participants. Over 98% of BMES participants were European ancestry. All BMES examinations were approved by the Human Research Ethics Committees of the Western Sydney Area Health Service and University of Sydney.Signed informed consent was obtained from participants at each examination. Participants of the BMES cross section II who had DNA available in early 2009 (n=2983) were genotyped using the Illumina Human 670-Quadv1 custom genotyping array at the Wellcome Trust Sanger Institute, Cambridge as part of WTCCC2, and 2,761 had genotyping data available. Following exclusion through GWAS and DNA quality control and phenotype exclusion criteria resulted in genotyping data being available for 1,896 individuals. Imputation was performed to HapMap (NCBI Build 36.1) using MACH (V 1.0.16; autosomes only). Imputed SNPs were excluded from the analysis when failing one or more of the following QC filters: 1) prop info ≥ 0.5 (a software-specific statistic from IMPUTE); 2) Hardy-Weinberg P-value < 1×10^{-6}. We did not filter the SNPs with MAF < 0.01 from the imputed SNPs so that rare SNPs were included for association assessment.

CROATIA-Korčula Study

The CROATIA-Korčula study, Croatia, is a population-based, cross-sectional study that includes a total of 969 adult examinees, aged 18-98 (mean=56.3), from the Dalmatian island of *Korčula* and most (N=930) underwent a complete eye examination [33]. The study received approval from Ethics Committee of the Medical School, University of Split and NHS Lothian Board in Scotland and Croatia and followed the tenets of the Declaration of Helsinki. Non-cycloplegic autorefraction was measured on each eye using a NIDEK Ark30 hand-held autorefractometer. Measures on eyes with a history of trauma, intra-ocular surgery, LASIK operations or keratoconus were removed. Analysis was performed as per analysis plan, excluding individuals with a cylinder power >= 5D in either eye and individuals with difference in cylinder power between right and left eyes beyond 4 standard deviations from the mean, and for over 25 year-old only as there were too few individuals in this study who were under 25 years of age. Genotypes were generated using a dense Illumina SNP arrays, Illumina CNV370v1 and CNV370-Quadv3, following the manufacturer's standard recommendations. Genotypes were determined using the Illumina BeadStudio software. Samples with a call rate below 97% , potentially mixed samples with excess autosomal heterozygosity or gender discrepancy (based on the sex chromosomes genotypes), and ethnic outliers (based on principal components analysis of genotypic data), were excluded from the analysis using the quality control algorithm implemented in the R package GenABEL. After exclusion of SNP with MAF < 0.01, call rate < 98% and HWE deviation $p < 10^{-6}$, samples were pre-phased using shapeit v2[34]. Imputation was carried out using impute v2[35] and the 1,000 genomes All ancestries phase1 integrated v3 reference panel. The impute2mach GENABEL function was used to convert the impute2 outputs to the MACH format that is used in the ABEL suite (http://www.genabel.org/packages) and the regression analyses of Spherical Equivalent Refraction adjusted for age and sex on SNP allele dose, education and interaction between SNP and education performed using the MixABEL package. The variance covariance matrix used in MixABEL to account for relatedness between individuals was generated using the polygenic functions of the GenABEL package. After phenotypic and genotypic quality control steps, 807 individuals were analysed.

CROATIA-Split Study

The CROATIA-Split study, Croatia, is a population-based, cross-sectional study in the Dalmatian City of Split that includes 1000 examinees aged 18-95. The study received approval from Ethics Committee of the Medical School, University of Split and NHS Lothian Board in Scotland and Croatia and followed the tenets of the Declaration of Helsinki. Individuals were genotyped with either the 370CNV-Quadv3 (n=500) or the Illumina OmniExpress Exome-8v1_A beadchips (n=500). Alleles were called in BeadStudio/GenomeStudio using Illumina cluster files. Subjects were excluded if they fulfilled any of the following criteria: genotypic call rate <97%, mismatch between reported and genotypic sex, unexpectedly low genomic sharing with first degree relatives, excess autosomal heterozygosity, or outliers identified by IBS clustering analysis. We excluded SNPs on the basis of minor allele frequency (<0.01/monomorphism), HWE ($P < 10^{-6}$), call rate (<97%). The samples genotyped with the denser array (Illumina OmniExpress Exome) were first prephased and imputed as described for the CROATIA-Korcula study and the output of this imputation used as a secondary panel to complement the 1,000 genomes. All ancestries phase1 integrated v3 reference panel for the imputation of the samples genotyped on the less dense array. Imputations for the two halves of the study were then combined to form a combined panel of ~37.5m SNPs. Genome-wide scan for association was performed as described in the CROATIA-Korcula Study.

Diabetes Control and Complications Trial (DCCT)

DCCT (1982-1993) was a multi-center randomized clinical trial to compare the effectiveness of intensive (≥3 daily insulin injections or insulin pump) and conventional (<3 daily insulin injections) diabetic treatments at the time in preventing development and progression of microvascular complications of type 1 diabetes[36]. Ethical approval was obtained from the Research Ethics Board of The Hospital for Sick Children. Subjective refraction was measured following the standard protocols using a letter chart at 10 to 20 feet, at baseline visit and annually

thereafter during DCCT. Refraction measurement was attempted at 1 meter for the subjects with poor visual acuity. In these cases the 4 meter refraction was estimated by subtracting +0.75 sphere from the 1 m measurement. In the current study the last available measurement for each individual was used. Genotyping was done using Illumina Human1M BeadChip assay. Individuals showing gender mismatch with typed X-linked markers, call rate <0.95, genotyping mismatch with an earlier study, high autosomal heterozygosity or cryptic relatedness were excluded from the analysis. Analysis was restricted to individuals who were self-identified as "white" and of 20 years or older. Ethnically admixed subjects, identified using population genetic approaches, were also excluded from further analysis. Details of genotyping quality control procedures are presented elsewhere[37]. Genotyped of untyped markers where imputed using 1000 Genomes Phase I integrated haplotypes as reference in IMPUTE v2.3.0.

Estonian Genome Center, University of Tartu (EGCUT)

The Estonian cohort is from the population-based biobank of the Estonian Genome Project of University of Tartu (EGCUT). The project was approved by the Research Ethics Committee of the University of Tartu and conducted according to the Estonian Gene Research Act. All participants have signed the broad informed consent (http://www.biobank.ee[14]). The current cohort size is over 51,515, from 18 years of age and up, which reflects closely the age distribution in the adult Estonian population. Subjects are recruited by the general practitioners (GP) and physicians in the hospitals were randomly selected from individuals visiting GP offices or hospitals. Each participant filled out a Computer Assisted Personal interview during 1-2 hours at a doctor's office, including personal data (place of birth, place(s) of living, nationality etc.), genealogical data (family history, three generations), educational and occupational history and lifestyle data (physical activity, dietary habits, smoking, alcohol consumption, women's health, quality of life). Anthropometric and physiological measurements were also taken. All diseases are defined according to the ICD10 coding. All the samples are genotyped with Illumina HumanCNV370 or HumanOmniExpress according to the Illumina protocol and the samples were assigned to discovery and replication by the availability on the time of analyses. Data quality control was performed with PLINK (http://pngu.mgh.harvard.edu/purcell/plink) (SNP call rate>98%; sample call rate >95%; MAF >0.01; HWE P $>10^{-6}$; cryptic relatedness). SHAPEIT v1 was used for phasing and IMPUTE v2.2.2. for imputation (1000 Genome Phase 1 integrated variant set, Amr 2012). GWAS GxE analysis was carried out with Quicktest.

EPIC-Norfolk Eye Study (EPIC)

The European Prospective Investigation into Cancer (EPIC) study is a pan-European prospective cohort study designed to investigate the aetiology of major chronic diseases[38]. EPIC-Norfolk , one of the UK arms of EPIC, recruited and examined 25,639 participants aged 40-79 years between 1993 and 1997 for the baseline examination[39]. Recruitment was via general practices in the city of Norwich and the surrounding small towns and rural areas, and methods have been described in detail previously [40]. Since virtually all residents in the UK are registered with a general practitioner through the National Health Service, general practice lists serve as population registers. Ophthalmic assessment formed part of the third health examination and this has been termed the EPIC-Norfolk Eye Study[41]. In total, 8,623 participants were seen for the ophthalmic examination, between 2004 and 2011. Refractive error was measured using a Humphrey Auto-Refractor 500 (Humphrey Instruments, San Leandro, California, USA). Educational level was recorded and classified into four groups according to the highest qualification achieved (Less than O level / O Level / A level / Degree). For the purposes of the current study, educational attainment was dichotomised into lower (Less than O level / O Level) or higher (A level / Degree). Genotyping was undertaken using the Affymetrix GeneChip Human Mapping 500K Array Set. Data were pre-phased with SHAPEIT version 2 and imputed to the March 2012 build of the 1000 Genomes project using IMPUTE version 2.2.2. The EPIC-Norfolk Eye Study was carried out following the principles of the Declaration of Helsinki and the Research Governance Framework for Health and Social Care and was approved by the Norfolk Local Research Ethics Committee (05/Q0101/191) and East Norfolk & Waveney NHS Research Governance Committee (2005EC07L). All participants gave written, informed consent.

Erasmus Rucphen Family Study (ERF)

The Erasmus Rucphen Family (ERF) Study is a family-based cohort in a genetically isolated population in the southwest of the Netherlands with over 3,000 participants aged between 18 and 86 years. Cross-sectional examination took place between 2002 and 2005. The rationale and study design of this study have been described elsewhere [42; 43]. Cross-sectional examination took place between 2002 and 2005, including a non-dilated automated measurement of refractive error using a Topcon RM-A2000 autorefractor. All measurements in these studies were conducted after the Medical Ethics Committee of the Erasmus University had approved the study protocols and all participants had given a written informed consent in accordance with the Declaration of Helsinki.

DNA was genotyped on one of four different platforms (Illumina 6k, Illumina 318K, Illumina 370K and Affymetrix 250K). Samples with low call rate (<97.5%), with excess autosomal heterozygosity (>0.336), or with sex-mismatch were excluded, as were outliers identified by the identity-by-state clustering analysis (outliers were defined as being >3 s.d. from population mean or having identity-by-state probabilities >97%). A set of genotyped input SNPs with call rate >98%, with minor allele frequency >0.01, and with Hardy-Weinberg P value $>10^{-6}$ was used for imputation. We used Minimac to impute to 1000G (phase 1, March 2012). For each imputed SNP, a reliability of imputation was estimated as the ratio of the empirically observed dosage variance to the expected binomial dosage variance (O/E ratio). GWAS GxE analyses were performed using the MixABEL package and adjusted for family structure in the first step of two-staged modelling.

Framingham Eye Study

The Framingham Eye Study [44] (FES) was nested within the Framingham Heart Study (FHS, http://www.framinghamheartstudy.org), which began its first round of extensive physical examinations in 1948 by recruiting 5,209 men and women from the town of Framingham, MA, USA. Surviving participants from the original cohort returned for biennial exams, which continue to the present. A total of 2675 FHS participants were also examined as part of the FES between 1973 and 1975. The FES was designed to evaluate ocular characteristics of examinees such as: senile cataract; age-related macular disease; glaucoma; and retinopathy. Between 1989 and 1991, 1603 offspring of original cohort participants also received ocular examinations [45]. The analyses in the current study are limited to 1532 participants (43.9% men) from both the original and the offspring cohorts for whom both phenotype and genotype data were available. Most individuals in this analysis set are unrelated but a small number of related pairs remain. All data--including refractive error, demographics and genotypes--were retrieved from the database of Genotypes and Phenotypes (dbGaP, http://www.ncbi.nlm.nih.gov/gap) after approval for controlled access to individual-level data. All study protocols are in compliance with the World Medical Association Declaration of Helsinki. Since 1971, written consent has been obtained from participants before each examination. The design of this study was approved by Johns Hopkins Bloomberg School of Public Health Institutional Review Board (FWA#0000287). The research protocols of the Framingham Heart Study are reviewed annually by the Institutional Review Board of the Boston University Medical Center and by the Observational Studies Monitoring Board of the National Heart, Lung and Blood Institute.

Genotyping was conducted as part of the NHLBI Framingham SNP Health Association Resource (SHARe). This sub-study contains genotype data for approximately 550000 SNPs (Affymetrix 500K mapping arrays [Mapping250k_Nsp and Mapping250K_Sty] plus Affymetrix 50K supplemental human gene-focused array) in over 9200 FHS participants (1497 of whom were used in this analysis). Samples were chosen based on pedigree information and genotyping quality; samples with a genotypic call rate below 95% were not chosen for analysis. The mean call rate for analyzed samples was 99.2% (SD=0.4%). Genotype data cleaning was carried-out in several steps. The final marker list contained 436,494 high-quality SNPs with a minor-allele frequency >= 0.01, a

Mendelian error rate below 2% across all pedigrees, a genotype call rate above 95%, and whose distribution was consistent with Hardy-Weinberg expectations ($P>10^{-4}$). Genotype imputation to the HapMap-II reference panel (CEU population release 22, NCBI build 36) was carried out in a two-step process using the Markov Chain Haplotyping (MACH version 1.0.16.a) software. First, crossover and error-rate maps were built using 400 unrelated individuals (200 male and 200 female) sampled from FHS subjects. Second, genotype imputations of 1000 Genomes haplotypes reference panel were carried out on the entire FHS dataset using parameters estimated from step 1. Statistical analyses were conducted with the R statistical software (version 2.7) and the GenABEL (version 1.7-2) and MixABEL (version 0.1-1) packages for linear mixed model association analyses. Linear mixed models included age, sex, the first two eigenvectors from principal components analyses of genotype data, a binary coding of education (0, 1), and the additively-coded SNP dosage (0 to 2). For the original cohort, years of schooling was not reported but an ordinal variable ranging from 0 (no schooling) to 8 (post-graduate) was collected. An interaction (G x E) term was generated as the product of additively-coded SNPs with the binary education variable. The kinship matrix was estimated empirically from the data and included as a random effect in the statistical model.

Finnish Twin Study on Aging (FITSA)

Finnish Twin Study on Aging (FITSA) is a study of genetic and environmental effects on the disablement process in older female twins. The FITSA participants were 103 MZ and 114 DZ Finnish twin pairs (424 individuals, all Caucasian women) aged 63-76 years who took part in multiple laboratory examination in 2000, 2003 and responded in questionnaires in 2011. Before the examinations, the subjects provided a written informed consent according to the Declaration of Helsinki. The study protocol was approved by the ethics committee of the Central Hospital District of Central Finland.

DNA was extracted from EDTA-anticoagulated whole blood according to standard procedures. The genotyping was carried out with Illumina HumanCoreExome chip. The genotyping quality control thresholds included minor allele frequency >0.01, success rate by marker >0.95, success rate by individual >0.95, and HWE $P>10^{-6}$. The imputation was performed with SHAPEIT2 and IMPUTE2 with 1000 Genomes haplotypes reference panel (Phase I integrated variant set release in NCBI build 37 (hg19) coordinates). The Quicktest version 0.95 was utilized in GxE regression analysis.

Gutenberg Health Study (GHS1, GHS2)

The Gutenberg Health Study (GHS) is a population-based, prospective, observational cohort study in the Rhine-Main Region in midwestern Germany with a total of 15,010 participants and follow-up after five years. The study sample is recruited from subjects aged between 35 and 74 years at the time of the exam. The sample was drawn randomly from local governmental registry offices and stratified by gender, residence (urban and rural) and decade of age. Exclusion criteria were insufficient knowledge of the German language to understand explanations and instructions, and physical or psychic inability to participate in the examinations in the study center. Individuals were invited for a 5-hour baseline-examination to the study center where clinical examinations and collection of blood samples were performed. The interdisciplinary study design comprises an ophthalmological examination, general and especially cardiovascular examinations, psychosomatic evaluation, laboratory tests, and biobanking for proteomic and genetic analyses. All participants underwent an ophthalmological investigation of 25 minutes' duration taking place between 11:00 a.m. and 8:00 p.m. This examination was based on standard operating procedures and included a medical history of eye diseases, autorefraction and visual acuity testing (Humphrey® Automated Refractor/Keratometer (HARK) 599™, Carl Zeiss Meditec AG, Jena, Germany), visual field screening using frequency doubling technology (Humphrey® Matrix Perimeter, Carl Zeiss Meditec AG, Jena, Germany), central corneal thickness and keratometry measurement (Scheimpflug imaging with the Pachycam™, Oculus, Wetzlar, Germany), IOP measurement with a non-contact tonometer (Nidek NT-2000™, Nidek Co., Japan), slitlamp biomicroscopy with undilated pupils (Haag-Streit BM

900®, Bern, Switzerland) and non-mydriatic fundus photography (Visucam PRO NM,™, Carl Zeiss Meditec AG, Jena, Germany), all administered by an ophthalmologist. The study was approved by the Local Ethics Committee of Rhineland-Palatinate, Germany (reference no. 837.020.07). According to the tenets of the Declaration of Helsinki, written informed consent was obtained from all participants prior to entering the study.

Within GHS, DNA was extracted from buffy-coats from EDTA blood samples as described in Zeller *et al*.[46]. Genetic analysis was conducted in the first 5,000 study participants. For these, 3,463 individuals were genotyped in 2008 (GHS1) and further 1,439 individuals in 2009 (GHS2). Genotyping was performed for GHS1 and GHS2 using the Affymetrix Genome-Wide Human SNP Array 6.0 (http://www.affymetrix.com), as described by the Affymetrix user manual. Genotypes were called using the Affymetrix Birdseed-V2 calling algorithm. Individuals with low genotyping call rate, a too high level of heterozygosity (hetFDR>0.01)), with sex-mismatches, and with Non-European ancestry were excluded. After applying standard quality criteria (minor allele frequency >1%, genotype call rate >98% and P-value of deviation from Hardy-Weinberg equilibrium of >0.0001), 689,634 SNPs in 2996 individuals from GHS1 and 701,418 SNPs in 1,179 individuals from GHS2 remained for analysis (total 4175). Imputation of missing genotypes was performed using the software MACH (v1.0.18.c) and minimac (release 2012-03-14) with the reference panel 1000G Phase I Integrated Release Version 2 Haplotypes (2010-11 data freeze, 2012-02-14 haplotypes) for each cohort separately. SNP x Education interaction analyses were performed using ProbABEL (v0.4.1) with age and sex included in the model as covariates.

KORA

KORA ("Kooperative Gesundheitsforschung in der Region Augsburg" which translates as “Cooperative Health Research in the Region of Augsburg”) is a population based study of adults randomly selected from 430,000 inhabitants living in Augsburg and 16 surrounding counties in Germany 25-28[47-50].. The collection was done in 4 separate groups from 1984-2001 (S1-S4). All survey participants are residents of German nationality identified through the registration office. In the KORA S3 and S4 studies 4,856 and 4,261 subjects have been examined implying response rates of 75% and 67%, respectively. 3,006 subjects participated in a 10-year follow-up examination of S3 in 2004/05 (KORA F3), and 3080 of S4 in 2006/2008 (KORA F4). The age range of the participants was 25 to 74 years at recruitment. The study was approved by ‘Ethik-Kommission der Bayerischen Landesärztekammer’. Written informed consent was obtained from all participants before enrollment in accordance with the Declaration of Helsinki.

Genome-wide genotyping using the Illumina 2.5M chip or the Illumina Omni Express chip was performed on a subset of individuals from the S3/F3. Samples with low call rate (<98%), sex-mismatch, exhibited excess heterozygosity rates or evidence for non-Caucasian ancestry were excluded. SNPs were excluded before imputation if they had a low a genotype call rate (<0.98), low minor allele frequency (<0.01) or Hardy-Weinberg P-value $<10^{-6}$. Phasing and imputation was performed with SHAPEIT v2 and IMPUTE2 v2.3.0 using the 1000g phase 1 integated reference panel. Eyeglass prescriptions were measured in addition to an evaluation using the Nikon Retinomax and subjects with age-related macular degeneration, cataracts, retinitis pigmentosa, color blindness, other congenital eye problems, LASIK, artificial lenses, and other eye surgery were excluded. GxE analyses were done with QUICKTEST version 0.95.The genomic control inflation factor was 1.016 (after filtering SNPs for MAF >1%, imputation quality info >0.3).

Ogliastra Genetic Park, Talana study (OGP Talana)

A cross-sectional ophthalmic study was performed in Talana, Perdasdefogu and Urzulei within the Ogliastra Project, a large epidemiological survey conducted in a geographically, culturally and genetically isolated population living in an eastern-central region of Sardinia[51]. The study protocol has been approved by the Institutional Review Board of the Italian Ministry of Education, Universities and Research. In Talana the study was carried out between October 2001 and October 2002 and adhered to the tenets of the declaration of

Helsinki. Talana is an Ogliastran village situated at an altitude of 700 m above sea level in one of the most secluded areas of Sardinia; it has about 1200 inhabitants and, importantly, archival records are available from 1589 and genealogical trees have been reconstructed from 1640. 789 volunteers gave their written informed consent and were invited to the local medical centre, which was equipped with a complete set of ophthalmic instruments for this survey. All participants underwent a complete eye examination conducted according to a standardized protocol that included visual acuity measurement with Snellen charts at a distance of 5 m, autorefraction (RK-8100 Topcon, Tokyo, Japan) assessing sphere, cylinder and axis, slit lamp biomicroscopy (Model BQ900, Haag-Streit, Bern, Switzerland), contact tonometry and colour fundus photography (TRC-50IA,Topcon) and non-contact optical biometry (IOLMaster,Carl Zeiss, Italy) and Optical coherence tomography (OCT). Whole blood was obtained from all consenting family members of Talana village for DNA extraction.

Genotyping was carried out using the Affymetrix 500k chips using standard protocols. SNPs quality control was performed using the GenABEL software package in R. Samples with overall SNP call rate < 95%, showing excess of heterozygosity, or being classified as outliers by allelic identity-by-state (IBS) clustering analysis, were excluded. After exclusion of SNPs with minor allele frequency < 0.05, Hardy-Weinberg P value >10^{-4} and call rate < 95%, data were pre-phased with Shapeit and imputed with Impute2 Using the GIANT phase 1 release v3 1000 Genome reference panel. Genome-wide GxE association analysis was performed using MixABEL.

Orkney Complex Disease Study (ORCADES)

The Orkney Complex Disease Study (ORCADES) is a population-based, cross-sectional study in the Scottish archipelago of Orkney, including 1,285 individuals with eye measurements. The study received approval from the Orkney and North of Scotland Local Research Ethics Committees in Scotland and followed the tenets of the Declaration of Helsinki. Autorefractive measurements were obtained using a Kowa KW 2000 autorefractometer. Measures on eyes with a history of trauma, intra-ocular surgery, LASIK operations or keratoconus were removed. Analysis was performed as per analysis plan excluding individuals with a cylinder power >= 5D in either eye and individuals with difference in cylinder power between right and left eyes beyond 4 standard deviations from the mean, and for over 25 year-old only as under 25 year were too few.

Individuals were genotyped with either the Illumina HumanHap300v2 or 370CNV-Quad beadchips (n=890) or the Illumina Omni1 (n=304) or Illumina OmniExpress beadchips (n=1073). Alleles were called in BeadStudio/GenomeStudio (Hap300/Omni) using Illumina cluster files. Subjects were excluded if they fulfilled any of the following criteria: genotypic call rate <98%, mismatch between reported and genotypic sex, unexpectedly low genomic sharing with first degree relatives, excess autosomal heterozygosity, or outliers identified by IBS clustering analysis. We excluded SNPs on the basis of minor allele frequency (<0.01/monomorphism), HWE (P<10^{-6}), call rate (<97%). Given the very high overlap in SNPs between the two Omni chips, the intersection of QC'd SNPs was used to impute and phase individuals genotyped on the Omni arrays together, whilst the Hap300 individuals were phased and imputed separately. Samples were phased using shapeit v2. Imputation was carried out using impute2 and the 1,000 genomes All ancestries phase1 integrated v3 reference panel, with a secondary reference panel of local exome sequences, sequenced using the Agilent SureSelect All Exon Kit v2.0 and Illumina 100 bp paired end reads (average 30x depth), derived from 90 ORCADES subjects chosen to optimally represent the haplotypes present. Imputations for the Hap300 and Omni subjects were then combined to form a combined panel of 37.5 M SNPs for 2222 subjects[52]. The impute2mach GENABEL function was used to convert the impute2 outputs to the MACH format that is used in the ABEL suite (http://www.genabel.org/packages) and the regression analyses of Spherical Equivalent Refraction adjusted for age and sex on SNP allele dose, education and interaction between SNP and education performed using the MixABEL package. The variance covariance matrix used in MixABEL to account for relatedness was generated using the polygenic functions of the GenABEL package.

RAINE Eye Health Study (RAINE)

The Raine Eye Health Study (REHS) was conceived to determine the prevalence of and risk factors for eye disease in young adults, and to characterize ocular biometric parameters in a young adult cohort [53]. The Western Australian Pregnancy Cohort (Raine) Study originated as a randomized-controlled trial of 2900 women recruited from the state's largest maternity hospital. The design of study has been approved by the Human Research Ethics Committee, University of Western Australia. Their offspring (N=2868) have been followed at birth, ages 1, 2, 3, 5, 8, 10, 14, 17 and 20 years of age in a prospective cohort study. DNA was collected from participants for genome-wide association studies and genotyping was performed using Illumina 660 Quad Array. Any pair of individuals who were related with a $\pi > 0.1875$ (in between second and third degree relatives – e.g. between half-sibs and cousins) was investigated, and the individual with the higher proportion of missing data was excluded from the 'clean' dataset (68 individuals excluded). Individuals who had low genotyping success (i.e. missing data) were excluded from the 'clean' dataset – a threshold of absent data > 3% was used for exclusion (16 individuals excluded). Additionally, if they had high levels of heterozygosity then they were also excluded (heterozygosity < 0.30 excluded 3 individuals). SNPs which did not satisfy a Hardy-Weinburg equilibrium p-value > 5.7x10-7 (919 markers), a call rate >95% (97,718 markers), and a minor allele frequency >0.01 (1%) (119,246 markers – includes CNV's) were excluded. To account for population stratification, the first five principal components were calculated using a subset of 42,888 SNPS that were not in LD with each other. Principal component analysis was conducted using the EIGENSTRAT program. Raine Study was imputed against the 1000 Genomes Phase 1 Europeans (November 23, 2010 release) using MACH v 2.3.0 software. A minimum passing threshold of 0.3 on the Rsq metric and a MAF>0.01 were applied to ~30 million imputed SNP. At the 20-year follow-up participants completed a comprehensive eye assessment that included visual acuity, orthoptic assessment and cycloplegic autorefraction, as well as several ocular biometric variables and multiple ophthalmic photographs of the anterior and posterior segments. Using the 20 year follow-up examination refractive error phenotypes, 348 Caucasian participants aged 20 years or older with high quality genotypes and known spherical equivalent refraction and educational level were included in the current analysis. ProbABEL 0.4.1 was used to perform G×E interaction analysis assuming an additive model with age, sex and the first two principal components fitted as covariates. Linear regression adjusting for age, sex and the first two principal components was performed using mach2qtl to estimate association of each SNP with spherical equivalent refraction.

Rotterdam Study (RS1, RS2, RS3)

The Rotterdam Study is a prospective population-based cohort study in the elderly living in Ommoord, a suburb of Rotterdam, the Netherlands. Details of the study are described elsewhere [54]. In brief, the Rotterdam Study consists of 3 independent cohorts: RS1, RS2, and RS3. For the current analysis, 5,422residents aged 55 years and older were included from RS1, 1,973 participants aged 55 and older from RS2, and 1,971 aged 45 and older from RS 3. 99% of subjects were of Caucasian ancestry. Participants underwent multiple physical examinations with regular intervals from 1991 to present, including a non-dilated automated measurement of refractive error using a Topcon RM-A2000 autorefractor. All measurements in RS-1–3 were conducted after the Medical Ethics Committee of the Erasmus University had approved the study protocols and all participants had given a written informed consent in accordance with the Declaration of Helsinki.

DNA was extracted from blood leucocytes according to standard procedures. Genotyping of SNPs was performed using the Illumina Infinium II HumanHap550 chip v3.0 array (RS-I); the HumanHap550 Duo Arrays and the Illumina Human610-Quad Arrays (RS-II), and the Human 610 Quad Arrays Illumina (RS-III). Samples with low call rate (<97.5%), with excess autosomal heterozygosity (>0.336), or with sex-mismatch were excluded, as were outliers identified by the identity-by-state clustering analysis (outliers were defined as being >3 s.d. from population mean or having identity-by-state probabilities >97%). We used genomic control to obtain optimal and unbiased results and applied the inverse variance method of each effect size estimated for both autosomal SNPs that were genotyped and imputed in both cohorts. A set of genotyped input SNPs with call rate >98%, with

minor allele frequency >0.01, and with Hardy-Weinberg P value $>10^{-6}$ was used for imputation. We used Minimac to impute to 1000G (phase 1, March 2012). For each imputed SNP, a reliability of imputation was estimated as the ratio of the empirically observed dosage variance to the expected binomial dosage variance (O/E ratio). GWAS GxE analyses were performed using ProbABEL.

TwinsUK

The TwinsUK adult twin registry based at St. Thomas' Hospital in London is a volunteer cohort of over 10,000 twins from the general population [55]. Twins largely volunteered unaware of the eye studies, gave fully informed consent under a protocol reviewed by the St. Thomas' Hospital Local Research Ethics Committee and underwent non-cyclopleged autorefraction using an ARM-10 autorefractor (Takagi Ltd).

Genotyping of the TwinsUK dataset was done with a combination of Illumina arrays (HumanHap300, HumanHap610Q, 1M-Duo and 1.2MDuo 1M). Intensity data for each of the three arrays were pooled separately (with 1M-Duo and 1.2MDuo 1M pooled together) and genotypes were assigned using the Illuminus calling algorithm . We applied similar quality control criteria to each dataset and merged them. Pre-phasing was done with SHAPE-IT software and imputation was performed using the IMPUTE v2 using 1000 Genomes haplotypes-Phase I integrated variant set release (v3) in NCBI build 37 (hg) coordinates. GWAS GxE analyses were performed using Quicktest and only one twin for each pair was included in the analysis to overcome family structure issues.

Wisconsin Epidemiologic Study of Diabetic Retinopathy (WESDR)

WESDR is an observational cohort study of diabetes complications (1979-2007)[56]. The study protocol has been approved by the Research Ethics Board of The Hospital for Sick Children. Subjective refraction was measured following standard protocols at each follow-up visit (roughly every 5 years). In the current study the first available refractive measurement after age 25 was used.

Subjects with type 1 diabetes from WESDR were genotyped using Illumina HumanOmni1-Quad BeadChip assay. Individuals showing gender mismatch with typed X-linked markers (n=8), cryptic relatedness (n=5), high autosomal heterozygosity (n=6), call rate <0.95 (n=30), as well as ethnicities other than "white" were not included in the analysis. Population genetic approaches based on multi-dimensional scaling implemented in PLINK v1.07 were used to identify and exclude ethnically admixed individuals. Imputation was performed in IMPUTE v2.3.0 using integrated haplotypes from 1000 Genomes Phase I as reference (IMPUTE2 chooses the best custom reference set for each individual internally). The GxE regression model accounted for age, gender and the first two principal components.

Young Finns Study (YFS)

The YFS cohort is a Finnish longitudinal population study sample on the evolution of cardiovascular risk factors from childhood to adulthood[57]. The first cross-sectional study was conducted in the year 1980 in five different centers. It included 3,596 participants in the age groups of 3, 6, 9, 12, 15, and 18, who were randomly chosen from the national population register. After the baseline in 1980 these subjects have been re-examined in 1983 and 1986 as young individuals, and in 2001, 2007 and 2011 as older individuals. For the current analysis a subsample from the newest (2011) follow-up was used from four centers (N=1480) where the refractive error measurements data from both eyes were available. The 1st ethics committee of the Hospital District of Southwest Finland has approved the study protocol and all participants provided written informed consent. This study was carried out in accordance with the recommendations of the Declaration of Helsinki.

Genomic DNA was extracted from peripheral blood leukocytes using a commercially available kit and Qiagen BioRobot M48 Workstation according to the manufacturer's instructions (Qiagen, Hilden, Germany). Genotyping was done for 2,556 samples using custom build Illumina Human 670k BeadChip at Welcome Trust Sanger

Institute. Genotypes were called using Illuminus clustering algorithm. 56 samples failed Sanger genotyping pipeline QC criteria (i.e., duplicated samples, heterozygosity, low call rate, or Sequenom fingerprint discrepancy). From the remaining 2,500 samples one sample failed gender check, three was removed due to low genotyping call rate (< 0.95) and 54 samples for possible relatedness (pi-hat > 0.2) . 11,766 SNPs were excluded based on Hardy–Weinberg equilibrium (HWE) test ($p \leq 10^{-6}$), 7,746 SNPs failed missingness test (call rate < 0.95) and 34,596 SNPs failed frequency test (MAF < 0.01). After quality control there were 2,442 samples and 546,677 genotyped SNPs available for further analysis [58]. Genotype imputation was performed using IMPUTE2 and 1000 Genomes Phase I Integrated Release Version 3 (Mar 2012) samples as a reference. GWAS GxE analyses were performed using Quicktest with age, sex and the first three principal components (to adjust for population stratification) included in the model as covariates.

Beijing Eye Study (BES)

The BES is a population-based cohort of Han Chinese in the rural region and in the urban region of Beijing in North China. The Medical Ethics Committee of the Beijing Tongren Hospital approved the study protocol and all participants gave informed consent, according to the Declaration of Helsinki. At baseline (2001), 4439 individuals out of 5324 eligible individuals aged 40 years or older participated (response rate: 83.4%). In the years 2006 and 2011, the study was repeated by re-inviting all participants from the survey from 2001 to be re-examined. Out of the 4439 subjects examined in 2001, 3251 (73.2%) subjects returned for the follow-up examination in 2006, and 2695 (60.7%) subjects returned for the follow-up examination in 2011. For all subjects, visual acuity was measured. Automatic refractometry (Auto Refractometer AR-610, Nidek Co., Ltd, Tokyo, Japan) was performed if uncorrected visual acuity was lower than 1.0. The values obtained by automatic refractometry were verified and refined by subjective refractometry. Refraction data collected in 2011 was used in the analysis. In the survey of 2006, blood samples were taken from 2,929 (90.1%), and DNA was extracted from blood leucocytes according to standard procedures. We performed genotyping using Illumina Human610-Quad BeadChip in 988 subjects [59]. Of them, we excluded 151 with cryptic relatedness during sample QC procedure. Additional 259 Individuals with cataract surgery or missing refraction data were also excluded. This left a total of 585 individuals for analysis. Linear regression analyses for SE were performed at each SNP using 585 individuals with age, sex, education, SNP x education, and the first two principal components (to adjust for population stratification) included in the model.

Nagahama Prospective Genome Cohort for the Comprehensive Human Bioscience (Nagahama)

The Nagahama Prospective Genome Cohort for the Comprehensive Human Bioscience (the Nagahama Study) is a community-based prospective cohort study that aims to determine the prevalence and risk factors of various diseases in a community. The details of study design and methodology have been described elsewhere[60]. In brief, residents of Nagahama City who satisfied the following criteria were recruited as participants and were examined between November 2008 and November 2010: 1) age 30 and 74 years; 2) ability to participate on one's own; 3) no significant problems communicating in Japanese; 4) no current serious diseases/symptoms or health issues; and 5) voluntarily decided to participate in this study. A total of 9,804 Japanese individuals participated in the Nagahama Study. All the participants in the Nagahama Study had their axial length (millimeter [mm]; IOL Master, Carl Zeiss Meditec, Dublin, CA, USA), spherical equivalent (diopter [D]; ARK-530A, Nidek, Aichi, Japan), and corneal curvature (mm; ARK-530A, Nidek) measured for both eyes. Color fundus photographs were also obtained from all participants (CR-DG10, Canon, Tokyo, Japan). Of the participants, 3,712 individuals were genome-scanned using HumanHap610K Quad Arrays, HumanOmni2.5M Arrays, and/or HumanExome Arrays (Illumina Inc., San Diego, California, USA). After our standard quality control, genomic imputation was performed on 192 participants' data that had been genotyped by every platform. Finally, the data that consists of 1,756,611 SNPs of 3,248 individuals were fixed. All study procedures were approved by Ethnics committee of Kyoto University Graduate School of Medicine.

Singapore Chinese Eye Study (SCES)

Similar to SINDI, the Singapore Chinese Eye Study (SCES) is a population-based cross-sectional study of eye diseases in Chinese adults 40 years of age or older residing in the southwestern part of Singapore. The methodology of the SCES study has been described in detail previously. Between 2009 and 2011, 3353 (72.8%) of 4605 eligible individuals underwent a comprehensive ophthalmologic examination, using the same protocol as SINDI [61]. Genome-wide genotyping using was done in a subset of SCES participants using Illumina Human610-Quad BeadChip [59] (SCES-610K, n=1952) and Illumina OmniExpress (SCES-OmniE, n=615). Samples were excluded if they showed evidence of admixture, cryptic relatedness, high heterogeneity and gender discrepancies. From a starting number of 1952 individuals, three samples had per-sample call rate of <95% and were removed from analysis. A total of 21 individuals showed evidence of admixture and were consequently excluded. Biological relationship verification revealed a total of 29 sample pairs with cryptic relatedness. For these, the sample with the lower call rate was removed. In addition, further 14 samples with impossible biological sharing or heterogeneity, probably because of contamination, were removed, as well as two individuals who were removed due to gender discrepancies. PC analysis of the remaining individuals for SCES against the 1000 genomes phase 1 cosmopolitan panel haplotypes (March 2012 release) did not show the cohort to be dissimilar in ancestry, and therefore no PCs were used to correct for any underlying population substructure in the analysis performed. Individuals were excluded from the study if they had cataract surgery and missing refraction data. Linear regression analyses of SE with gene and education interaction were performed using 1710 individuals in SCES-610K and 543 in SCES-OmniE with age and sex included in the model as covariates.

***Singapore Malay Eye Study (SIMES*)**

SiMES is a population-based prevalence survey of Malay adults aged 40 to 79 years living in Singapore that was conducted between August of 2004 and June of 2006[61]. From a Ministry of Home Affairs random sample of 16,069 Malay adults in the Southwestern area, an age-stratified random sampling strategy was used in selecting 1400 from each decade from age 40 years onward (40–49, 50–59, 60–69, and 70–79 years). The 4168 eligible participants from the sampling frame, while 3280 (78.7%) participated. Genome-wide genotyping was performed in 3072 individuals[59; 62].

Total of 3072 DNA samples were genotyped using the Illumina Human 610 Quad Beadchips [62; 63]. Using the same quality control criteria, we omitted a total of 530 individuals including those of subpopulation structure (n=170), cryptic relatedness (n=279), excessive heterozygosity or high missingness rate > 5% (n=37), and gender discrepancy (n=44). A total of 2165 individuals were over age 25 and had high quality genotypes and phenotypes for astigmatism. After the removal of the samples, SNP QC was then applied on a total of 579,999 autosomal SNPs for the 2542 post-QC samples. The same QC methods used for SCES were applied to the SiMES genotyping samples. Linear regression analyses of SE with gene and education interaction were performed using 2256 individuals with age, sex and the first two principal components (to adjust for population stratification) included in the model as covariates.

Singapore Indian Eye Study (SINDI)

SINDI is a population-based survey of major eye diseases [64] in ethnic Indians aged 40 to 80 years living in the South-Western part of Singapore and was conducted from August 2007 to December 2009. In brief, 4,497 Indian adults were eligible and 3400 participated. Genome-wide genotyping was performed in 2,953 individuals [63]. Participants were excluded from the study if they had cataract surgery and missing refraction data.
The Illumina Human610 Quad Beadchips was used for genotyping all DNA samples from SINDI (n=2593). We excluded 415 subjects from the total of 2953 genotyped samples based on: excessive heterozygosity or high missingness rate > 5% (n=34) , cryptic relatedness (n=326), issues with population structure ascertainment (n=39) and gender discrepancies (n=16). This left a total of 2,538 individuals with 579,999 autosomal SNPs and 2,088 of

these individuals were also over age 20 and had phenotype data. During SNP QC procedure, SNPs were excluded based on (i) high rates of missingness (> 5%) ; (ii) monomorphism or MAF < 1% ; or (iii) genotype frequencies deviated from HWE ($P < 1 \times 10^{-6}$). Linear regression analyses of SE with gene and education interaction were performed using 2088 individuals with age, sex and the first two principal components (to adjust for population stratification) included in the model as covariates.

Singapore Prospective Study Program (SP2-1M; SP2-610)

Samples of SP2 were from a revisit of two previously conducted population-based surveys carried out in Singapore between 1992 and 1998, including the National Health Survey 1992 and the National Health Survey 1998[65]. These studies comprise random samplings of individuals stratified by ethnicity from the entire Singapore population. A total of 8266 subjects were invited in this follow-up survey and 6301 (76.1% response rate) subjects completed the questionnaire, of which 4056 (64.4% of those who completed the questionnaire) also attended the health examination and donated blood specimens. The present GWA genotyping for SP2 involved individuals of Chinese descent only (n=2867)[66].

Of the 2,867 blood-derived DNA samples, 1,459 samples were genotyped on the 610-Quad (SP2-610) and 1,016 samples on the 1M-Duov3 (SP2-1M). We excluded 443 individuals on the following conditions, sample call rates of less than 95%, excessive heterozygosity, cryptic relatedness by IBS, population structure ascertainment, and gender discrepancies as listed in the main text. During the SNPs QC procedure, we excluded SNPs with low genotyping call rates (> 5% missingness) or monomorphic, with MAF < 1%, or with significant deviation from HWE ($P< 10^{-6}$). This yielded a post-QC set of 462,580 SNPs. We additionally assessed the SNPs that are present on different platforms for extreme variations in allele frequencies with a 2-degree of freedom chi-square test of proportions, removing 62 SNPs with *P*-values < 0.0001. A total of 811 individuals in SP2-1M and 854 individuals in SP2-610 had both high quality genotype data and SE data and were used in the Linear regression analyses of SE with gene and education interaction, adjusting for age and sex.

Strabismus, Amblyopia and Refractive Error Study (STARS)

The Strabismus, Amblyopia and Refractive Error Study in Singaporean Chinese Preschoolers (STARS) Family study is a family-based study nested in a prevalence survey of Singaporean preschool children (n=3,009) conducted from March 2008 to March 2010[67]. The biological parents of STARS probands were invited to enroll in the STARS Family study. A total of 1,451 samples from 440 nuclear families were genotyped using Illumina Human610 Quad Beadchips. The 741 parents who had phenotype data and who also had available, high quality GWAS genotypes were used in the current study. Linear regression analyses of SE including gene and education interaction were performed with age, sex included in the model as covariates.

All Singapore studies adhere to the Declaration of Helsinki. Ethics approvals have been obtained from the Institutional Review Boards of the Singapore Eye Research Institute, Singapore General hospital, National University of Singapore and National Healthcare Group, Singapore. In all cohorts, participants provided written, informed consent at the recruitment into the studies.

Supplementary Note 2 Acknowledgements

We would like to acknowledge the following agencies and persons:

The **Alienor** study is supported by laboratoires Théa (Clermont-Ferrand, France). The Three-City study is conducted under a partnership agreement between the *Institut National de la Santé et de la Recherche Médicale* (INSERM), the University of Bordeaux and Sanofi-Aventis. The *Fondation pour la Recherche Médicale* funded the preparation and initiation of the study. The Three-City study is also supported by the *Caisse Nationale Maladie des Travailleurs Salariés*, *Caisse Nationale de Solidarité pour l'Autonomie, Direction Générale de la Santé*, MGEN, *Institut de la Longévité*, *Conseils Régionaux d'Aquitaine et Bourgogne*, *Fondation de France*, Ministry of Research-INSERM Programme "*Cohortes et collections de données biologiques*", *Agence Nationale de la Recherche* ANR PNRA 2006 and LongVie 2007, and the "*Fondation Plan Alzheimer*" (FCS 2009-2012). Laboratoires Théa participated in the design of the Alienor study, but none of the sponsors participated in the collection, management, statistical analysis and interpretation of the data, not in the preparation, review or approval of the present manuscript.

The UK Medical Research Council and the Wellcome Trust (Grant ref: 102215/2/13/2) and the University of Bristol provide core support for **ALSPAC**. This research was specifically funded by grant MC_UU_12013/3&4 from the UK Medical Research Council, a NIHR Career Development Fellowship (CDF-2009-02-35) and a Wellcome Trust ISSF Populations Pilot Award (grant 508353/509506). GWAS data were generated by Sample Logistics and Genotyping Facilities at the Wellcome Trust Sanger Institute and LabCorp (Laboratory Corportation of America) using support from 23andMe. ALSPAC thanks all the families who took part in this study, the midwives for their help in recruiting them, and the whole ALSPAC team, which includes interviewers, computer and laboratory technicians, clerical workers, research scientists, volunteers, managers, receptionists and nurses.

AREDS1c was supported by contracts from National Eye Institute/National Institutes of Health, Bethesda, MD, with additional support from Bausch & Lomb Inc, Rochester, NY. The genotyping costs were supported by the National Eye Institute (R01EY020483 and RO1EY024233 to D.S.) and some of the analyses were supported by the Intramural Research Program of the National Human Genome Research Institute, National Institutes of Health, USA (JEBW, CLS, TSA). AREDS acknowledges Frederick Ferris and Emily Chew, National Eye Institute, National Institutes of Health, Bethesda, MD; and the Center for Inherited Disease Research, Baltimore, MD where SNP genotyping was carried out. The investigators gratefully acknowledge the advice and guidance of Hemin Chin of the National Eye Institute.

BATS (Australian Twins) were supported by an Australian National Health and Medical Research Council (NHMRC) Enabling Grant (2004-2009, 350415, 2005-2007); Clifford Craig Medical Research Trust; Ophthalmic Research Institute of Australia; American Health Assistance Foundation; Peggy and Leslie Cranbourne Foundation; Foundation for Children; Jack Brockhoff Foundation; National Institutes of Health/National Eye Institute (RO1EY01824601 (2007-2010)); Pfizer Australia Senior Research Fellowship (to D.A.M.); and Australian NHMRC Career Development Award (to S.M.). Genotyping was funded by an NHMRC Medical Genomics Grant; US National Institutes of Health/National Eye Institute (1RO1EY018246 (to T.L.Y)), Australian sample imputation analyses were carried out on the Genetic Cluster Computer which is financially supported by the Netherlands Scientific Organization (NWO48005003). Australian Twins thank Nicholas Martin, Scott Gordon, Dale Nyholt, Sarah Medland, Brian McEvoy, Margaret Wright, Anjali Henders, Megan Campbell for ascertaining and processing genotyping data, and research nurses and managers at QIMR Berghofer for data collections on education level in the 19UP study; Jane MacKinnon, Shayne Brown, Lisa Kearns, Jonathan Ruddle, Paul Sanfilippo,

Sandra Staffieri, Olivia Bigault, Colleen Wilkinson, Jamie Craig, Yaling Ma, Julie Barbour for assisting with clinical examinations; and Dr Camilla Day and staff at the Center for Inherited Disease Research.

BMES was supported by the Australian National Health & Medical Research Council (NH&MRC), Canberra Australia (974159, 211069, 457349, 512423, 475604, 529912); the Centre for Clinical Research Excellence in Translational Clinical Research in Eye Diseases; NH&MRC research fellowships (358702, 632909 to J.J.W, 1028444 to P.N.B.); and the Wellcome Trust, UK as part of Wellcome Trust Case Control Consortium 2 (A. Viswanathan, P. McGuffin, P. Mitchell, F. Topouzis, P. Foster) for genotyping costs of the entire BMES population (085475B08Z, 08547508Z, 076113). The Centre for Eye Research Australia receives Operational Infrastructure Support from the Victorian government. BMES acknowledges Elena Rochtchina from the Centre for Vision Research, Department of Ophthalmology and Westmead Institute for Medical Research, University of Sydney (NSW Australia); John Attia, Rodney Scott, Elizabeth G. Holliday from the University of Newcastle (Newcastle, NSW Australia); Maria Schache and Andrea J. Richardson from the Centre for Eye Research Australia, University of Melbourne; Michael T. Inouye, Medical Systems Biology, Department of Pathology & Department of Microbiology & Immunology, University of Melbourne (Victoria, Australia); Ananth Viswanathan, Moorfields Eye Hospital (London, UK); Paul J. Foster, NIHR Biomedical Research Centre for Ophthalmology, UCL Institute of Ophthalmology & Moorfields Eye Hospital (London); Peter McGuffin, MRC Social Genetic and Developmental Psychiatry Research Centre, Institute of Psychiatry, King's College (London, United Kingdom); Fotis Topouzis, Department of Ophthalmology, School of Medicine, Aristotle University of Thessaloniki, AHEPA Hospital (Thessaloniki, Greece); Xueling Sim, National University of Singapore.

The **CROATIA** studies were funded by grants from the Medical Research Council (UK) and from the Republic of Croatia Ministry of Science, Education and Sports (10810803150302); and the CROATIA-Korcula genotyping was funded by the European Union framework program 6 project EUROSPAN (LSHGCT2006018947). The CROATIA studies acknowledges Dr. Goran Bencic, Biljana Andrijević Derk, Valentina Lacmanović Lončar, Krešimir Mandić, Antonija Mandić, Ivan Škegro, Jasna Pavičić Astaloš, Ivana Merc, Miljenka Martinović, Petra Kralj, Tamara Knežević and Katja Barać-Juretić as well as the recruitment team from the Croatian Centre for Global Health, University of Split and the Institute of Anthropological Research in Zagreb for the ophthalmological data collection; Peter Lichner and the Helmholtz Zentrum Munchen (Munich, Germany), AROS Applied Biotechnology, Aarhus, Denmark and the Wellcome Trust Clinical facility (Edinburgh, Scotland) for the SNP genotyping all studies; Susan Campbell and Pau Navarro for genetic data preparation.

The **DCCT/**EDIC Research Group is sponsored through research contracts from the National Institute of Diabetes, Endocrinology and Metabolic Diseases of the National Institute of Diabetes and Digestive and Kidney Diseases (NIDDK) and the National Institutes of Health. Clinical data and DNA from the DCCT study is available through the National Institute of Diabetes and Digestive and Kidney Diseases repository at https://www.niddkrepository.org/niddk/home.do. The authors are grateful to the patients and researchers of DCCT/EDIC. ADP is the guarantor for DCCT.

EGCUT studies were financed by University of Tartu (grant "Center of Translational Genomics") by Estonian Government (grant # TP1GV140601, grant #ETF9353) and by European Commission through the European Regional Development Fund in the frame of grant "center of Excellence in genomic" and by an Estonian Reseach Infrastructure's Roadmap and through FP7 grant #313010.

EPIC-Norfolk infrastructure and core functions are supported by grants from the Medical Research Council (G1000143) and Cancer Research UK (C864/A14136). The clinic for the third health examination was funded by Research into Ageing (262). Mr Khawaja is a Wellcome Trust Clinical Research Fellow. Miss Chan is a joint Medical Research Council/Royal College of Ophthalmologists Research Fellow, and received additional support

from the International Glaucoma Association. Professor Foster has received additional support from the Richard Desmond Charitable Trust (via Fight for Sight) and the Department for Health through the award made by the National Institute for Health Research to Moorfields Eye Hospital and the UCL Institute of Ophthalmology for a specialist Biomedical Research Centre for Ophthalmology.

Framingham Eye Study (FES) is a sub-study of the Framingham Heart Study, which is conducted and supported by the National Heart, Lung, and Blood Institute (NHLBI) in collaboration with Boston University (Contract No. N01-HC-25195). These data were obtained from the NIH repository dbGaP (accession numbers phs000007/HMB-IRB-MDS and phs000007/HMB-IRB-NPU-MDS). This manuscript was not prepared in collaboration with investigators of the Framingham Heart Study and does not necessarily reflect the opinions or views of the Framingham Heart Study, Boston University, or NHLBI. Funding for SHARe Affymetrix genotyping was provided by NHLBI Contract N02-HL-64278. SHARe Illumina genotyping was provided under an agreement between Illumina and Boston University. Funding for the collection of FES phenotype data was supported by the National Eye Institute (ZIAEY000403; N01EY22112, N01EY92109). The current analyses were supported by intramural funds of the National Human Genome Research Institute, NIH, USA (to JEBW, QL, CLS, and RW).

FITSA was supported by ENGAGE (FP7-HEALTH-F4-2007, 201413); European Union through the GENOMEUTWIN project (QLG2-CT-2002-01254); the Academy of Finland Center of Excellence in Complex Disease Genetics (213506, 129680); the Academy of Finland Ageing Programme; and the Finnish Ministry of Culture and Education and University of Jyväskylä. For FITSA the contributions of Antti-Pekka Sarin, Emmi Tikkanen, Samuli Ripatti, Markku Kauppinen, Taina Rantanen and Jaakko Kaprio are acknowledged.

The **Gutenberg Health Study (GHS)** was funded through the government of Rheinland-Pfalz ("Stiftung Rheinland Pfalz für Innovation", contract AZ 961-386261/733); the research programs "Wissen schafft Zukunft" and the "Center for Translational Vascular Biology (CTVB)" of the Johannes Gutenberg-University of Mainz, the German National Genome Research Network ''NGFNplus'' by the Federal Ministry of Education and Research, Germany (A301GS0833) and its contracts with Boehringer Ingelheim, PHILIPS Medical Systems and Novartis Pharma, including an unrestricted grant for the Gutenberg Health Study. We thank all study participants for their willingness to provide data for this research project and we are indebted to all coworkers for their enthusiastic commitment.

KORA was financed by the Helmholtz Center Munich, German Research Center for Environmental Health; the German Federal Ministry of Education and Research; the State of Bavaria; the German National Genome Research Network (NGFN-2 and NGFNPlus) (01GS0823); Munich Center of Health Sciences as part of LMUinnovativ; the genotyping was carried out by the Center for Inherited Disease Research, Baltimore, MD, and was supported by the National Eye Institute (R01 EY020483 and RO1EY024233 to D.S.). Some of the analyses were supported by the Intramural Research Program of the National Human Genome Research Institute, NIH, USA.

OGP Talana was supported by grants from the Italian Ministry of Education, University and Research (5571DSPAR2002, 718Ric2005). OGP Talana thanks the Ogliastra population and the municipal administrators for their collaboration to the project and for economic and logistic support.

ORCADES was supported by the Chief Scientist Office of the Scottish Government, the Royal Society, the Medical Research Council Human Genetics Unit and the European Union framework program 6 EUROSPAN project (LSHGCT2006018947). ORCADES acknowledges the invaluable contributions of Lorraine Anderson and the research nurses in Orkney, in particular Margaret Pratt who performed the eye measurements,as well as the administrative team in Edinburgh University; and the Wellcome Trust Clinical facility (Edinburgh, Scotland) for

DNA extraction; and Peter Lichner and the Helmholtz Zentrum Munchen (Munich, Germany) for genotyping; and Mirna Kirin for the genetic data imputation.
The core management of the **RAINE** Study is funded by The University of Western Australia (UWA), The Telethon Institute for Child Health Research, Raine Medical Research Foundation, UWA Faculty of Medicine, Dentistry and Health Sciences, Women's and Infant's Research Foundation and Curtin University. Genotyping was funded by NHMRC project grant 572613. Support for the Raine Eye Health Study was provided by NHMRC Grant 1021105, Lions Eye Institute, the Australian Foundation for the Prevention of Blindness and Alcon Research Institute. The Raine Eye Health Study authors thank the Raine eye health study participants and their families. They also thank the Raine Study management, Craig Pennell, Jenny Mountain and the team at TICHR and LEI for cohort co-ordination and data collection, particularly: Seyhan Yazar, Hannah Forward,Charlotte McKnight, Alex Tan, Alla Soloshenko, Sandra Oates, and Diane Wood.

The **Rotterdam Study (RSI, RS2, RS3)** and **ERF** are supported by the Netherlands Organisation of Scientific Research NWO Investments (nr. 175.010.2005.011, 911-03-012, Vidi nr. 91796357 to C.C.W.K.); the Research Institute for Diseases in the Elderly (014-93-015; RIDE2), the Netherlands Genomics Initiative (NGI)/Netherlands Organisation for Scientific Research (NWO) project nr. 050-060-810; Erasmus Medical Center and Erasmus University, Rotterdam, The Netherlands; Netherlands Organization for Health Research and Development (ZonMw); UitZicht; the Ministry of Education, Culture and Science; the Ministry for Health, Welfare and Sports; the European Commission (DG XII); the Municipality of Rotterdam; Center for Medical Systems Biology of NGI; Lijf en Leven; M.D. Fonds; Henkes Stichting; Stichting Nederlands Oogheelkundig Onderzoek; Swart van Essen; Bevordering van Volkskracht; Blindenhulp; Landelijke Stichting voor Blinden en Slechtzienden; Rotterdamse Vereniging voor Blindenbelangen; OOG; Algemene Nederlandse Vereniging ter Voorkoming van Blindheid; the Rotterdam Eye Hospital Research Foundation; ErasmusMC Trustfonds; Stichting Glaucoomfonds; Stichting Nederlands Oogheelkundig Onderzoek (SNOO); Ter Meulen Fonds; Stichting tot Verbetering Lot van de Blinden; Stichting Dondersfonds; MolMed; LSBS; Stichting Nelly Reef Fund; Stichting Simonsfonds; Novartis; Alcon; Topcon Europe. We thank Pascal Arp, Mila Jhamai, Marijn Verkerk, Lizbeth Herrera and Marjolein Peters, Patricia van Hilten, Jeanette Vergeer, Sander Bervoets for their help in creating the GWAS database, and Karol Estrada and Maksim V. Struchalin for their support in creation and analysis of imputed data. We thank Ada Hooghart, Corina Brussee, Riet Bernaerts-Biskop for the ophthalmological examinations. The authors are grateful to the study participants, the staff from the Rotterdam Study and the participating general practitioners and pharmacists.

TwinsUK was funded by the Wellcome Trust; European Community's Seventh Framework Programme (FP7/2007-2013)/grant agreement HEALTH-F2-2008-201865-GEFOS and (FP7/2007-2013), ENGAGE project grant agreement HEALTH-F4-2007-201413 and the FP-5 GenomEUtwin Project (QLG2-CT-2002-01254). The study also receives support from the Dept of Health via the National Institute for Health Research (NIHR) comprehensive Biomedical Research Centre award to Guy's & St Thomas' NHS Foundation Trust in partnership with King's College London. TDS is an NIHR senior Investigator. The project also received support from a Biotechnology and Biological Sciences Research Council (BBSRC) project grant. (G20234) .The authors acknowledge the funding and support of the National Eye Institute via an NIH/CIDR genotyping project (PI: Terri Young; 1RO1EY018246). Genotyping of TwinsUK samples: We thank the staff from the Genotyping Facilities at the Wellcome Trust Sanger Institute for sample preparation, Quality Control and Genotyping led by Leena Peltonen and Panos Deloukas; Le Centre National de Génotypage, France, led by Mark Lathrop, for genotyping; Duke University, North Carolina, USA, led by David Goldstein, for genotyping; and the Finnish Institute of Molecular Medicine, Finnish Genome Center, University of Helsinki, led by Aarno Palotie. Genotyping was also performed by CIDR as part of an NEI/NIH project grant. NS is supported by the Wellcome Trust (Core Grant Number 091746/Z/10/Z).

WESDR was supported by NEI (grants R01EY03083 and EY016379, Ronald and Barbara Klein) and a Research to Prevent Blindness Senior Scientific Investigator Award (Ronald and Barbara).
Young Finns Study was financially supported by the Academy of Finland (134309 (Eye), 126925, 121584, 124282, 129378 (Salve), 117787 (Gendi), and 41071 (Skidi)); the Social Insurance Institution of Finland, Kuopio, Tampere; Turku University Hospital Medical Funds (grant 9M048 and 9N035 to T.L.); Juho Vainio Foundation; Paavo Nurmi Foundation; Finnish Foundation of Cardiovascular Research and Finnish Cultural Foundation; Tampere Tuberculosis Foundation; and Emil Aaltonen Foundation (to T.L.). The expert technical assistance in the statistical analyses by Irina Lisinen and Ville Aalto are gratefully acknowledged.

Nagahama Study was supported by Grants-in-aid for scientific research (No. 2429082, No24592624) from the Japan Society for the Promotion of Science, Tokyo, Japan, Health Labour Sciences Research Grant H23-003, and Supporting Program for Interaction-based Initiative Team Studies.

Beijing Eye Study was supported by National Natural Science Foundation of China (grant 81170890).

The **Singapore studies** (**SCES, SIMES, SINDI, SP2)** were supported by the National Medical Research Council, Singapore (NMRC 0796/2003, NMRC 1176/2008, STaR/0003/2008; CG/SERI/2010), Biomedical Research Council, Singapore (06/1/21/19/466, 09/1/35/19/616 and 08/1/35/19/550). The Strabismus, Amblyopia, and Refractive Error Study of Preschool Children (**STARS**) was supported by a NMRC grant (1176/2008). The Singapore Tissue Network and the Genome Institute of Singapore, Agency for Science, Technology and Research, Singapore provided services. The authors acknowledge the medical editing assistance from the Clinical Sciences, Duke-NUS Graduate Medical School, Singapore.

Supplementary References

1. Warde-Farley, D., Donaldson, S.L., Comes, O., Zuberi, K., Badrawi, R., Chao, P., Franz, M., Grouios, C., Kazi, F., Lopes, C.T., et al. (2010). The GeneMANIA prediction server: biological network integration for gene prioritization and predicting gene function. Nucleic acids research 38, W214-220.
2. Verhoeven, V.J., Hysi, P.G., Wojciechowski, R., Fan, Q., Guggenheim, J.A., Hohn, R., Macgregor, S., Hewitt, A.W., Nag, A., Cheng, C.Y., et al. (2013). Genome-wide meta-analyses of multiancestry cohorts identify multiple new susceptibility loci for refractive error and myopia. Nature genetics 45, 314-318.
3. Kiefer, A.K., Tung, J.Y., Do, C.B., Hinds, D.A., Mountain, J.L., Francke, U., and Eriksson, N. (2013). Genome-wide analysis points to roles for extracellular matrix remodeling, the visual cycle, and neuronal development in myopia. PLoS genetics 9, e1003299.
4. Merico, D., Isserlin, R., Stueker, O., Emili, A., and Bader, G.D. (2010). Enrichment map: a network-based method for gene-set enrichment visualization and interpretation. PloS one 5, e13984.
5. Hunter, S., Apweiler, R., Attwood, T.K., Bairoch, A., Bateman, A., Binns, D., Bork, P., Das, U., Daugherty, L., Duquenne, L., et al. (2009). InterPro: the integrative protein signature database. Nucleic acids research 37, D211-215.
6. Saw, S.M., Hong, C.Y., Chia, K.S., Stone, R.A., and Tan, D. (2001). Nearwork and myopia in young children. Lancet 357, 390.
7. Zheng, Y., Ding, X., Chen, Y., and He, M. (2013). The Guangzhou Twin Project: an update. Twin research and human genetics : the official journal of the International Society for Twin Studies 16, 73-78.
8. Boyd, A., Golding, J., Macleod, J., Lawlor, D.A., Fraser, A., Henderson, J., Molloy, L., Ness, A., Ring, S., and Davey Smith, G. (2013). Cohort Profile: The 'Children of the 90s'--the index offspring of the Avon Longitudinal Study of Parents and Children. Int J Epidemiol 42, 111-127.
9. Fraser, A., Macdonald-Wallis, C., Tilling, K., Boyd, A., Golding, J., Davey Smith, G., Henderson, J., Macleod, J., Molloy, L., Ness, A., et al. (2013). Cohort Profile: the Avon Longitudinal Study of Parents and Children: ALSPAC mothers cohort. Int J Epidemiol 42, 97-110.
10. Wagner, A.H., Anand, V.N., Wang, W.H., Chatterton, J.E., Sun, D., Shepard, A.R., Jacobson, N., Pang, I.H., Deluca, A.P., Casavant, T.L., et al. (2013). Exon-level expression profiling of ocular tissues. Experimental eye research 111, 105-111.
11. Zhang, H., Pao, L.I., Zhou, A., Brace, A.D., Halenbeck, R., Hsu, A.W., Bray, T.L., Hestir, K., Bosch, E., Lee, E., et al. (2014). Deorphanization of the human leukocyte tyrosine kinase (LTK) receptor by a signaling screen of the extracellular proteome. Proceedings of the National Academy of Sciences of the United States of America 111, 15741-15745.
12. Gentile, V., Nicotra, M., Scaravelli, G., Antonini, G., Ambrosi, S., Saccucci, P., Adanti, S., Bottini, E., and Gloria-Bottini, F. (2014). ACP1 genetic polymorphism and spermatic parameters in men with varicocele. Andrologia 46, 147-150.
13. Fan, Q., Zhou, X., Khor, C.C., Cheng, C.Y., Goh, L.K., Sim, X., Tay, W.T., Li, Y.J., Ong, R.T., Suo, C., et al. (2011). Genome-wide meta-analysis of five Asian cohorts identifies PDGFRA as a susceptibility locus for corneal astigmatism. PLoS genetics 7, e1002402.
14. Diskin, S.J., Capasso, M., Schnepp, R.W., Cole, K.A., Attiyeh, E.F., Hou, C., Diamond, M., Carpenter, E.L., Winter, C., Lee, H., et al. (2012). Common variation at 6q16 within HACE1 and LIN28B influences susceptibility to neuroblastoma. Nature genetics 44, 1126-1130.
15. Dietz, H.C., and Pyeritz, R.E. (1995). Mutations in the human gene for fibrillin-1 (FBN1) in the Marfan syndrome and related disorders. Human molecular genetics 4 Spec No, 1799-1809.
16. Rivas, M.A., Pirinen, M., Conrad, D.F., Lek, M., Tsang, E.K., Karczewski, K.J., Maller, J.B., Kukurba, K.R., DeLuca, D.S., Fromer, M., et al. (2015). Human genomics. Effect of predicted protein-truncating genetic variants on the human transcriptome. Science 348, 666-669.

17. Huo, L., Cui, D., Yang, X., Gao, Z., Trier, K., and Zeng, J. (2013). All-trans retinoic acid modulates mitogen-activated protein kinase pathway activation in human scleral fibroblasts through retinoic acid receptor beta. Molecular vision 19, 1795-1803.
18. Grundberg, E., Small, K.S., Hedman, A.K., Nica, A.C., Buil, A., Keildson, S., Bell, J.T., Yang, T.P., Meduri, E., Barrett, A., et al. (2012). Mapping cis- and trans-regulatory effects across multiple tissues in twins. Nature genetics 44, 1084-1089.
19. Solbu, T.T., Bjorkmo, M., Berghuis, P., Harkany, T., and Chaudhry, F.A. (2010). SAT1, A Glutamine Transporter, is Preferentially Expressed in GABAergic Neurons. Front Neuroanat 4, 1.
20. Pirinen, M., Lappalainen, T., Zaitlen, N.A., Consortium, G.T., Dermitzakis, E.T., Donnelly, P., McCarthy, M.I., and Rivas, M.A. (2015). Assessing allele-specific expression across multiple tissues from RNA-seq read data. Bioinformatics.
21. Tsai, W., Morielli, A.D., and Peralta, E.G. (1997). The m1 muscarinic acetylcholine receptor transactivates the EGF receptor to modulate ion channel activity. EMBO J 16, 4597-4605.
22. Duncan, G., and Collison, D.J. (2003). Role of the non-neuronal cholinergic system in the eye: a review. Life Sci 72, 2013-2019.
23. Fujishige, K., Kotera, J., Michibata, H., Yuasa, K., Takebayashi, S., Okumura, K., and Omori, K. (1999). Cloning and characterization of a novel human phosphodiesterase that hydrolyzes both cAMP and cGMP (PDE10A). J Biol Chem 274, 18438-18445.
24. Wolloscheck, T., Spiwoks-Becker, I., Rickes, O., Holthues, H., and Spessert, R. (2011). Phosphodiesterase10A: abundance and circadian regulation in the retina and photoreceptor of the rat. Brain research 1376, 42-50.
25. Delcourt, C., Korobelnik, J.F., Barberger-Gateau, P., Delyfer, M.N., Rougier, M.B., Le Goff, M., Malet, F., Colin, J., and Dartigues, J.F. (2010). Nutrition and age-related eye diseases: the Alienor (Antioxydants, Lipides Essentiels, Nutrition et maladies OculaiRes) Study. The journal of nutrition, health & aging 14, 854-861.
26. Group, C.S. (2003). Vascular factors and risk of dementia: design of the Three-City Study and baseline characteristics of the study population. Neuroepidemiology 22, 316-325.
27. Age-Related Eye Disease Study Research, G. (2001). A randomized, placebo-controlled, clinical trial of high-dose supplementation with vitamins C and E and beta carotene for age-related cataract and vision loss: AREDS report no. 9. Arch Ophthalmol 119, 1439-1452.
28. Age-Related Eye Disease Study Research, G. (2001). A randomized, placebo-controlled, clinical trial of high-dose supplementation with vitamins C and E, beta carotene, and zinc for age-related macular degeneration and vision loss: AREDS report no. 8. Archives of ophthalmology 119, 1417-1436.
29. Clemons, T.E., Chew, E.Y., Bressler, S.B., McBee, W., and Age-Related Eye Disease Study Research, G. (2003). National Eye Institute Visual Function Questionnaire in the Age-Related Eye Disease Study (AREDS): AREDS Report No. 10. Arch Ophthalmol 121, 211-217.
30. Age-Related Eye Disease Study Research, G. (1999). The Age-Related Eye Disease Study (AREDS): design implications. AREDS report no. 1. Control Clin Trials 20, 573-600.
31. Mackey, D.A., Mackinnon, J.R., Brown, S.A., Kearns, L.S., Ruddle, J.B., Sanfilippo, P.G., Sun, C., Hammond, C.J., Young, T.L., Martin, N.G., et al. (2009). Twins eye study in Tasmania (TEST): rationale and methodology to recruit and examine twins. Twin Res Hum Genet 12, 441-454.
32. Foran, S., Wang, J.J., and Mitchell, P. (2003). Causes of visual impairment in two older population cross-sections: the Blue Mountains Eye Study. Ophthalmic epidemiology 10, 215-225.
33. Vitart, V., Bencic, G., Hayward, C., Herman, J.S., Huffman, J., Campbell, S., Bucan, K., Zgaga, L., Kolcic, I., Polasek, O., et al. (2010). Heritabilities of ocular biometrical traits in two croatian isolates with extended pedigrees. Invest Ophthalmol Vis Sci 51, 737-743.
34. Delaneau, O., Zagury, J.F., and Marchini, J. (2013). Improved whole-chromosome phasing for disease and population genetic studies. Nature methods 10, 5-6.

35. Howie, B.N., Donnelly, P., and Marchini, J. (2009). A flexible and accurate genotype imputation method for the next generation of genome-wide association studies. PLoS genetics 5, e1000529.
36. The DCCT Research Group. (1993). The effect of intensive treatment of diabetes on the development and progression of long-term complications in insulin-dependent diabetes mellitus. The New England journal of medicine 329, 977-986.
37. Paterson, A.D., Waggott, D., Boright, A.P., Hosseini, S.M., Shen, E., Sylvestre, M.P., Wong, I., Bharaj, B., Cleary, P.A., Lachin, J.M., et al. (2010). A genome-wide association study identifies a novel major locus for glycemic control in type 1 diabetes, as measured by both A1C and glucose. Diabetes 59, 539-549.
38. Riboli, E., and Kaaks, R. (1997). The EPIC Project: rationale and study design. European Prospective Investigation into Cancer and Nutrition. International journal of epidemiology 26 Suppl 1, S6-14.
39. Day, N., Oakes, S., Luben, R., Khaw, K.T., Bingham, S., Welch, A., and Wareham, N. (1999). EPIC-Norfolk: study design and characteristics of the cohort. European Prospective Investigation of Cancer. British journal of cancer 80 Suppl 1, 95-103.
40. Hayat, S.A., Luben, R., Keevil, V.L., Moore, S., Dalzell, N., Bhaniani, A., Khawaja, A.P., Foster, P., Brayne, C., Wareham, N.J., et al. (2014). Cohort profile: A prospective cohort study of objective physical and cognitive capability and visual health in an ageing population of men and women in Norfolk (EPIC-Norfolk 3). International journal of epidemiology 43, 1063-1072.
41. Khawaja, A.P., Chan, M.P., Hayat, S., Broadway, D.C., Luben, R., Garway-Heath, D.F., Sherwin, J.C., Yip, J.L., Dalzell, N., Wareham, N.J., et al. (2013). The EPIC-Norfolk Eye Study: rationale, methods and a cross-sectional analysis of visual impairment in a population-based cohort. BMJ open 3.
42. Aulchenko, Y.S., Heutink, P., Mackay, I., Bertoli-Avella, A.M., Pullen, J., Vaessen, N., Rademaker, T.A., Sandkuijl, L.A., Cardon, L., Oostra, B., et al. (2004). Linkage disequilibrium in young genetically isolated Dutch population. Eur J Hum Genet 12, 527-534.
43. Pardo, L.M., MacKay, I., Oostra, B., van Duijn, C.M., and Aulchenko, Y.S. (2005). The effect of genetic drift in a young genetically isolated population. Ann Hum Genet 69, 288-295.
44. Leibowitz, H.M., Krueger, D.E., Maunder, L.R., Milton, R.C., Kini, M.M., Kahn, H.A., Nickerson, R.J., Pool, J., Colton, T.L., Ganley, J.P., et al. (1980). The Framingham Eye Study monograph: An ophthalmological and epidemiological study of cataract, glaucoma, diabetic retinopathy, macular degeneration, and visual acuity in a general population of 2631 adults, 1973-1975. Surv Ophthalmol 24, 335-610.
45. Group, F.E.S. (1996). Familial aggregation and prevalence of myopia in the Framingham Offspring Eye Study. Archives of ophthalmology 114, 326-332.
46. Zeller, T., Wild, P., Szymczak, S., Rotival, M., Schillert, A., Castagne, R., Maouche, S., Germain, M., Lackner, K., Rossmann, H., et al. (2010). Genetics and beyond--the transcriptome of human monocytes and disease susceptibility. PLoS One 5, e10693.
47. Wichmann, H.E., Gieger, C., Illig, T., and Group, M.K.S. (2005). KORA-gen--resource for population genetics, controls and a broad spectrum of disease phenotypes. Gesundheitswesen 67 Suppl 1, S26-30.
48. Holle, R., Happich, M., Lowel, H., Wichmann, H.E., and Group, M.K.S. (2005). KORA--a research platform for population based health research. Gesundheitswesen 67 Suppl 1, S19-25.
49. Oexle, K., Ried, J.S., Hicks, A.A., Tanaka, T., Hayward, C., Bruegel, M., Gogele, M., Lichtner, P., Muller-Myhsok, B., Doring, A., et al. (2011). Novel association to the proprotein convertase PCSK7 gene locus revealed by analysing soluble transferrin receptor (sTfR) levels. Hum Mol Genet 20, 1042-1047.
50. Steffens, M., Lamina, C., Illig, T., Bettecken, T., Vogler, R., Entz, P., Suk, E.K., Toliat, M.R., Klopp, N., Caliebe, A., et al. (2006). SNP-based analysis of genetic substructure in the German population. Hum Hered 62, 20-29.
51. Biino, G., Palmas, M.A., Corona, C., Prodi, D., Fanciulli, M., Sulis, R., Serra, A., Fossarello, M., and Pirastu, M. (2005). Ocular refraction: heritability and genome-wide search for eye morphometry traits in an isolated Sardinian population. Human genetics 116, 152-159.

52. Rietveld, C.A., Medland, S.E., Derringer, J., Yang, J., Esko, T., Martin, N.W., Westra, H.J., Shakhbazov, K., Abdellaoui, A., Agrawal, A., et al. (2013). GWAS of 126,559 individuals identifies genetic variants associated with educational attainment. Science 340, 1467-1471.
53. Yazar, S., Forward, H., McKnight, C.M., Tan, A., Soloshenko, A., Oates, S.K., Ang, W., Sherwin, J.C., Wood, D., Mountain, J.A., et al. (2013). Raine eye health study: design, methodology and baseline prevalence of ophthalmic disease in a birth-cohort study of young adults. Ophthalmic genetics 34, 199-208.
54. Hofman, A., van Duijn, C.M., Franco, O.H., Ikram, M.A., Janssen, H.L., Klaver, C.C., Kuipers, E.J., Nijsten, T.E., Stricker, B.H., Tiemeier, H., et al. (2011). The Rotterdam Study: 2012 objectives and design update. Eur J Epidemiol 26, 657-686.
55. Spector, T.D., and Williams, F.M. (2006). The UK Adult Twin Registry (TwinsUK). Twin Res Hum Genet 9, 899-906.
56. Klein, R., Lee, K.E., Gangnon, R.E., and Klein, B.E.K. (2010). The 25-Year Incidence of Visual Impairment in Type 1 Diabetes Mellitus The Wisconsin Epidemiologic Study of Diabetic Retinopathy. Ophthalmology 117, 63-70.
57. Raitakari, O.T., Juonala, M., Ronnemaa, T., Keltikangas-Jarvinen, L., Rasanen, L., Pietikainen, M., Hutri-Kahonen, N., Taittonen, L., Jokinen, E., Marniemi, J., et al. (2008). Cohort profile: the cardiovascular risk in Young Finns Study. Int J Epidemiol 37, 1220-1226.
58. Smith, E.N., Chen, W., Kahonen, M., Kettunen, J., Lehtimaki, T., Peltonen, L., Raitakari, O.T., Salem, R.M., Schork, N.J., Shaw, M., et al. (2010). Longitudinal genome-wide association of cardiovascular disease risk factors in the Bogalusa heart study. PLoS Genet 6.
59. Cornes, B.K., Khor, C.C., Nongpiur, M.E., Xu, L., Tay, W.T., Zheng, Y., Lavanya, R., Li, Y., Wu, R., Sim, X., et al. (2012). Identification of four novel variants that influence central corneal thickness in multi-ethnic Asian populations. Hum Mol Genet 21, 437-445.
60. Nakata, I., Yamashiro, K., Nakanishi, H., Akagi-Kurashige, Y., Miyake, M., Tsujikawa, A., Matsuda, F., Yoshimura, N., and Nagahama Cohort Research, G. (2013). Prevalence and characteristics of age-related macular degeneration in the Japanese population: the Nagahama study. American journal of ophthalmology 156, 1002-1009 e1002.
61. Foong, A.W., Saw, S.M., Loo, J.L., Shen, S., Loon, S.C., Rosman, M., Aung, T., Tan, D.T., Tai, E.S., and Wong, T.Y. (2007). Rationale and methodology for a population-based study of eye diseases in Malay people: The Singapore Malay eye study (SiMES). Ophthalmic epidemiology 14, 25-35.
62. Vithana, E.N., Aung, T., Khor, C.C., Cornes, B.K., Tay, W.T., Sim, X., Lavanya, R., Wu, R., Zheng, Y., Hibberd, M.L., et al. (2011). Collagen-related genes influence the glaucoma risk factor, central corneal thickness. Human molecular genetics 20, 649-658.
63. Khor, C.C., Ramdas, W.D., Vithana, E.N., Cornes, B.K., Sim, X., Tay, W.T., Saw, S.M., Zheng, Y., Lavanya, R., Wu, R., et al. (2011). Genome-wide association studies in Asians confirm the involvement of ATOH7 and TGFBR3, and further identify CARD10 as a novel locus influencing optic disc area. Human molecular genetics 20, 1864-1872.
64. Lavanya, R., Jeganathan, V.S., Zheng, Y., Raju, P., Cheung, N., Tai, E.S., Wang, J.J., Lamoureux, E., Mitchell, P., Young, T.L., et al. (2009). Methodology of the Singapore Indian Chinese Cohort (SICC) eye study: quantifying ethnic variations in the epidemiology of eye diseases in Asians. Ophthalmic epidemiology 16, 325-336.
65. Hughes, K., Aw, T.C., Kuperan, P., and Choo, M. (1997). Central obesity, insulin resistance, syndrome X, lipoprotein(a), and cardiovascular risk in Indians, Malays, and Chinese in Singapore. J Epidemiol Community Health 51, 394-399.
66. Sim, X., Ong, R.T., Suo, C., Tay, W.T., Liu, J., Ng, D.P., Boehnke, M., Chia, K.S., Wong, T.Y., Seielstad, M., et al. (2011). Transferability of type 2 diabetes implicated loci in multi-ethnic cohorts from Southeast Asia. PLoS genetics 7, e1001363.

67. Li, L.J., Cheung, C.Y., Gazzard, G., Chang, L., Mitchell, P., Wong, T.Y., and Saw, S.M. (2011). Relationship of ocular biometry and retinal vascular caliber in preschoolers. Investigative ophthalmology & visual science 52, 9561-9566.
